# Synthesis and preliminary studies of ^11^C-labeled tetrahydro-1,7-naphthyridine-2-carboxamides for PET imaging of metabotropic glutamate receptor 2

**DOI:** 10.7150/thno.42587

**Published:** 2020-09-14

**Authors:** Xiaofei Zhang, Yiding Zhang, Zhen Chen, Tuo Shao, Richard Van, Katsushi Kumata, Xiaoyun Deng, Hualong Fu, Tomoteru Yamasaki, Jian Rong, Kuan Hu, Akiko Hatori, Lin Xie, Qingzhen Yu, Weijian Ye, Hao Xu, Douglas J. Sheffler, Nicholas D. P. Cosford, Yihan Shao, Pingping Tang, Lu Wang, Ming-Rong Zhang, Steven H. Liang

**Affiliations:** 1 Division of Nuclear Medicine and Molecular Imaging, Massachusetts General Hospital & Department of Radiology, Harvard Medical School, Boston, MA, 02114, USA; 2 State Key Laboratory and Institute of Elemento-Organic Chemistry, Collaborative Innovation Center of Chemical Science and Engineering, Nankai University, Tianjin 300071, China; 3 Department of Radiopharmaceuticals Development, National Institute of Radiological Sciences, National Institutes for Quantum and Radiological Science and Technology, 4-9-1 Anagawa, Inage-ku, Chiba 263-8555, Japan; 4 Department of Chemistry and Biochemistry, University of Oklahoma, Norman, Oklahoma 73019, United States; 5 Department of Nuclear Medicine and PET/CT-MRI Center, The First Affiliated Hospital of Jinan University, 613 West Huangpu Road, Tianhe District, Guangzhou 510630, China; 6 Cancer Metabolism and Signaling Networks Program and Conrad Prebys Center for Chemical Genomics, Sanford-Burnham Prebys Medical Discovery Institute, La Jolla, California 92037, United States.

**Keywords:** positron emission tomography, metabotropic glutamate receptor 2, negative allosteric modulator, 11C, mGlu2

## Abstract

Selective modulation of metabotropic glutamate receptor 2 (mGlu_2_) represents a novel therapeutic approach for treating brain disorders, including schizophrenia, depression, Parkinson's disease (PD), Alzheimer's disease (AD), drug abuse and addiction. Imaging mGlu_2_ using positron emission tomography (PET) would allow for* in vivo* quantification under physiological and pathological conditions and facilitate drug discovery by enabling target engagement studies. In this paper, we aimed to develop a novel specific radioligand derived from negative allosteric modulators (NAMs) for PET imaging of mGlu_2_.

**Methods.** A focused small molecule library of mGlu_2_ NAMs with tetrahydro naphthyridine scaffold was synthesized for pharmacology and physicochemical evaluation. GIRK dose-response assays and CNS panel binding selectivity assays were performed to study the affinity and selectivity of mGlu_2_ NAMs, among which compounds **14a** and **14b** were selected as PET ligand candidates. Autoradiography in SD rat brain sections was used to confirm the *in vitro* binding specificity and selectivity of [^11^C]**14a** and [^11^C]**14b** towards mGlu_2_. *In vivo* binding specificity was then studied by PET imaging. Whole body biodistribution study and radiometabolite analysis were conducted to demonstrate the pharmacokinetic properties of [^11^C]**14b** as most promising PET mGlu_2_ PET ligand.

**Results.** mGlu_2_ NAMs** 14a-14g** were synthesized in 14%-20% yields in five steps. NAMs **14a** and **14b** were selected to be the most promising ligands due to their high affinity in GIRK dose-response assays. [^11^C]**14a** and [^11^C]**14b** displayed similar heterogeneous distribution by autoradiography, consistent with mGlu_2_ expression in the brain. While PET imaging study showed good brain permeability for both tracers, compound [^11^C]**14b** demonstrated superior binding specificity compared to [^11^C]**14a**. Further radiometabolite analysis of [^11^C]**14b** showed excellent stability in the brain.

**Conclusions.** Compound** 14b** exhibited high affinity and excellent subtype selectivity, which was then evaluated by *in vitro* autoradiography and *in vivo* PET imaging study after labeling with carbon-11. Ligand [^11^C]**14b**, which we named [^11^C]MG2-1904, demonstrated high brain uptake and excellent *in vitro*/*in vivo* specific binding towards mGlu_2_ with high metabolic stability in the brain. As proof-of-concept, our preliminary work demonstrated a successful example of visualizing mGlu_2_ *in vivo* derived from NAMs, which represents a promising chemotype for further development and optimization aimed for clinical translation.

## Introduction

In the central nervous system (CNS), *L*-glutamate is the major endogenous neurotransmitter that mediates a vast majority of synaptic excitations by interacting with two distinct types of receptors: the ionotropic glutamate receptors (iGluRs), which have voltage-gated cation channel activity, and the metabotropic glutamate receptors (mGlus), which are coupled to GTP-binding proteins to mediate intracellular second messenger systems such as phospholipase C (PLC) and adenylate cyclase (AC) 1-7. Based on their pharmacology and signal transduction mechanism, mGlus consist of eight subtypes which are typically classified into three categories: Group I (mGlu_1_ and mGlu_5_) primarily activates PLC via G_αq_ coupling, leading to mobilization of intracellular Ca^2+^. Both group II (mGlu_2_ and mGlu_3_) and group III (mGlu_4_, mGlu_6-8_) preferentially couple to AC and G_αi_, inhibiting the release of AC and suppressing cAMP production 5. The mGlus modulate inhibitory activity within the CNS and the dysfunction of the glutamatergic system has been associated with the pathophysiology of numerous psychiatric and neurological disorders 8-11.

Group II mGlus negatively modulate the presynaptic release of glutamate and activation of potassium channels. As opposed to mGlu_3_ which is expressed throughout the CNS, mGlu_2_ has a more limited distribution and is localized extensively on presynaptic nerve terminals 8, 12. In the brain, high expression of mGlu_2_ can be found in the striatum, cerebral cortex, hippocampus, amygdala and cerebellum 13-17. It has been reported that mGlu_2_ dysfunction is related to several CNS disorders, thus attracting much attention as a promising therapeutic target 18, 19. In particular, selective modulation of mGlu_2_ is a potential strategy for the treatment of schizophrenia 20-22, depression 23, 24, Parkinson's disease (PD) 25-28, Alzheimer's disease (AD) 29, 30, drug abuse and addiction 31-35.

**Figure 1:**
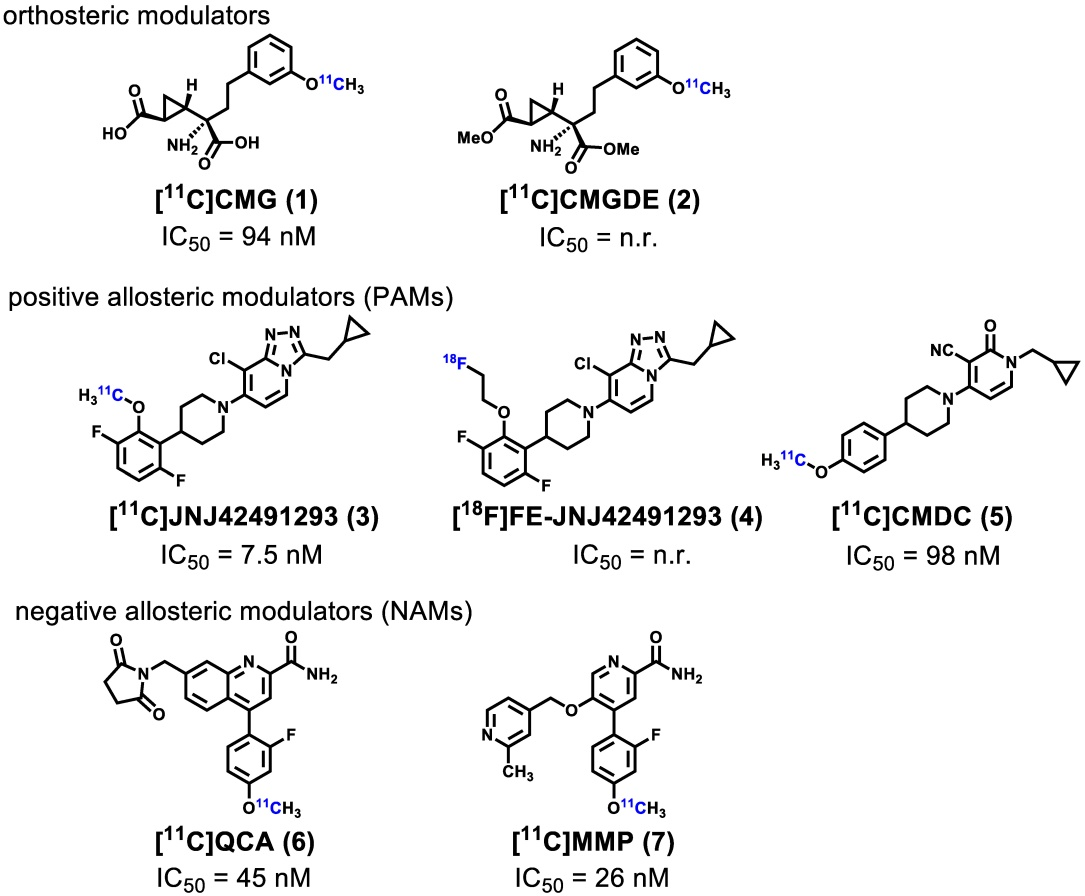
Representative mGlu2 PET ligands that have been tested in animals and/or humans. n.r. = not reported.

Noninvasive imaging of mGlu_2_ using positron emission tomography (PET) can visualize and provide quantitative measurement of the distribution and expression of this receptor under physiological and pathological conditions 36-40, further enabling a better understanding of the relationship between mGlu_2_ mediated glutamatergic signaling and CNS disorders. Furthermore, the development of high-affinity and selective mGlu_2_ PET tracers would enable clinical translation of mGlu_2_ modulators by providing a means to perform target engagement and dose occupancy studies. In the past several years, continuous research efforts have been invested in the development of PET ligands for imaging mGlu_2_, including [^11^C]CMG (**1**) 41, [^11^C]CMGDE (**2**) 41, [^11^C]JNJ42491293 (**3**) 42, [^18^F]FE-JNJ42491293 (**4**) 43, [^11^C]CMDC (**5**) 44, and our recently reported PET ligands namely [^11^C]QCA (**6**) 45 and [^11^C]MMP (**7**) 46 (**Figure 1**). As the first studied mGlu_2_ PET tracer, [^11^C]CMG (**1**) failed to cross blood-brain-barrier (BBB), which was then overcome by its ester derivative [^11^C]CMGDE (**2**) 41. These two tracers showed low *in vivo* specificity and selectivity between mGlu_2_ and mGlu_3_ attributed to the conserved orthosteric binding sites. The first positive allosteric modulator (PAM)-based PET ligands **3** 42 and its fluoroethyl analog **4** 43, were subsequently reported in 2012 and 2013, and the radioligand **3** was advanced to human studies 47. However, an unexpectedly high myocardial retention observed in humans along with off-target binding, confirmed by mGlu_2_ knockout rats, made these efforts of limited translational value. In addition, there are only limited preliminary mGlu_2_ imaging data in the human brain based on a mGlu_2_ ligand developed by Merck, Inc., the structure of which has not been disclosed 48. During the preparation of this manuscript, a preliminary radiosynthesis of a potential mGlu_2_ radioligand [^18^F]JNJ-46356479 has been reported 49. As a derivative of JNJ-40068782 50, [^11^C]CMDC (**5**) was not further pursued due to limited BBB penetration 44. In 2017, the first negative allosteric modulator (NAM)-based PET ligand, [^11^C]QCA (**6**) with good affinity (IC_50_ = 45 nM) and high selectivity for mGlu_2_ over mGlu_3_, was developed by our groups 45. Although *in vitro* autoradiography (ARG) results showed excellent specific binding to mGlu_2_, low brain uptake (peak value ~ 0.3 SUV) caused by ATP binding cassette (ABC) efflux pump (PgP/Bcrp substrate) limited further investigation of [^11^C]QCA (**6**). Most recently, our research groups identified a new NAM PET ligand namely [^11^C]MMP (**7**) with an improved affinity (IC_50_ = 26 nM) with reasonable lipophilicity (Log*D* = 3.30) 46. Unfortunately, animal PET imaging data demonstrated that [^11^C]MMP (**7**) exhibited moderate brain uptake (peak value ~ 0.6) and low levels of *in vivo* specific binding. To date, there is no NAM-based PET ligand that can visualize and quantify mGlu_2_ with sufficient brain penetration, high affinity and selectivity, and this represents an urgent and unmet need for drug discovery and clinical development.

In this study, we develop a focused array of NAMs with moderate to high affinity and selectivity for mGlu_2_ based on our continuous medicinal chemistry efforts. Herein, we describe our chemical syntheses, pharmacological screening and ^11^C-labeling of the most promising candidates. Comprehensive evaluations including brain permeability and specificity were conducted by *in vitro* autoradiography,* ex vivo* biodistribution and preliminary *in vivo* rodent PET imaging, all of which provide an excellent entry point for further mGlu_2_ PET ligand development aimed at clinical translation.

## Results and Discussion

### Medicinal Chemistry

**Scheme 1:**
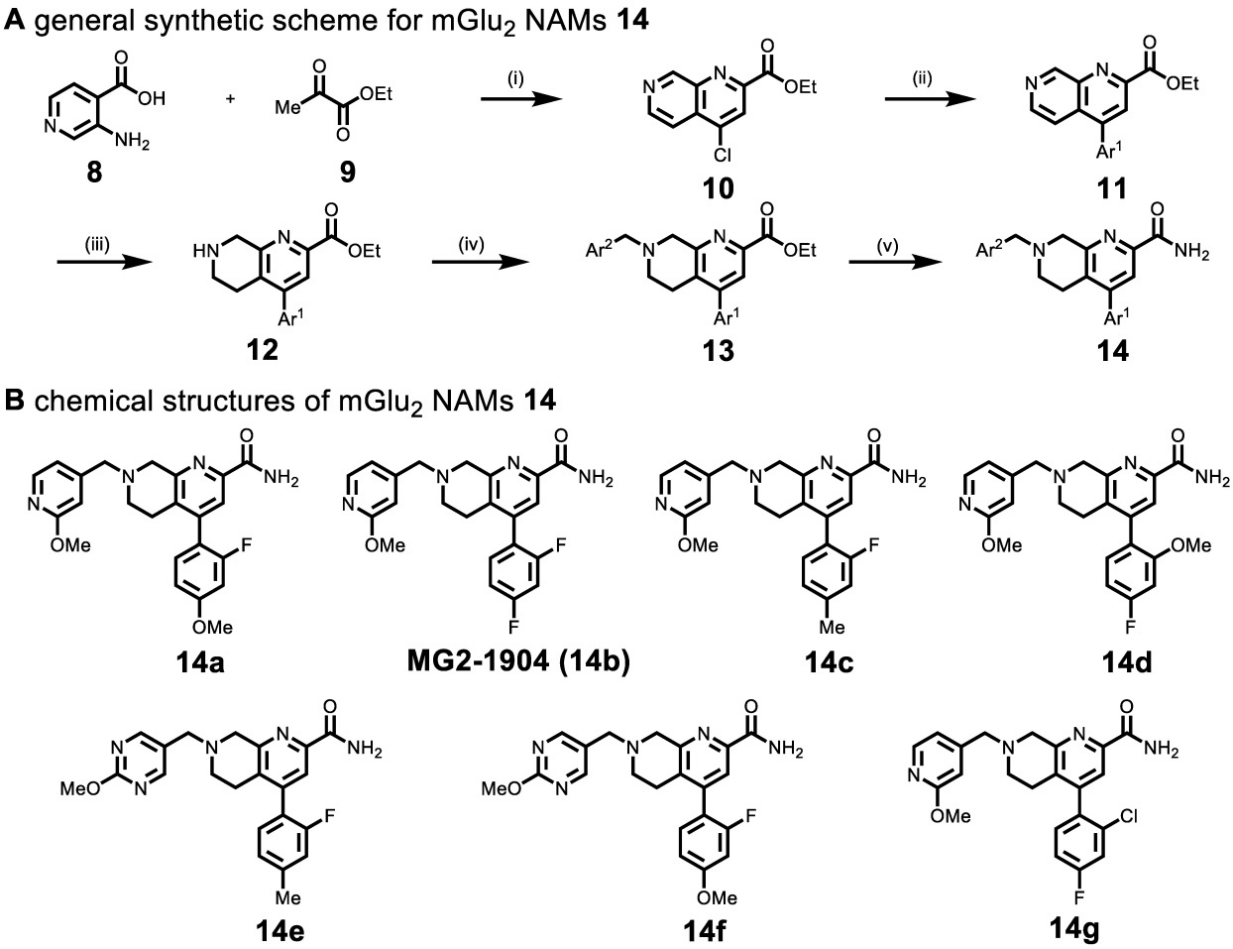
(A) Synthesis of mGlu_2_ NAMs **14a-14g**. Reactions and conditions: (i) POCl_3_, 100 ^o^C 1 h, 64% yield; (ii) Ar^1^-B(OH)_2_, Pd(PPh_3_)_4_, K_2_CO_3_, 1,4-Dioxane/H_2_O, 100 ^o^C, overnight, 80%-72% yield; (iii) NaBH_3_CN, AcOH, 25^o^C, 5 min; (iv) Ar^2^-CH_2_Cl,K_2_CO_3_, MeCN, r.t., 4 h, 52%-42% yield; (v) ammonia 7 M in MeOH, 25 ^o^C, 4 h, 80%-70% yield. (B) Chemical structures of mGlu_2_ NAMs **14a-14g**.

Based on recent advances in the development of mGlu_2_ NAMs (as therapeutic candidates) by scientists from Vanderbilt University^51^ and Merck Research Laboratories (Patents WO/2018/063955, WO/2016/032921, WO/2013/066736), a reductionist approach was utilized to design the second-generation of potent, selective and brain penetrant compounds. For PET ligand development, we envisioned that a simple tetrahydro naphthyridine scaffold with suitably tethered Ar^1^ and Ar^2^ moieties could overcome the challenges associated with brain penetration and *in vivo* stability encountered in the previous series (**Scheme 1**). The optimization was carried out by truncation of the bicyclic core system in the first generation QCA **6**. To obtain second-generation mGlu_2_ NAMs, we first employed acid-mediated cyclization between 3-aminoisonicotinic acid **8** and ethyl pyruvate **9** to synthesize naphthyridine ester **10** in 64% yield. Then aromatic motif Ar^1^ was installed via the Suzuki-Miyaura cross-coupling with different boronic acids in 72%-80% yields. Another aromatic moiety Ar^2^ was introduced by the reduction of naphthyridine moiety with NaBH_3_CN to provide tetrahydro naphthyridine **12**, followed by S_N_2 displacement with a series of heteroarylmethyl chlorides in 42%-52% yields over two steps. The ensuing compounds **13a-13g** were treated with ammonia in methanol to afford the desired mGlu_2_ NAMs **14a**-**14g** in 70-80% yields. Together, we synthesized seven second-generation mGlu_2_ NAMs from starting material **8** in 14%-20% overall yields in five steps.

**Figure 2:**
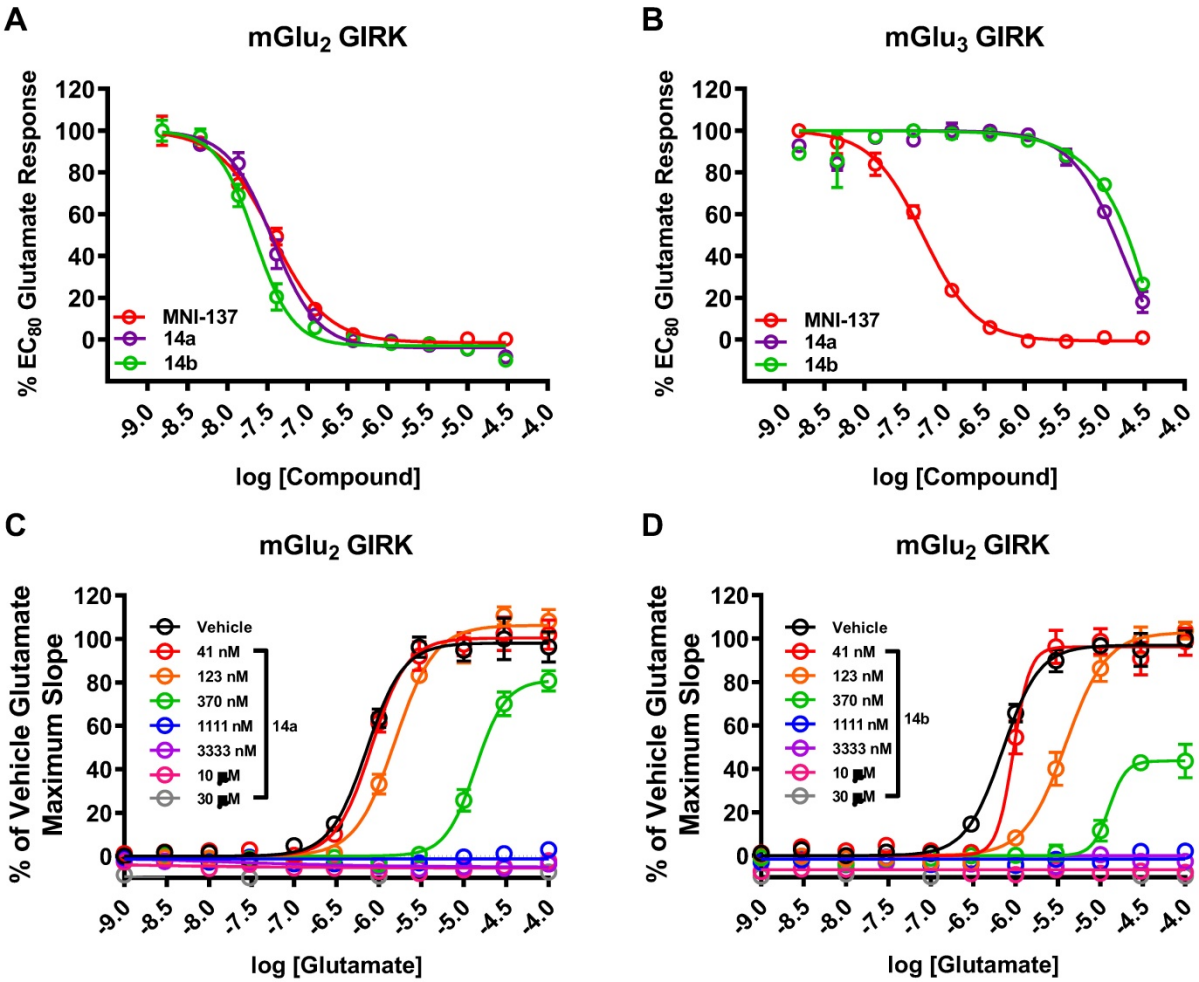
Representative dose-response curves of **14a** and **14b** in GIRK assays using human HEK293 cells expressing mGlu_2/3_ receptors. *In vitro* affinity of **14a** and **14b** with (A) mGlu_2_ GIRK assay and (B) mGlu_3_ GIRK assay with the control mGlu_2/3_ NAM MNI-137. As a non-competitive mode of action, **14a** (C) and **14b** (D) showed right shift of mGlu_2_ dose-response curves and decreased maximal response with increasing glutamate concentration.

The pharmacological and physicochemical properties of these NAMs were investigated using our established procedures 45, and the results are depicted in** Table 1**. Representative concentration-response curves for determining the affinity and subtype selectivity (**Figures 2A** & **2B**) as well as noncompetitive negative allosteric modulation (dose-dependently right shift and maximal response decrease towards increasing concentration of glutamate; **Figures 2C** & **2D**) of compounds **14a** and **14b** are illustrated herein. For affinity and subtype selectivity evaluation of all NAMs **14**, see details in **Figure S1 and S10** in the supporting information. Various substituents at Ar^1^ and Ar^2^ groups were incorporated into the scaffold of tetrahydro naphthyridine carboxamide. Interestingly, the optimal Ar^2^ group, namely 2-fluoro-4-methylphenyl, in the first-generation mGlu_2_ NAMs failed to improve the ligand affinity, as reflected by unfavored results of IC_50_ 129 nM for **14c**, and 106 nM for **14e**. Maintaining a 2-fluoro-4-mexothyphenyl group at the Ar^2^ position and changing the Ar^2^ group from pyridine to a pyrimidine moiety dramatically lowered affinity towards mGlu_2_ (IC_50_ 39 nM for **14a** *vs.* 318 nM for **14f**). Varying the substituents on the Ar^1^ group also affected the affinity. The IC_50_ values decreased when the methoxy group was displaced by a fluorine at the* para*-position (**14a** *vs.* **14b**), but increased when the fluoro substituent was displaced by methoxy or chloro substituents at the* ortho*-position (**14b** *vs.* **14d**, **14g**). All these NAMs showed excellent selectivity towards mGlu_2_ over mGlu_3_ (IC_50_ > 10-30 μM for mGlu_3_). Furthermore, we performed the binding selectivity assays of ligand **14b** in a comprehensive CNS panel provided by the NIMH PDSP. The results indicated that **14b** has no substantial interaction with related mGlu receptor family and no significant response with any other major brain targets. The compound also showed no activity in hERG safety assay (see details in **Figure S10** and **S11** in the supporting information). Compounds **14a-14g** were slightly more lipophilic than the first-generation ligands, and tPSA values for this series were reduced, all of which may provide improved brain permeability compared with **6**. As a result, NAMs **14a** and **14b** were selected to be the most promising ligands due to their high affinity (**Figure 2A**), excellent subtype selectivity (**Figure 2B**, inactive against mGlu_3_ up to 30 µM) and reasonable lipophilicity and tPSA in the second-generation. To our delight, methoxy substituents on these two compounds could be used as a labeling handle for carbon-11 52, which could facilitate our preliminary *in vivo* evaluation on this scaffold with PET.

### Radiochemistry

**Scheme 2:**
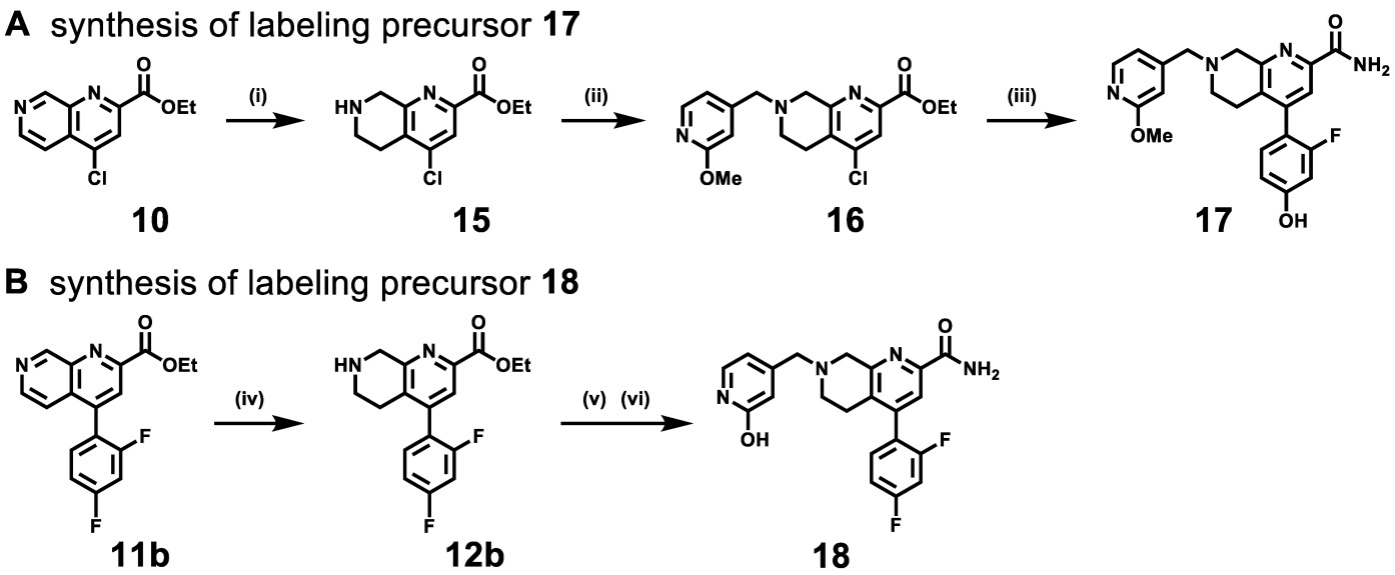
Syntheses of labeling precursors** 17** (A) and **18** (B). Reactions and conditions: (i) NaBH_3_CN, AcOH, 25 ℃, 5 min; (ii) 2-methoxy-4-(chloromethyl)pyridine, K_2_CO_3_, MeCN, r.t., 4 h, 45% yield for two steps; (iii) (2-fluoro-4-hydroxyphenyl)boronic acid, Pd(dppf)Cl_2_, H_2_O/1,4-dixone, K_2_CO_3_, 100 ℃, 5 h then ammonia 7 M in MeOH, 25 ℃, 4 h, 42% yield for two steps; (iv) NaBH_3_CN, AcOH, 25 ℃, 5 min; (v) K_2_CO_3_, MeCN, r.t., 4 h; (vi) ammonia 7M in MeOH, 25 ℃, 4 h, 70% yield.

The hydroxyl group on (hetero)arenes is a feasible labeling site for ^11^C-labeled PET ligand development via methylation with [^11^C]CH_3_I under basic conditions 52. As shown in **Scheme 2**, the syntheses of radiolabeling precursors **17** and **18** were conducted analogously to our synthetic route for second-generation mGlu_2_ NAMs. Compound **16** was obtained from intermediate **10** by NaBH_3_CN-mediated reduction and subsequent S_N_2 displacement with 2-methoxy-4-(chloromethyl)pyridine in 45% yield over two steps. The Suzuki cross-coupling with 2-fluoro-4-hydroxyphenylboronic acid and ammonolysis with 7N ammonia in methanol solution was employed to give radiolabeling precursor **17** in 44% yield over two steps. Amine alkylation between 2-hydroxy-4-(chloromethyl)pyridine and intermediate **12b** (reduced from compound **11b**) was conducted followed by ammonolysis fulfilled the synthesis of radiolabeling precursor **18** in 56% yield. In all, precursors **17** and **18** were synthesized from their corresponding 1,7-naphthyridine-2-carboxylates **10** and **11b** in an overall yield of 20% and 22%, respectively, over three steps.

**Scheme 3:**
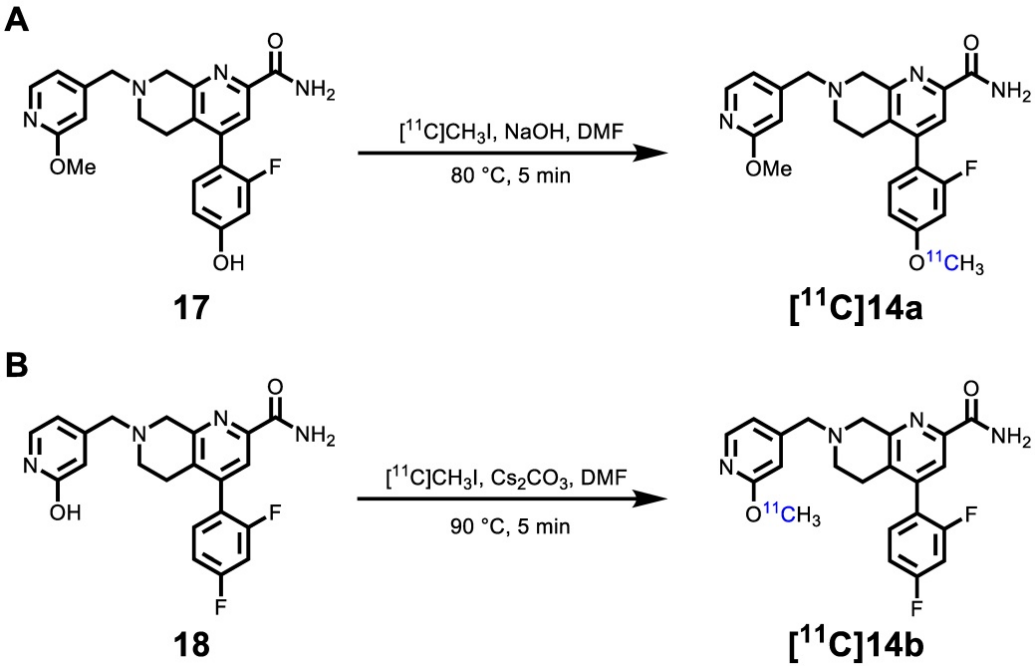
Radiosynthesis of [^11^C]**14a** (A) [^11^C]**14b** (B).

As shown in **Scheme 3A**, the radiosynthesis of [^11^C]**14a** was accomplished by passing gaseous [^11^C]CH_3_I through a solution of phenolic precursor **17** (1.0 mg) and NaOH (3-5 μL, 0.5 M) in DMF (300 μL). The reaction was conducted at 80 °C for 5 min, followed by purification using semi-preparative HPLC. The decay-corrected radiochemical yields of [^11^C]**14a** were 36.6 ± 7.3% (*n* = 7) based on the starting radioactivity of [^11^C]CO_2_. The radiosynthesis of [^11^C]**14b** was realized by ^11^C-methylation of hydroxypyridinyl precursor **18** (1.0 mg) in anhydrous DMF (300 μL; pre-saturated with 10-20 mg Cs_2_CO_3_) at 90 °C for 5 min (**Scheme 3B**), generating the desired product [^11^C]**14b** with decay-corrected radiochemical yields of 6.53 ± 1.5% (*n* = 10) after semipreparative HPLC purification. Both radiotracers were formulated in a saline solution containing 100 μL of 25% ascorbic acid in sterile water and 100 μL of 20% Tween 80 (see details in Methods). The radiochemical and chemical purities were greater than 99%, and molar activities were greater than 74 GBq/μmol (2 Ci/μmol). The overall synthesis time of both radiotracers was* ca*. 40 min, and no radiolysis was observed up to 90 min.

### *In Vitro* Autoradiography

**Figure 3:**
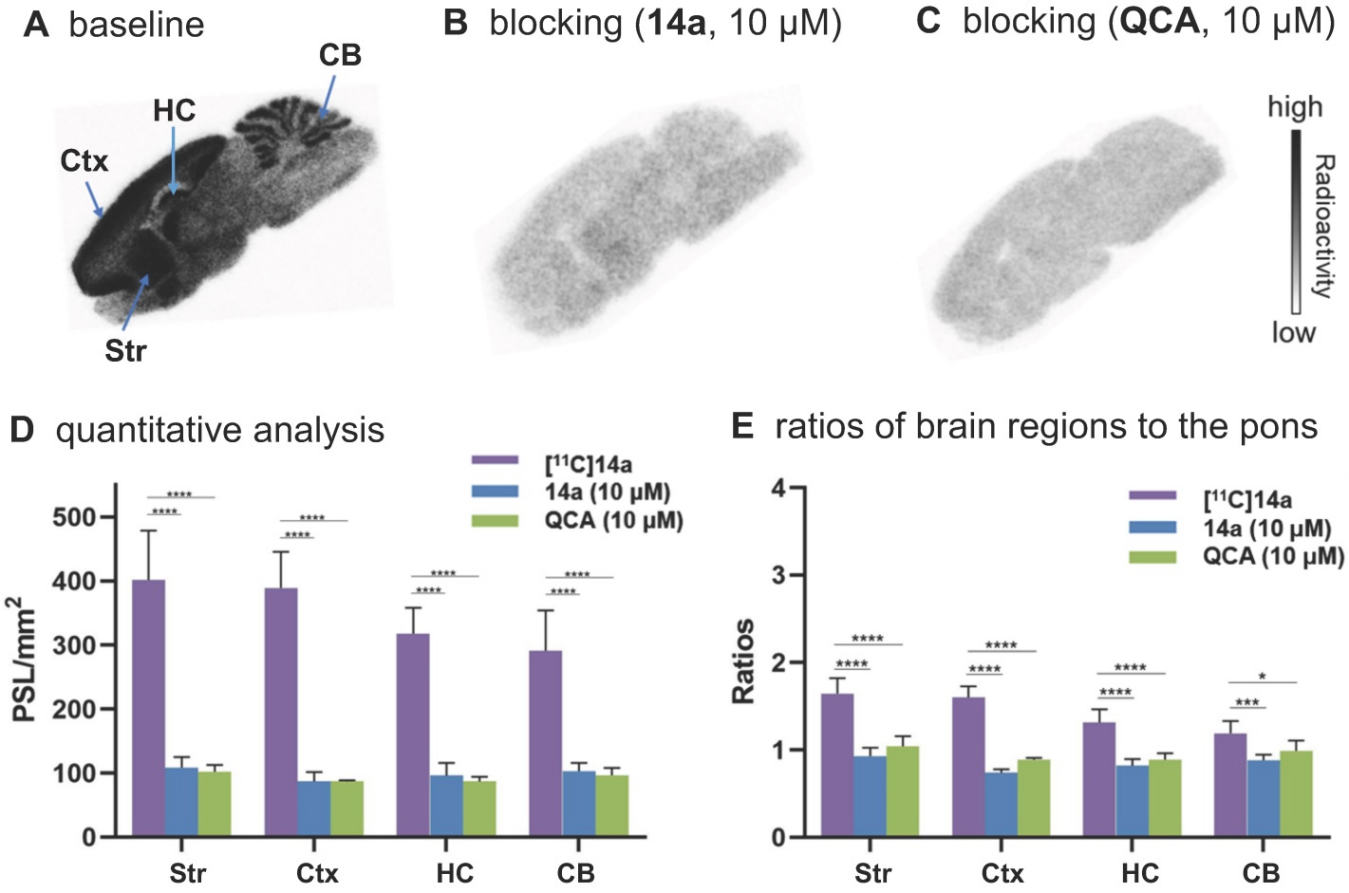
*In vitro* autoradiography of [^11^C]**14a** in rat brain sections. (A) Brain sections were treated with [^11^C]**14a** (1.25 μCi/mL); (B) Brain sections were pre-treated with **14a** (10 μM), followed by [^11^C]**14a** (1.25 μCi/mL); (C) Brain sections were pre-treated with **QCA** (10 μM), followed by [^11^C]**14a** (1.25 μCi/mL); (D) Quantitative analysis of baseline and blocking experiments. The value is expressed as PSL per mm^2^; (E) Ratios of brain regions to the pons. Str = striatum; Ctx = cerebral cortex; HC = hippocampus; CB = cerebellum. Six serial brain sections were used for each conditions. Data are presented as mean ± SEM (*n* = 6) and analyzed by one-way ANOVA. Asterisks indicate statistical significance. *p < 0.05, ***p ≤ 0.001, and ****p ≤ 0.0001.

**Figure 4:**
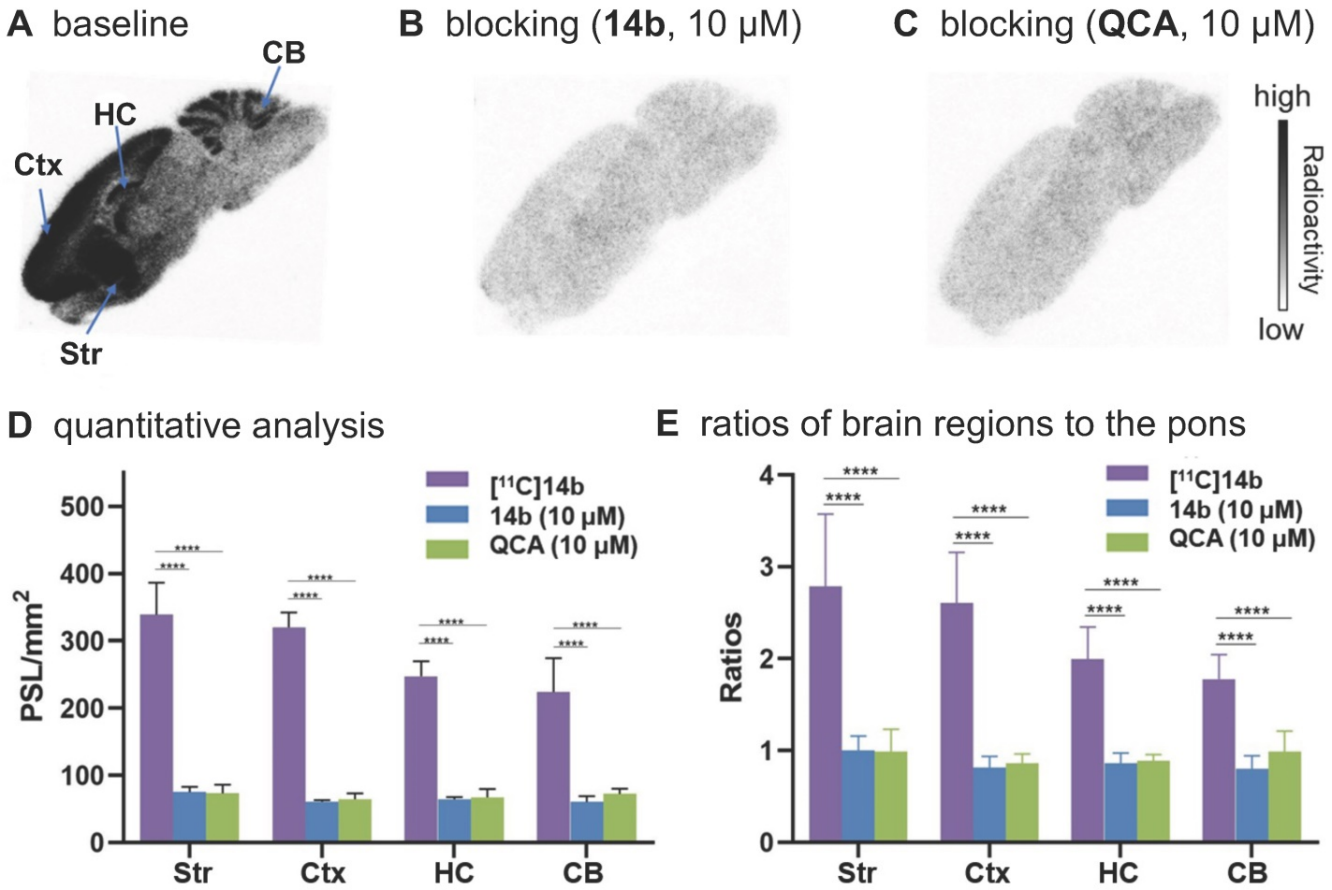
*In vitro* autoradiography of [^11^C]**14b** in rat brain sections. (A) Brain sections were treated with [^11^C]**21b** (1.25 μCi/mL); (B) Brain sections were pre-treated with **14b** (10 μM), followed by [^11^C]**21b** (1.25 μCi/mL); (C) Brain sections were pre-treated with **QCA** (10 μM), followed by [^11^C]**14b** (1.25 μCi/mL); (D) Quantitative analysis of baseline and blocking experiments. The value is expressed as PSL per mm^2^; (E) Ratios of brain regions to the pons. Str = striatum; Ctx = cerebral cortex; HC = hippocampus; CB = cerebellum. Six serial brain sections were used for each conditions. Data are presented as mean ± SEM (*n* = 6) and analyzed by one-way ANOVA. Asterisks indicate statistical significance. *p < 0.05, ***p ≤ 0.001, and ****p ≤ 0.0001.

To confirm the *in vitro* binding specificity and selectivity of [^11^C]**14a** and [^11^C]**14b** towards mGlu_2_,* in vitro* autoradiography (ARG) studies were performed in the brain sections of Sprague-Dawley (SD) rats wild-type (**Figures 3** &** 4**) and an image of the ROIs used for quantification is included in **Figure S9** in the supporting information. Both ligands [^11^C]**14a** and [^11^C]**14b** (radioconcentration 1.25 μCi/mL each) displayed similar heterogeneous distribution with high radioactivity accumulated in the striatum and cerebral cortex, followed by hippocampus and cerebellum (**Figure 3A** &** 3D**; **Figure 4A** &** 4D**). Their heterogeneous patterns were in accordance with the biological expression of mGlu_2_ in rodents 13-17, as well as our previously published data for [^11^C]QCA 45. Blocking studies were conducted by pretreatment with either the corresponding mGlu2 NAMs (10 μM of **14a** for [^11^C]**14a**, **Figure 3B**; 10 μM of **14b** for [^11^C]**14b**, **Figure 4B**) or QCA (10 μM of **6**, **Figure 3C** and **Figure 4C**), which showed substantial radioactivity reduction in rat brain sections (*vide infra*). These results indicated that both ligands [^11^C]**14a** and [^11^C]**14b** exhibited high-level specific binding towards mGlu_2_ *in vitro*. These ARG results were further analyzed by the comparison with bound signals from brain regions of interest to that of the pons, in which the lowest uptake was observed. As shown in **Figures 3E** &** 4E**, higher contrast ratios were observed for [^11^C]**14b**, for example, 2.79 in the striatum, 2.61 in the cerebral cortex, 2.00 in the hippocampus and 1.78 in the cerebellum under baseline conditions. Under blocking conditions (preincubated with unlabeled compound QCA, **14b** or **14a** at 10 µM, respectively), these ratios were decreased by 45-69% for [^11^C]**14b** while only 17-54% for [^11^C]**14a**. These results demonstrated that [^11^C]**14b** had improved specific binding to mGlu_2_ compared with [^11^C]**14a**. Attributed to their excellent binding specificity, both radioligands were advanced to subsequent PET evaluation to study their potential for imaging of mGlu_2_* in vivo*, although [^11^C]**14b** exhibited superior *in vitro* performance.

### PET Imaging Studies in Rat Brain

Dynamic PET imaging studies were performed in the brain of Sprague-Dawley (SD) rats to assess the *in vivo* binding specificity and washout kinetics of our promising radioligands [^11^C]**14a** and [^11^C]**14b**. As shown in **Figure S2A** (supporting information), time-activity curves (TACs) of [^11^C]**14a** exhibited good brain permeability. Specifically, the radioactivity in all brain regions of interest increased rapidly, peaked at 2.5 min (for example, 1.7 SUV in the striatum), and then gradually washed out over 60 min. However, pretreatment with non-radioactive compound **14a** (1 mg/kg) displayed marginal differences in major brain regions between baseline and blocking conditions (**Figure S2B** in supporting information), indicating low-to-modest *in vivo* specificity of [^11^C]**14a**.

**Figure 5:**
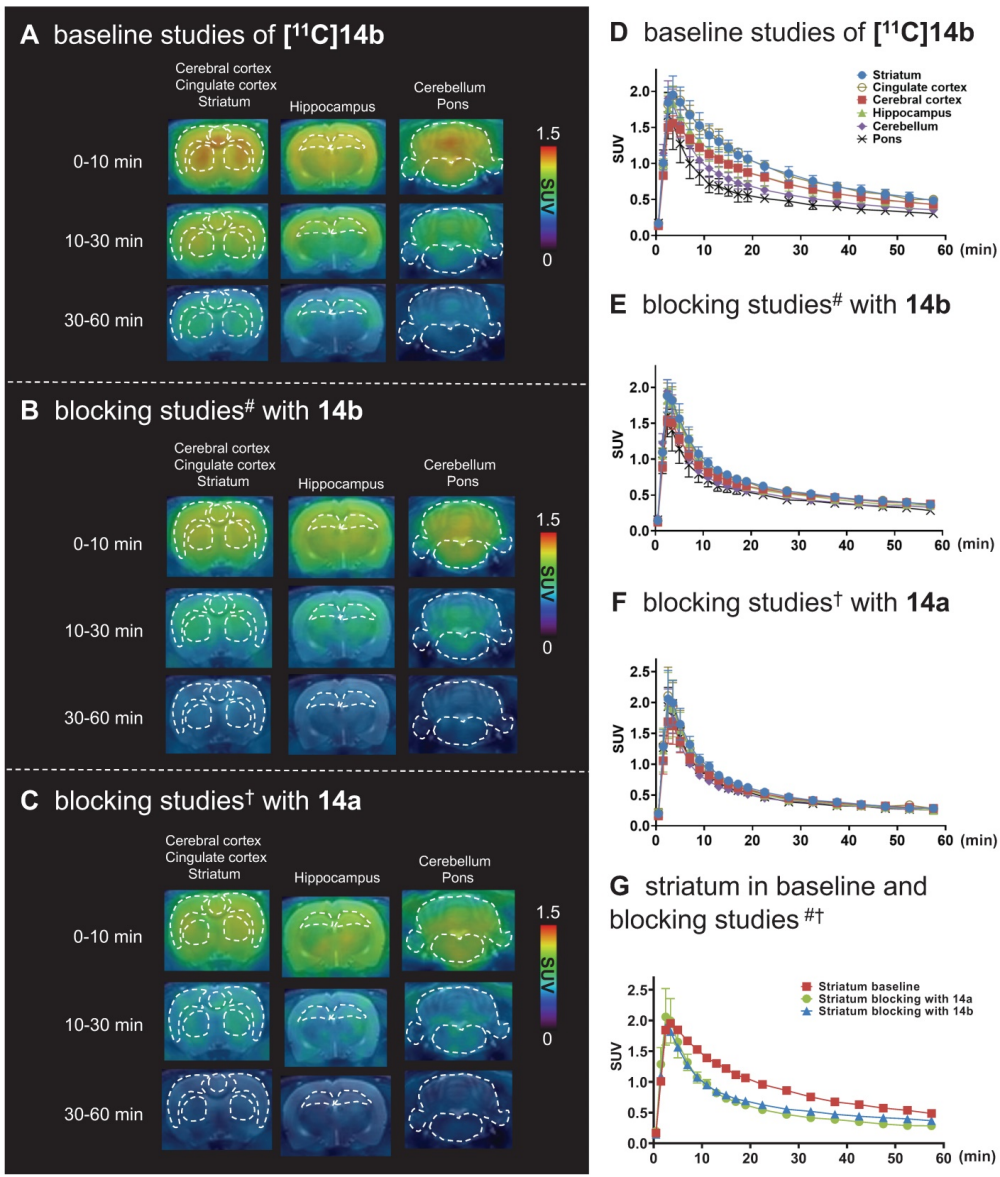
Representative PET/MRI fused coronal images (summed at 0-10 min, 10-30 min and 30-60 min) and time-activity curves of [^11^C]**14b** under baseline and blocking conditions in SD rat brain. ^#^Blocking conditions: **14b** (1 mg/kg), 30 min* i.v.* before radioligand injection; ^†^Blocking conditions: **14a** (3 mg/kg), 30 min *i.v.* before radioligand injection. Data are presented as mean ± SEM (n = 3).

For PET imaging of [^11^C]**14b** in rat brains, representative PET images (summed coronal view at 0-10 min, 10-30 min and 30-60 min intervals; see **Figure S12** for sagittal and horizontal views in the supporting information) in different brain regions, and the corresponding TACs are shown in **Figure 5**. The radioactivity in all brain regions reached a maximum level within 3 min, displayed heterogeneous distribution, which is consistent with mGlu_2_ distribution, and washed out gradually over 60 min (**Figure 5A** &** 5D**). Blocking studies with pretreatment of non-radioactive NAMs **14b** and **14a** successfully abolished heterogenous regional brain uptake, leading to a uniform distribution and reduced brain uptake (**Figure 5B, 5C, 5E** &** 5F**). Using the highest mGlu_2_ region, the striatum, as an example, we compared the regional TACs under baseline and blocking conditions in **Figure 5G** and found obvious reduction of the bound signals under blocking. These results indicated [^11^C]**14b** showed improved *in vivo* binding specificity to mGlu_2_ compared to [^11^C]**14a**.

**Figure 6:**
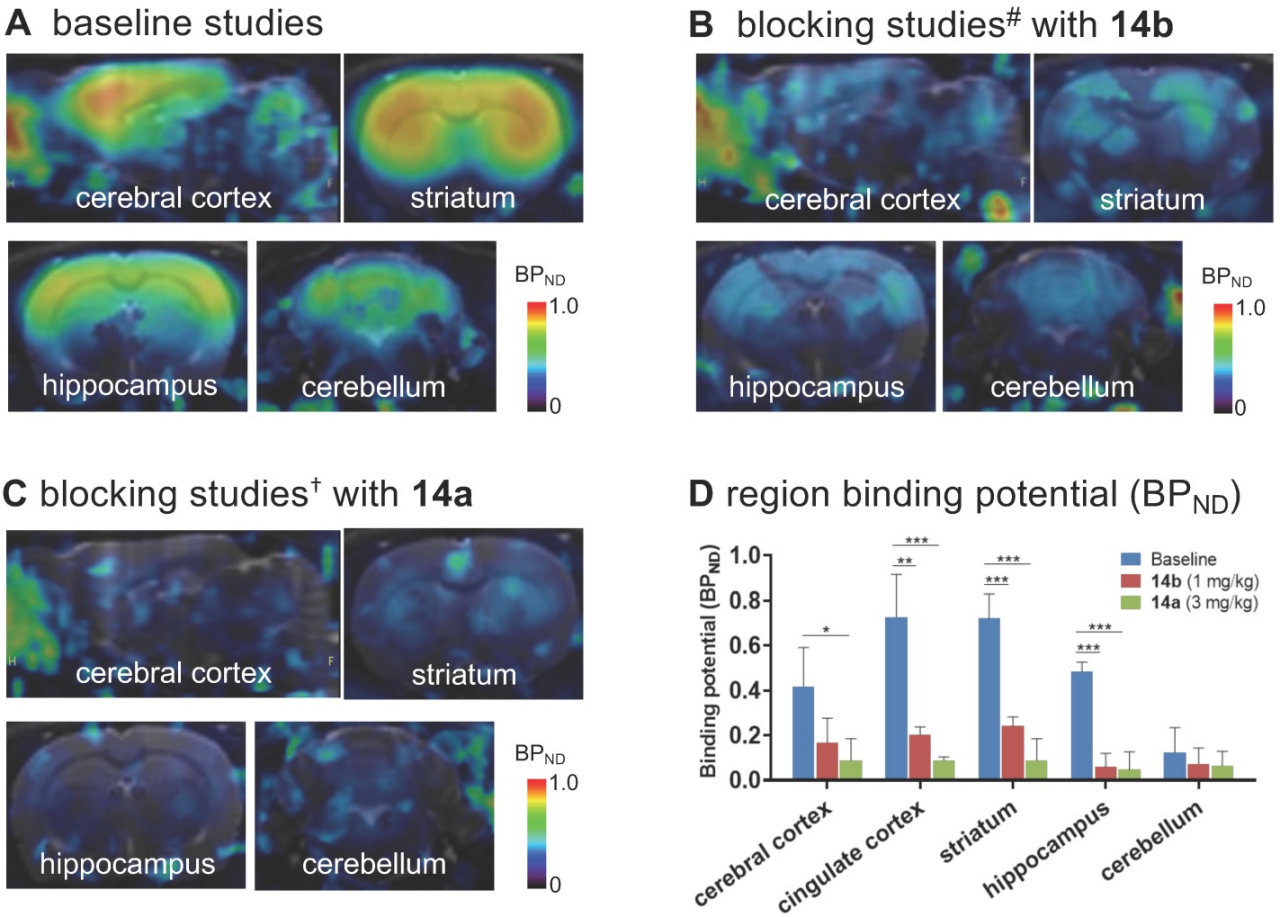
Parametric mapping and binding potentials of [^11^C]**14b** in rat brains. ^#^Blocking conditions: **14b** (1 mg/kg), 30 min *i.v.* before radioligand injection; ^†^blocking conditions: **14a** (3 mg/kg), 30 min *i.v.* before radioligand injection. Data are presented as mean ± SEM (*n* = 3) and analyzed by one-way ANOVA. Asterisks indicate statistical significance. *p < 0.05, **p ≤ 0.01, and ***p ≤ 0.001.

To further quantitatively estimate the specific binding of [^11^C]**14b**, the non-displaceable binding potential (BP_ND_) values were analyzed by the simplified reference tissue model (SRTM) 53, 54, with the pons as the pseudo reference region (the lowest and consistent brain uptake between baseline and blocking conditions). As shown in **Figure 6**, the bound signal of [^11^C]**14b** in rat brain was found in a decreasing order of the striatum and cingulate cortex (0.72), followed by the cerebral cortex (0.42) and hippocampus (0.48), and the lowest BP_ND_ was identified in the cerebellum (0.12) under baseline conditions. The distribution pattern was consistent with the expression of mGlu_2_ in rat brain 13-17 as well as the *in vitro* ARG results in **Figure 4**. Under blocking conditions, the BP_ND_ values were decreased substantially by pretreatment with **14b** (41-88% reduction, 1 mg/kg, **Figure 6B** and **6D**) and **14a** (48-90% reduction, 3 mg/kg, **Figure 6C** and** 6D**). The signal heterogeneity in the parametric brain mapping was also abolished under these conditions. In all, parametric PET images with the BP_ND_ scale could clearly visualize the uptake differences in detailed brain regions, and confirm the specific binding of [^11^C]**14b** *in vivo* between baseline and blocking conditions by PET.

### Whole Body Biodistribution Studies and Radiometabolite Analysis of [^11^C]14b

**Figure 7:**
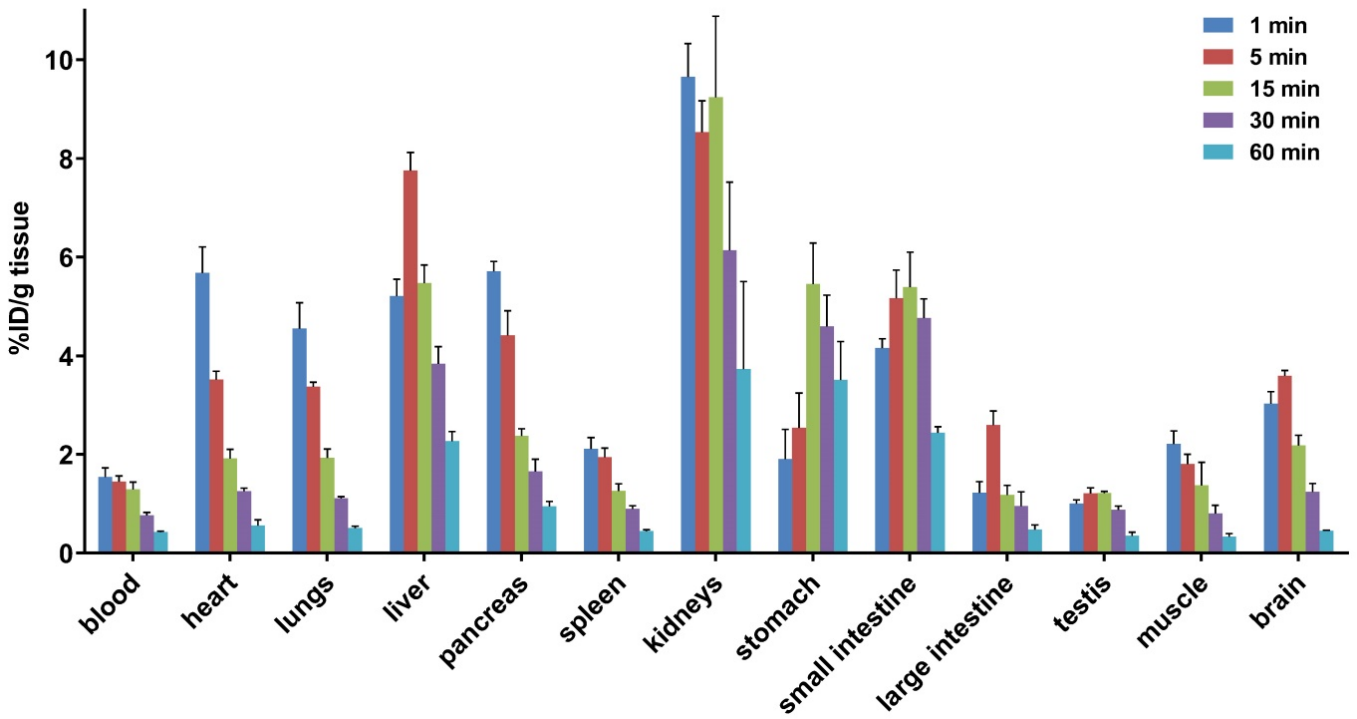
Whole-body *ex vivo* biodistribution studies in mice at five different time points (1, 5, 15, 30 and 60 min) post injection of [^11^C]**14b**. Data are expressed as %ID/g (mean ± SD, *n* = 3). %ID/g = injected dose per gram of wet tissue.

**Figure 8:**
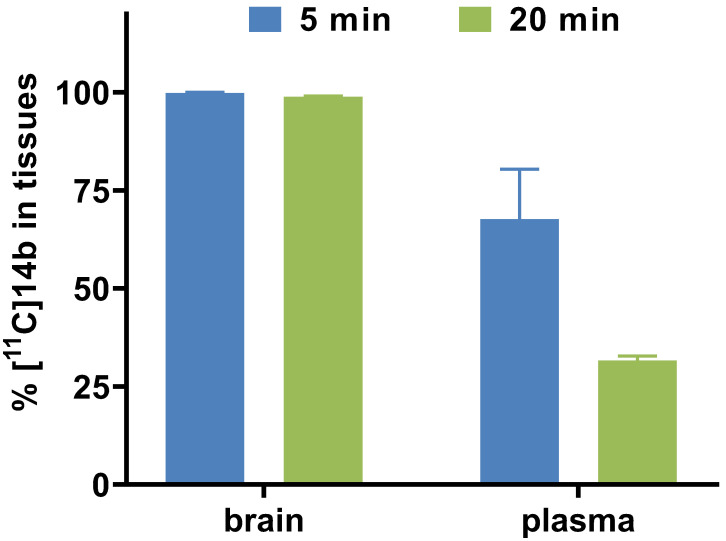
Radiometabolite analysis of [^11^C]**14b** in rats (average two runs)

To study the pharmacokinetic properties of [^11^C]**14b**, the uptake, distribution, and clearance were studied in mice at five time points (1, 5, 15, 30, and 60 min) post tracer injection. The results were expressed as the percentage of the injected dose per gram of wet tissue (%ID/g) in **Figure 7**. The radioactivity of [^11^C]**14b** in blood was washed out rapidly, with the ratio of %ID/g_(1min/60min)_ > 3.5. High radioactivity levels (> 4 %ID/g) were observed in several organs including the heart, lungs, liver, pancreas, kidneys and small intestine within the initial 1 min. After that, the signals in most organs decreased rapidly, while the radioactivity in the liver, large intestine and brain increased until 5 min and then washed out gradually. The signal in the stomach and small intestine reached a plateau after 15 min. The particular high uptake in the small intestine, kidney, and liver was probably due to the hepatobiliary and urinary excretion together with possible renal and intestinal reuptake pathways.^55^ Notably, high brain uptake (*ca.* 3.6 %ID/g) was detected after 5 min post injection, which was consistent with *in vivo* imaging data obtained from the PET studies. Furthermore, to investigate the *in vivo* stability of [^11^C]**14b**, radiometabolites in the plasma and brain in SD rats were evaluated at two time points (5 and 20 min) post injection. The percentages of parent [^11^C]**14b** and corresponding radiometabolites which were determined by radio-HPLC are shown in **Figure 8** (see details in **Figure S6-S8** and **Table S2** in the supporting information). The fraction corresponding to unchanged [^11^C]**14b** in plasma was decreased from 77% at 5 min to 21% at 20 min. On the other hand, [^11^C]**14b** showed excellent stability in the brain without any obvious ^11^C-labeled metabolite detected (unchanged fraction >99% at 20 min), which suggested the radiometabolites in the blood did not cross the BBB. It should be noted that, based on the radiometabolite analysis of [^11^C]**14a**, which has reasonable *in vivo* stability in the brain and plasma (see details in **Figure S3-S5** and **Table S1** in the supporting information), we have ruled out the possibility of high nonspecific binding of [^11^C]**14a** was attributed to *in vivo* metabolism. We postulated that the difference between IC_50_ values (39 nM of **14a** versus 24 nM of **14b**) and target selectivity may, in part, explain the increased nonspecific binding of [^11^C]**14a*** in vitro* (as seen in autoradiography) and *in vivo* (by PET study). Further in-depth pharmacological experiment is necessary to validate this hypothesis. As a result, the high *in vivo* stability of [^11^C]**14b** in the brain could facilitate further quantification of mGlu_2_ expression and target engagement studies by PET. Subsequent isotopologue-labeling of ligand **14b** using ^18^F, saturation binding assay to determine B_max_ and K_d_, and further validation including PET imaging in mGlu_2_ knockout mice and non-human primate are underway to evaluate the suitability of this new chemotype for potential clinical translation.

## Conclusion

We have prepared a new array of tetrahydro-1,7-naphthyridine carboxamide-based mGlu_2_ NAMs with amenability for radiolabeling. The preliminary pharmacological and physicochemical evaluations were carried out to identify two most promising modulators **14a** and **14b**, the corresponding ^11^C-isotopologues of which were produced in good radiochemical yields and high radiochemical purities. The subsequent autoradiography, PET imaging, whole body distribution and radiometabolism studies demonstrated that [^11^C]**14b** (which we named [^11^C]MG2-1904) exhibited sufficient brain permeability, high specific binding, and suitable *in vivo* metabolic stability, which could be used for further quantitative measurement under different physiological and pathological conditions. Further validation including PET imaging in higher species are underway to evaluate the suitability of this new chemotype for potential clinical translation.

## Materials and Methods

**General Considerations.** All the starting materials used in the syntheses were purchased from commercial vendors and used without further purification. Thin-layer chromatography (TLC) was conducted with 0.25 mm silica gel plates (^60^F_254_) and visualized by exposure to UV light (254 nm) or stained with potassium permanganate. Flash column chromatography was performed using silica gel (particle size 0.040-0.063 mm). ^1^H-Nuclear magnetic resonance (NMR) spectra were obtained on a 300 & 400 MHz on Bruker spectrometers. ^13^C NMR spectra were obtained at 75 & 100 MHz. Chemical shifts (δ) are reported in ppm and coupling constants are reported in Hertz. The multiplicities are abbreviated as follows: s = singlet, d = doublet, t = triplet, q = quartet, quint = quintet, sext = sextet, sept = setpet, m = multiplet, br = broad signal, dd = doublet of doublets. For all the HRMS measurements, the ionization method is ESI and the mass analyzer type is TOF on an AB SCIEX 500R Mass Spectrometer Systems. Lipophilicity (cLogD) and topological polar surface area (tPSA) were calculated by ChemDraw 16.0 software (PerkinElmer, USA). Carbon-11 (^11^C) was produced by ^14^N(p, α)^11^C nuclear reactions using a GE PETtrace cyclotron (16.5 MeV) or a Sumitomo CYPRIS HM-18 cyclotron. The animal experiments were approved by the Institutional Animal Care and Use Committee of Massachusetts General Hospital or the Animal Ethics Committee at the National Institute of Radiological Sciences. DdY mice (male; 7 weeks, 34-36 g) and SD rats (male; 7 weeks; 210-230 g) were kept on a 12 h light/12 h dark cycle and were allowed food and water ad libitum.

### Medicinal Chemistry

#### Chemical syntheses of mGlu_2_ NAMs 14

*Ethyl 4-chloro-1,7-naphthyridine-2-carboxylate* (**10**)*.* To 3-aminoisonicotinic acid (**8**) (5.24 g, 22.2 mmol) in a round-bottom flask was added ethyl pyruvate (**9**) (6 mL, 54.0 mmol, 2.4 equiv) and stirred for 10 mins before the addition of POCl_3_ (90 mL). The mixture was stirred at 100 ^o^C for 1 h, then quenched by iced water (100 mL) and 1 N NaOH (300 mL) before extracted with dichloromethane (200 mL, three times). The combined organic layers were washed with saturated aqueous sodium chloride, dried over MgSO_4_ and concentrated *in vacuo*. The residue was purified by flash chromatography on silica gel (hexanes to ethyl acetate gradient column) to yield the compound (**10**) as brown solid (3.35 g, 64%). R*_f_*= 0.3 (Hexanes/EtOAc = 10:1). ^1^H NMR (400 MHz, CDCl_3_) 9.75 (d, *J* = 0.9 Hz, 1H), 8.84 (d, *J* = 5.8 Hz, 1H), 8.47 (s, 1H), 8.08 (dd, *J* = 5.8, 1.0 Hz, 1H), 4.61 (q, *J* = 7.1 Hz, 2H), 1.53 (t, *J* = 7.1 Hz, 3H). ^13^C NMR (100 MHz, CDCl_3_) δ 163.8, 155.5, 149.7, 146.3, 143.3, 143.0, 130.8, 124.8, 116.1, 63.0, 14.3.

*Ethyl 4-(2-fluoro-4-methoxyphenyl)-1,7-naphthyridine-2-carboxylate* (**11a**)*.* To a solution of ethyl 4-chloro-1,7-naphthyridine-2-carboxylate (**10**) (70.8 mg, 0.300 mmol), 2-fluoro-4-methoxyphenylboronic acid (51.0 mg, 0.300 mmol) and K_2_CO_3_ (82.8 mg, 0.600 mmol) in 1,4-dioxane/water (v/v, 10/1, 1.8 mL) was added Pd(PPh_3_)_4_ (34.6 mg, 0.03 mmol) under Ar. The mixture was stirred at 100 ^o^C overnight, then quenched with water (3 mL) and extracted with ethyl acetate (5 mL, three times). The combined organic layers were washed with saturated aqueous sodium chloride, dried over MgSO_4_ and concentrated *in vacuo*. The residue was purified by flash chromatography on silica gel (hexanes to ethyl acetate gradient column) to yield the compound **11a** as white solid (72 mg, 74%). R*_f_* = 0.3 (Hexanes/EtOAc = 10:1). ^1^H NMR (300 MHz, CDCl_3_) δ 9.76 (s, 1H), 8.66 (d, *J* = 6.0 Hz, 1H), 8.35 (s, 1H), 7.67 (dd, *J* = 6.0, 2.7 Hz, 1H), 7.35 (t, *J* = 8.5 Hz, 1H), 7.00-6.77 (m, 2H), 4.59 (q, *J* = 7.1 Hz, 2H), 3.91 (s, 3H), 1.51 (t, *J* = 7.1 Hz, 3H). ^13^C NMR (100 MHz, CDCl_3_) δ 164.8 , 164.5 (d, *J* = 249.1 Hz), 157.9 (d, *J* = 10.0 Hz), 155.6 , 149.4 , 145.8 , 145.0 , 142.7 , 132.0 (d, *J* = 10.2 Hz), 128.5 (d, *J* = 12.1 Hz), 125.7 , 118.3 , 107.7 (d, *J* = 21.7 Hz), 99.7 (d, *J* = 26.0 Hz), 62.6 , 55.8 , 14.4.

*Ethyl 4-(2,4-difluorophenyl)-1,7-naphthyridine-2-carboxylate* (**11b**). Compound **11b** was prepared in 80% yield as a white solid using a similar method that described for **11a**. ^1^H NMR (300 MHz, CDCl_3_) δ 9.79 (d, *J* = 0.9 Hz, 1H), 8.69 (d, *J* = 5.9 Hz, 1H), 8.35 (s, 1H), 7.58 (d, *J* = 5.9 Hz, 1H), 7.44 (td, *J* = 8.3, 6.2 Hz, 1H), 7.17 - 7.03 (m, 2H), 4.60 (q, *J* = 7.1 Hz, 2H), 1.52 (t, *J* = 7.1 Hz, 3H). ^13^C NMR (75 MHz, CDCl_3_) δ 164.4, 163.7 (dd, *J* = 252.7, 11.7 Hz), 159.7 (dd, *J* = 252.2, 12.1 Hz), 155.7, 149.4, 145.6, 142.6, 142.4, 132.4 (dd, *J* = 9.8, 4.4 Hz), 131.6 (d, *J* = 10.8 Hz), 130.8, 128.5 (d, *J* = 12.7 Hz), 125.7, 119.5 (dd, *J* = 15.7, 4.0 Hz), 117.5 (d, *J* = 2.0 Hz), 112.3 (dd, *J* = 21.5, 3.8 Hz), 104.8 (t, *J* = 25.6 Hz), 62.6, 14.3.

*Ethyl 4-(2-fluoro-4-methyphenyl)-1,7-naphthyridine-2-carboxylate* (**11c**). Compound **11c** was prepared in 72% yield as a white solid using a manner method that described for **11a**. ^1^H NMR (300 MHz, CDCl_3_) δ 9.72 (s, 1H), 8.61 (d, *J* = 5.8 Hz, 1H), 8.29 (d, *J* = 0.6 Hz, 1H), 7.55 (ddd, *J* = 5.9, 2.6, 1.0 Hz, 1H), 7.27 (t, *J* = 7.7 Hz, 1H), 7.17 - 7.01 (m, 2H), 4.54 (q, *J* = 7.1 Hz, 2H), 2.43 (s, 3H), 1.45 (t, *J* = 7.1 Hz, 3H). ^13^C NMR (75 MHz, CDCl_3_) δ 164.6, 159.2 (d, *J* = 249.0 Hz), 155.6, 149.3, 145.3, 143.6, 142.7, 142.5 (d, *J* = 8.0 Hz), 134.8 (d, *J* = 10.4 Hz), 131.1, 131.0, 131.0, 125.6, 125.5, 125.5, 120.2 (d, *J* = 15.5 Hz), 117.9, 116.9, 116.6, 62.6, 21.3 (d, *J* = 1.7 Hz), 14.3.

*Ethyl 4-(4-fluoro-2-methoxyphenyl)-1,7-naphthyridine-2-carboxylate* (**11d**). Compound **11d** was prepared in 80% yield as a white solid using a similar method that described for **11a**. ^1^H NMR (300 MHz, CDCl_3_) δ 9.75 (s, 1H), 8.61 (d, *J* = 6.0 Hz, 1H), 8.30 (s, 1H), 7.54 (d, *J* = 5.9 Hz, 1H), 7.26 (s, 3H), 7.00 - 6.66 (m, 2H), 4.59 (q, *J* = 7.1 Hz, 2H), 3.72 (s, 3H), 1.50 (t, *J* = 7.1 Hz, 3H). ^13^C NMR (100 MHz, CDCl_3_) δ 164.8, 164.5 (d, *J* = 249.1 Hz), 157.9 (d, *J* = 10.1 Hz), 155.6, 149.4, 145.8, 145.0, 142.7, 138.4 (d, *J* = 10.2 Hz), 132.0 (d, *J* = 10.2 Hz), 131.6, 128.5 (d, *J* = 12.1 Hz), 125.7, 120.6, 118.3, 107.7 (d, *J* = 21.7 Hz), 99.7 (d, *J* = 26.0 Hz), 62.6, 55.8, 14.4.

*Ethyl 4-(2-chloro-4-fluorophenyl)-1,7-naphthyridine-2-carboxylate* (**11g**). Compound **11g** was prepared in 75% yield as a white solid using a similar method that described for **11a**.^ 1^H NMR (400 MHz, CDCl_3_) δ 9.8 (s, 1H), 8.7 (d, *J* = 5.8 Hz, 1H), 8.3 (s, 1H), 7.4 - 7.3 (m, 3H), 7.2 (td, *J* = 8.2, 2.5 Hz, 1H), 4.6 (q, *J* = 7.5 Hz, 2H), 1.5 (t, *J* = 7.1 Hz, 3H).^ 13^C NMR (100 MHz, CDCl_3_) δ 164.6, 163.0 (d, *J* = 253.1 Hz), 155.8, 149.4, 145.7, 145.6, 142.6, 134.2 (d, *J* = 10.5 Hz), 132.4 (d, *J* = 9.0 Hz), 131.0, 130.8 (d, *J* = 3.7 Hz), 125.6, 117.7 (d, *J* = 24.8 Hz), 117.6, 114.8 (d, *J* = 21.4 Hz), 62.8, 14.4.

*Ethyl 4-(2-fluoro-4-methoxyphenyl)-4a,5,6,7,8,8a-hexahydro-1,7-naphthyridine-2-carboxylate* (**12a**)*.* To the solution of **11a** (5.0 mmol) in AcOH (10 mL) was added NaBH_3_CN (0.94 g, 15.0 mmol, 3.0 equiv). The mixture was stirred for 5 mins at room temperature, then quenched by water (30 mL) and extracted with dichloromethane (200 mL, 3 times). The combined organic layers were washed with saturated aqueous sodium chloride, dried over MgSO_4_ and concentrated *in vacuo*. The residue **12a** was used without further purification.

Compound **12b-g** were prepared in a manner similar to that described for **12a** and used without further purification.

*Ethyl 4-(2-fluoro-4-methoxyphenyl)-7-((2-methoxypyridin-4-yl)methyl)-5,6,7,8-tetrahydro-1,7-naphthyridine-2-carboxylate* (**13a**)*.* To the residue **12a** solution in MeCN (5 mL) was added K_2_CO_3_ (1.4 g, 10.0 mmol) before the addition of 4-(chloromethyl)-2-methoxypyridine (0.79 g, 5.0 mmol) in MeCN (5 mL). The mixture was stirred at room temperature for 4 h, then quenched with H_2_O and extracted with ethyl acetate (5 mL, three times). The combined organic layers were washed with saturated aqueous sodium chloride, dried over MgSO_4_ and concentrated *in vacuo*. The residue was purified by flash chromatography on silica gel (hexanes to ethyl acetate gradient column) to yield compound **13a** as white solid (50% for two steps, 67.7 mg). R*_f_* = 0.2 (Hexanes/EtOAc = 1:1). ^1^H NMR (300 MHz, CDCl_3_) δ 8.00 (d, *J* = 5.2 Hz, 1H), 7.76 (s, 1H), 7.06 (t, *J* = 8.5 Hz, 1H), 6.83 (d, *J* = 5.2 Hz, 1H), 6.76-6.58 (m, 3H), 4.36 (q, *J* = 7.1 Hz, 2H), 3.83 (s, 3H), 3.80 (s, 2H), 3.75 (s, 3H), 3.58 (s, 2H), 2.76-2.56 (m, 4H), 1.30 (t, *J* = 7.1 Hz, 3H). ^13^C NMR (100 MHz, CDCl_3_) δ 165.3, 164.6, 161.4 (d, *J* = 10.9 Hz), 159.8 (d, *J* = 247.5 Hz), 155.6, 149.9, 146.8, 145.1 (d, *J* = 53.5 Hz), 133.1, 132.1 (d, *J* = 9.9 Hz), 131.1 (d, *J* = 5.3 Hz), 128.5 (d, *J* = 12.1 Hz), 124.7, 117.3, 110.6, 110.4 (d, *J* = 2.9 Hz), 101.9 (d, *J* = 25.7 Hz), 61.8, 61.2, 58.9, 55.7, 53.4, 49.9, 27.2, 14.4.

*Ethyl 4-(2,4-difluorophenyl)-7-((2-methoxypyridin-4-yl)methyl)-5,6,7,8-tetrahydro-1,7-naphthyridine-2-carboxylate* (**13b**)*.* Compound **13b** was prepared in 45% yield as a white solid using a similar method that described for **13a**. ^1^H NMR (300 MHz, CDCl_3_) δ 8.09 (dd, *J* = 5.3, 0.7 Hz, 1H), 7.82 (s, 1H), 7.21 (td, *J* = 8.3, 6.3 Hz, 1H), 7.08 - 6.87 (m, 3H), 6.76 (dt, *J* = 1.4, 0.7 Hz, 1H), 4.44 (qd, *J* = 7.1, 0.6 Hz, 2H), 3.91 (d, *J* = 0.7 Hz, 3H), 3.89 (s, 2H), 3.68 (s, 2H), 2.73 (s, 4H), 1.38 (td, *J* = 7.1, 0.6 Hz, 3H). ^13^C NMR (75 MHz, CDCl_3_) δ 165.1, 164.6, 155.7, 146.9, 145.5, 144.0, 132.8, 131.6, 131.5 (dd, *J* = 9.5, 5.0 Hz), 124.9, 124.5, 121.4, 117.3, 111.9 (d, *J* = 21.4 Hz), 110.7, 104.5 (t, *J* = 25.5 Hz), 61.9, 61.1, 58.7, 53.4, 49.8, 27.0, 14.3.

*Ethyl 4-(2-fluoro-4-methyphenyl)-7-((2-methoxypyridin-4-yl)methyl)-5,6,7,8-tetrahydro-1,7-naphthyridine-2-carboxylate* (**13c**)*.* Compound **13c** was prepared in 52% yield as a white solid using a similar method that described for **13a**. ^1^H NMR (300 MHz, CDCl_3_) δ 8.09 (dd, *J* = 5.3, 0.7 Hz, 1H), 7.83 (s, 1H), 7.16-6.89 (m, 5H), 6.76 (s, 1H), 4.44 (q, *J* = 7.1 Hz, 2H), 3.92 (s, 3H), 3.89 (s, 2H), 3.68 (s, 2H), 2.74 (dd, *J* = 11.7, 4.9 Hz, 4H), 2.40 (s, 3H), 1.38 (t, *J* = 7.1 Hz, 3H). ^13^C NMR (75 MHz, CDCl_3_) δ 165.1, 164.6, 158.9 (d, *J* = 247.4 Hz), 147.6, 146.8, 145.3, 145.0, 141.4 (d, *J* = 7.9 Hz), 132.8, 130.2 (d, *J* = 3.8 Hz), 125.1 (d, *J* = 3.1 Hz), 124.5, 122.1, 117.2, 116.4 (d, *J* = 21.6 Hz), 110.6, 109.3, 61.8, 61.1, 58.6, 53.4, 49.8, 26.9, 21.1 (d, *J* = 1.6 Hz), 14.3.

*4-((4-fluoro-2-methoxyphenyl))-7-((2-methoxypyridin-4-yl)methyl)-5,6,7,8-tetrahydro-1,7-naphthyridine-2-carboxylate* (**13d**). Compound **13d** was prepared in 48% yield as a white solid using a similar method that described for **13a**.^ 1^H NMR (400 MHz, CDCl_3_) δ 8.1 (d, *J* = 5.3 Hz, 1H), 7.8 (s, 1H), 7.1 (dd, *J* = 8.3, 6.6 Hz, 1H), 6.9 (dd, *J* = 5.2, 1.4 Hz, 1H), 6.8 - 6.7 (m, 3H), 4.4 (q, *J* = 7.1 Hz, 2H), 3.9 (s, 3H), 3.9 (s, 2H), 3.8 (s, 3H), 3.7 (s, 2H), 2.7 (s, 4H), 1.4 (t, *J* = 7.1 Hz, 3H).^ 13^C NMR (100 MHz, CDCl_3_) δ 165.4, 164.6, 163.8 (d, J = 247.5 Hz), 157.4 (d, J = 9.9 Hz), 155.2, 149.9, 146.9, 146.8, 145.2, 133.2, 130.9 (d, J = 10.1 Hz), 124.8, 122.7 (d, J = 3.4 Hz), 117.3, 110.7, 107.2 (d, J = 21.4 Hz), 99.3 (d, J = 25.9 Hz), 61.8, 61.3, 58.8, 55.7, 53.4, 50.0, 26.9, 14.4.

*Ethyl 4-(2-fluoro-4-methyphenyl)-7-((2-methoxypyrimidin-5-yl)methyl)-5,6,7,8-tetrahydro-1,7-naphthyridine-2-carboxylate* (**13e**)*.* Compound **13e** was prepared in 45% yield as a white solid using a similar method that described for **13a**. ^1^H NMR (300 MHz, CDCl_3_) δ 8.50 (s, 2H), 7.84 (s, 1H), 7.16 - 6.91 (m, 3H), 4.44 (q, *J* = 7.1 Hz, 2H), 4.01 (s, 3H), 3.88 (s, 2H), 3.66 (s, 2H), 2.40 (s, 3H), 1.39 (t, *J* = 7.1 Hz, 3H). ^13^C NMR (100 MHz, CDCl_3_) δ 164.8, 158.9 (d, *J* = 247.4 Hz), 152.6, 146.2, 145.7, 142.0, 141.9, 132.1, 130.2 (d, *J* = 3.6 Hz), 125.4 (d, *J* = 3.1 Hz), 125.0, 121.8, 121.6, 116.7, 116.5, 62.1, 50.1, 42.7, 36.1, 29.7, 26.8, 21.2, 14.3.

*Ethyl 4-(2-fluoro-4-methoxyphenyl)-7-((2-methoxypyrimidin-5-yl)methyl)-5,6,7,8-tetrahydro-1,7-naphthyridine-2-carboxylate* (**13f**)*.* Compound **13f** was prepared in 42% yield as a white solid using a similar method that described for **13a**. ^1^H NMR (300 MHz, CDCl_3_) δ 8.49 (s, 2H), 7.76 (s, 1H), 7.04 (dd, *J* = 8.2, 6.6 Hz, 1H), 6.79 - 6.64 (m, 2H), 4.43 (q, *J* = 7.2 Hz, 2H), 3.99 (s, 3H), 3.85 (s, 2H), 3.74 (s, 3H), 3.64 (s, 2H), 1.37 (t, *J* = 7.1 Hz, 3H). ^13^C NMR (100 MHz, CDCl_3_) δ 165.3 (d, *J* = 1.9 Hz), 161.5 (d, *J* = 10.9 Hz), 159.9, 159.8 (d, *J* = 247.6 Hz), 155.3, 145.1 (d, *J* = 31.7 Hz), 133.1, 132.1 (d, *J* = 10.1 Hz), 131.0 (d, *J* = 5.3 Hz), 128.5 (d, *J* = 12.2 Hz), 124.8, 124.1, 117.3 (d, *J* = 16.8 Hz), 110.4 (d, *J* = 2.9 Hz), 101.9 (d, *J* = 25.7 Hz), 61.9, 58.6, 56.6, 55.7, 55.0, 49.6, 27.1, 14.3.

*4-(2-chloro-4-fluoropheny)-7-((2-methoxypyrimidin-5-yl)methyl)-5,6,7,8-tetrahydro-1,7-naphthyridine-2-carboxylate* (**13g**)*.* Compound **13g** was prepared in 42% yield as a white solid using a similar method that described for **13a**. ^1^H NMR (400 MHz, CDCl_3_) δ 8.09 (d, *J* = 5.2 Hz, 1H), 7.77 (s, 1H), 7.30 - 7.20 (m, 1H), 7.20 - 7.12 (m, 1H), 7.09 (td, *J* = 8.2, 2.5 Hz, 1H), 6.90 (dd, *J* = 5.3, 1.4 Hz, 1H), 6.76 (s, 1H), 4.45 (qd, *J* = 7.1, 2.9 Hz, 2H), 3.92 (s, 3H), 3.91 - 3.86 (m, 2H), 3.67 (s, 2H), 2.80 - 2.54 (m, 4H), 1.40 (t, *J* = 7.1 Hz, 3H).^ 13^C NMR (100 MHz, CDCl_3_) δ 165.09, 164.58, 162.42 (d, *J* = 251.6 Hz), 155.84, 149.75, 147.11, 146.83, 145.49, 133.33 (d, *J* = 10.3 Hz), 132.82 (d, *J* = 3.8 Hz), 132.65, 131.09 (d, *J* = 8.8 Hz), 124.04, 117.26 (d, *J* = 24.8 Hz), 117.23, 114.52 (d, *J* = 21.3 Hz), 110.63, 61.92, 61.14, 58.72, 53.38, 49.75, 26.98, 14.33.

*4-(2-fluoro-4-methoxyphenyl)-7-((2-methoxypyridin-4-yl)methyl)-5,6,7,8-tetrahydro-1,7-naphthyridine-2-carboxamide* (**14a**)*.* To the solution of compound **13a** (67.7 mg, 0.15 mmol) in MeOH (5 mL) was added ammonia in MeOH (7 *N*, 5 mL). The mixture was stirred for 4 h before the solvent was removed *in vacuo*. The residue was purified by flash chromatography on silica gel (hexanes to ethyl acetate gradient column) to yield compound **14a** as white solid (72%, 45.6 mg). R*_f_* = 0.2 (Hexanes/EtOAc = 1:2). Melting point 151-153 ºC. ^1^H NMR (300 MHz, CDCl_3_) δ 8.08 (d, *J* = 5.2 Hz, 1H), 7.89 (s, 1H), 7.78 (d, *J* = 4.5 Hz, 1H), 7.10 (t, *J* = 8.5 Hz, 1H), 6.90 (d, *J* = 5.2 Hz, 1H), 6.80 - 6.59 (m, 3H), 6.33 (s, 1H), 3.89 (s, 3H), 3.79 (s, 3H), 3.74 (s, 2H), 3.63 (s, 2H), 2.70 (dd, *J* = 14.4, 4.7 Hz, 4H).^ 13^C NMR (75 MHz, CDCl_3_) δ 166.2 , 164.3 , 161.5 (d, *J* = 11.2 Hz), 159.6 (d, *J* = 244.8 Hz), 154.4, 131.9, 131.8 (d, *J* = 5.2 Hz), 121.6, 117.7, 117.3 (d, *J* = 16.4 Hz), 111.3 (d, *J* = 2.8 Hz), 110.3, 102.2 (d, *J* = 25.7 Hz), 60.4, 58.2, 56.2, 53.5, 50.1, 27.0. HRMS (ESI): calculated for C_23_H_24_FN_4_O_3_ [M + H], 423.1832; found, 423.1817.

*4-(2,4-difluorophenyl)-7-((2-methoxypyridin-4-yl)methyl)-5,6,7,8-tetrahydro-1,7-naphthyridine-2- carboxamide* (**14b**). Compound **14b** was prepared in 80% yield as a white solid using a similar method that described for **14a**. Melting point 128-130 ºC. ^1^H NMR (300 MHz, CDCl_3_) δ 8.11 (d, *J* = 5.2 Hz, 1H), 7.90 (s, 1H), 7.78 (s, 1H), 7.36 - 7.15 (m, 1H), 7.14 - 6.85 (m, 3H), 6.78 (s, 1H), 6.01 (d, *J* = 4.6 Hz, 1H), 3.92 (s, 3H), 3.79 (s, 2H), 3.68 (s, 2H), 2.72 (s, 4H). ^13^C NMR (75 MHz, CDCl_3_) δ 166.7, 164.6, 163.2 (dd, *J* = 249.9, 10.0 Hz), 159.3 (dd, *J* = 250.1, 11.9 Hz), 154.2, 145.0, 146.9, 146.6, 144.4, 132.4, 131.5 (dd, *J* = 9.5, 5.0 Hz), 121.8, 121.7 (dd, *J* = 16.8, 4.0 Hz), 117.2, 111.9 (dd, *J* = 21.2, 3.6 Hz), 110.5, 104.4 (t, *J* = 25.5 Hz), 61.1, 58.5, 53.4, 49.9, 26.9 (d, *J* = 3.0 Hz). HRMS (ESI): calculated for C_22_H_21_F_2_N_4_O_2_ [M + H], 411.1633; found, 411.1616

*4-(2-fluoro-4-methyphenyl)-7-((2-methoxypyridin-4-yl)methyl)-5,6,7,8-tetrahydro-1,7-naphthyridine-2-carboxamide* (**14c**). Compound **14c** was prepared in 72% yield as a white solid using a similar method that described for **14a**. ^1^H NMR (300 MHz, CDCl_3_) δ 8.12 (s, 1H), 7.93 (s, 1H), 7.78 (s, 1H), 7.20 - 6.86 (m, 4H), 6.79 (s, 1H), 5.89 (s, 1H), 3.93 (s, 3H), 3.79 (s, 2H), 3.69 (s, 2H), 2.74 (s, 4H), 2.39 (s, 3H). ^13^C NMR (75 MHz, CDCl_3_) δ 166.2 , 164.4 , 158.7 (d, *J* = 244.7 Hz), 154.4, 150.9, 147.9, 147.2, 144.8, 141.7 (d, *J* = 8.1 Hz), 131.7, 130.9 (d, *J* = 3.8 Hz), 126.0 (d, *J* = 2.9 Hz), 122.4 (d, *J* = 16.1 Hz), 121.4, 117.7, 116.6 (d, *J* = 21.7 Hz), 110.3, 60.4, 58.2, 53.5, 50.0, 26.9, 21.1 (d, *J* = 1.6 Hz). HRMS (ESI): calculated for C_23_H_23_FN_4_NaO_3_ [M + H], 407.1883; found, 407.1892.

*4-(4-fluoro-2-methoxyphenyl)-7-((2-methoxypyridin-4-yl)methyl)-5,6,7,8-tetrahydro-1,7-naphthyridine-2- carboxamide* (**14d**)*.* Compound **14d** was prepared in 75% yield as a white solid using a similar method that described for **14a**. ^1^H NMR (300 MHz, CDCl_3_) δ 8.13 (d, *J* = 5.2 Hz, 1H), 7.87 (s, 1H), 7.77 (d, *J* = 4.1 Hz, 1H), 7.05 (dd, *J* = 8.2, 6.7 Hz, 1H), 6.97 (d, *J* = 5.0 Hz, 1H), 6.80 (s, 1H), 6.77 - 6.63 (m, 2H), 5.67 (d, *J* = 4.7 Hz, 1H), 3.94 (s, 3H), 3.81 (s, 2H), 3.75 (s, 3H), 3.72 (s, 2H), 2.74 (s, 4H). ^13^C NMR (75 MHz, CDCl_3_) δ 166.3, 164.3, 162.0, 157.7, 157.6, 152.4 (d, *J* = 223.4 Hz), 147.7, 147.2 (d, *J* = 7.0 Hz), 132.1, 131.4, 131.3, 123.0 (d, *J* = 2.9 Hz), 121.6, 117.8, 110.3, 107.4 (d, *J* = 21.2 Hz), 100.4 (d, *J* = 26.1 Hz), 60.5, 58.2, 56.4, 53.5, 50.1, 26.7. HRMS (ESI): calculated for C_23_H_23_FN_4_NaO_3_ [M + Na], 445.1652; found, 445.1641.

*4-(2-fluoro-4-methyphenyl)-7-((2-methoxypyrimidin-5-yl)methyl)-5,6,7,8-tetrahydro-1,7-naphthyridine-2-carboxamide* (**14e**)*.* Compound **14e** was prepared in 73% yield as a white solid using a similar method that described for **14a**. ^1^H NMR (300 MHz, CDCl_3_) δ 8.53 (s, 2H), 7.93 (s, 1H), 7.77 (s, 1H), 7.15 - 6.93 (m, 3H), 5.56 (s, 1H), 4.02 (s, 3H), 3.78 (s, 2H), 3.67 (s, 2H), 2.73 (s, 4H), 2.40 (s, 3H). ^13^C NMR (100 MHz, CDCl_3_) δ 166.4, 158.8 (d, *J* = 247.4 Hz), 151.0, 147.3, 146.2, 141.9 (d, *J* = 8.0 Hz), 131.5, 130.3 (d, *J* = 3.7 Hz), 125.4 (d, *J* = 3.0 Hz), 122.5, 121.8 (d, *J* = 16.3 Hz), 116.5 (d, *J* = 21.6 Hz), 49.7, 42.8, 36.2, 26.6, 26.6 , 21.2. HRMS (ESI): calculated for C_22_H_23_FN_5_O_2_ [M + H], 408.1836; found, 408.1826.

*4-(2-fluoro-4-methoxyphenyl)-7-((2-methoxypyrimidin-5-yl)methyl)-5,6,7,8-tetrahydro-1,7-naphthyridine-2-carboxamide* (**14f**)*.* Compound **14f** was prepared in 80% yield as a white solid using a similar method that described for **14a**. ^1^H NMR (300 MHz, CDCl_3_) δ 8.54 (s, 2H), 7.93 (s, 1H), 7.78 (s, 1H), 7.13 (t, *J* = 8.6 Hz, 1H), 6.91 - 6.61 (m, 2H), 5.70 (s, 1H), 4.02 (s, 3H), 3.85 (s, 3H), 3.78 (s, 2H), 3.67 (s, 2H), 2.74 (s, 4H). ^13^C NMR (75 MHz, *d*_6_-DMSO) δ 166.2, 165.1, 161.5 (d, *J* = 11.2 Hz), 160.4, 159.6 (d, *J* = 244.8 Hz), 154.3, 147.9, 144.7, 131.9, 131.7 (d, *J* = 5.0 Hz), 124.9, 121.6, 117.3 (d, *J* = 16.4 Hz), 111.3, 102.2 (d, *J* = 25.7 Hz), 57.9, 56.2, 55.7, 55.0, 49.7, 26.9. HRMS (ESI): calculated for C_22_H_23_FN_5_O_3_ [M + H], 424.1785; found, 424.1785.

*4-(2-chloro-4-fluorophenyl)-7-((2-methoxypyrimidin-5-yl)methyl)-5,6,7,8-tetrahydro-1,7-naphthyridine-2-carboxamide* (**14g**)*.* Compound **14g** was prepared in 70% yield as a white solid using a similar method that described for **14a**. ^1^H NMR (300 MHz, *d*_6_-DMSO) δ 8.12 (d, *J* = 5.2 Hz, 1H), 8.00 (s, 1H), 7.71 - 7.56 (m, 3H), 7.54 - 7.41 (m, 1H), 7.36 (td, *J* = 8.5, 2.5 Hz, 1H), 7.00 (d, *J* = 4.8 Hz, 1H), 6.80 (s, 1H), 3.84 (s, 3H), 3.71 (s, 4H), 2.78 - 2.63 (m, 2H), 2.65 - 2.50 (m, 2H). ^13^C NMR (75 MHz, *d*_6_-DMSO) δ 166.1, 164.3, 154.5, 150.9, 148.0, 147.2, 133.3 (d, *J* = 3.5 Hz), 132.8 (d, *J* = 11.6 Hz), 132.6, 132.3 (d, *J* = 9.1 Hz), 131.6, 121.0, 117.7, 117.4 (d, *J* = 24.7 Hz), 115.5, 115.2, 110.3, 60.4, 58.1, 53.5, 49.9, 26.7. HRMS (ESI): calculated for C_22_H_21_FN_4_O_2_ [M + H], 427.1337; found, 427.1325.

### Chemical syntheses of radiolabeling precursors 17 and 18

*Ethyl 4-chloro-7-((2-methoxypyridin-4-yl)methyl)-5,6,7,8-tetrahydro-1,7-naphthyridine-2-carboxylate* (**16**)*.* Compound **16** was prepared in 45% yield as a white solid using a similar method that described for **13a**. ^1^H NMR (300 MHz, CDCl_3_) δ 8.10 (d, *J* = 5.2 Hz, 1H), 7.97 (s, 1H), 6.90 (d, *J* = 4.7 Hz, 1H), 6.84 - 6.62 (m, 1H), 4.44 (q, *J* = 7.1 Hz, 2H), 3.93 (s, 3H), 3.81 (s, 2H), 3.69 (s, 2H), 2.97 (t, *J* = 5.9 Hz, 2H), 2.82 (t, *J* = 5.9 Hz, 2H), 1.40 (t, *J* = 7.1 Hz, 3H). ^13^C NMR (75 MHz, CDCl_3_) δ 164.6, 164.2, 156.8, 149.4, 146.9, 146.2, 144.9, 132.4, 123.8, 117.1, 110.7, 62.1, 60.9, 58.2, 53.4, 49.6, 26.8, 14.2.

*4-(2-fluoro-4-hydroxyphenyl)-7-((2-methoxypyridin-4-yl)methyl)-5,6,7,8-tetrahydro-1,7-naphthyridine-2-carboxamide* (**17**). To a solution of ethyl 4-chloro-7-((2-methoxypyrimidin-5-yl)methyl)-5,6,7,8-tetrahydro-1,7-naphthyridine-2-carboxylate (**16**) (10.8 mg, 0.030 mmol), 2-fluoro-4-methoxyphenyl boronic acid (5.1 mg, 0.030 mmol) and K_2_CO_3_ (8.28 mg, 0.060 mmol) in 1,4-dioxane/water (v/v, 10/1, 1.8 mL) was added Pd(dppf)Cl_2_ (2.2 mg, 0.003 mmol) under Ar. The mixture was stirred at 100 ^o^C for 4 h, then quenched with water (3 mL) and extracted with ethyl acetate (5 mL, three times). The combined organic layers were concentrated *in vacuo*. The residue was dissolved in 7 N ammonia methanol solution (2 mL) and stirred for 4h before quenched with ethyl acetate (5 mL) and concentrated *in vacuo.* The residue was purified by flash chromatography on silica gel (hexanes to ethyl acetate gradient column) to yield the compound **17** as white solid (5.3 mg, 44%). ^1^H NMR (300 MHz, CDCl_3_) δ 8.13 (d, *J* = 5.3 Hz, 1H), 7.87 (s, 2H), 7.08 - 6.93 (m, 2H), 6.84 - 6.65 (m, 3H), 5.68 (s, 1H), 3.95 (s, 3H), 3.81 (s, 2H), 3.71 (s, 2H), 2.77 (s, 4H).^13^C NMR (75 MHz, CDCl_3_) δ 165.2 , 164.6 , 159.7 (d, *J* = 247.2 Hz), 159.1 , 159.0 , 146.6 , 145.5 , 145.0 , 133.3 , 131.0 (d, *J* = 5.2 Hz), 124.9 , 117.4 , 116.3 (d, *J* = 16.4 Hz), 112.1 , 112.0 , 110.6 , 103.6 (d, *J* = 24.7 Hz), 61.0 , 58.4 , 53.6 , 49.7 , 27.0. HRMS (ESI): calculated for C_22_H_22_FN_4_O_3_ [M + H], 409.1676; found, 409.1691.

*4-(2,4-difluorophenyl)-7-((2-hydroxypyridin-4-yl)methyl)-5,6,7,8-tetrahydro-1,7-naphthyridine-2-carboxamide* (**18**) Precursor **18** was prepared in 56% yield as a white solid using a similar method that described for **14a**. ^1^H NMR (400 MHz, CDCl_3_) δ 8.36 (d, *J* = 5.0 Hz, 1H), 7.94 (s, 1H), 7.80 (d, *J* = 4.6 Hz, 1H), 7.42 (d, *J* = 1.3 Hz, 1H), 7.32 - 7.28 (m, 1H), 7.24 (td, *J* = 8.4, 6.3 Hz, 1H), 7.04 - 6.89 (m, 2H), 6.13 (d, *J* = 4.6 Hz, 1H), 3.81 (s, 2H), 3.75 (s, 2H), 2.76 (s, 4H). ^13^C NMR (100 MHz, CDCl_3_) δ 166.7, 163.2 (dd, *J* = 250.8, 11.4 Hz), 159.2 (dd, *J* = 250.4, 11.8 Hz), 153.8, 151.9, 150.9, 149.8, 146.8, 144.4, 132.5, 131.5 (dd, *J* = 9.6, 4.9 Hz), 124.0, 122.4, 121.9, 121.6 (dd, *J* = 16.7, 3.8 Hz), 111.9 (dd, *J* = 21.1, 3.7 Hz), 104.4 (t, *J* = 25.5 Hz), 60.7, 58.5, 50.0, 26.9 (d, *J* = 3.5 Hz). HRMS (ESI): calculated for C_22_H_19_F_2_N_4_O_2_ [M + H], 397.1476; found, 397.1490.

### Pharmacology

*Cell Line Generation and Thallium Flux Assays*. The general procedure for the preparation of human mGlu_2_ and mGlu_3_ was described previously 45, 56 with minor modifications in this work. The cloning sites were NheI/NotI for both receptors. HEK GIRK cells, generously provided by Lily Jan (University of California San Francisco, San Francisco, CA), were transfected with 24 μg of DNA using Fugene6 (Promega), stable transfectants were selected with 1 μg/mL puromycin dihydrochloride (Sigma-Aldrich, St. Louis, MO), and polyclonal human mGlu_2_ GIRK and mGlu_3_ GIRK cell lines were established. Cells were maintained following selection in 45% DMEM, 45% Ham's F12, 10% FBS, 100 units/mL penicillin/streptomycin, 20 mM HEPES, pH 7.3, 1 mM sodium pyruvate, 2 mM glutamine, 700 μg/mL G418 (Mediatech, Inc., Herndon, VA), and 600 μg/mL puromycin (growth media) at 37 °C in the presence of 5% CO_2_. All cell culture reagents were purchased from Invitrogen Corp. (Carlsbad, CA) unless otherwise noted.

*Human mGlu_2_ and mGlu_3_ Thallium Flux in Vitro Assays*. Potencies of these NAMs at both mGlu_2_ and mGlu_3_ were investigated by thallium flux through GIRK channels, which was disclosed in detail 57 as well as described 45 in our previous work. In particular, cells were plated into 384-well, black-walled, clear-bottomed poly(D-lysine_-coated plates at a density of 15 000 cells per well in 20 μL of DMEM containing 10% dialyzed FBS, 20 mM HEPES, and 100 units/mL penicillin/streptomycin (assay media). Plated cells were incubated overnight at 37 °C in the presence of 5% CO_2_. The next day, the medium was exchanged from the cells to assay buffer [Hanks' balanced salt solution (Invitrogen) containing 20 mM HEPES, pH 7.3] using an ELX405 microplate washer (BioTek), leaving 20 μL per well, followed by the addition of 20 μL per well of FluoZin2-AM (330 nM final concentration) indicator dye (Invitrogen; prepared as a stock in DMSO and mixed in a 1:1 ratio with Pluronic acid F-127) in assay buffer. Cells were incubated for 1 h at room temperature, and the dye was exchanged to assay buffer using an ELX405, leaving 20 μL per well. For concentration-response curve experiments, compounds were serially diluted 1:3 into 10 point concentration-response curves and were transferred to daughter plates using an Echo acoustic plate reformatter (Labcyte, Sunnyvale, CA). Test compounds were diluted to 2 times their final desired concentration in assay buffer (0.3% DMSO final concentration). Agonists were diluted in thallium buffer [125 mM sodium bicarbonate (added fresh the morning of the experiment), 1 mM magnesium sulfate, 1.8 mM calcium sulfate, 5 mM glucose, 12 mM thallium sulfate, and 10 mM HEPES, pH 7.3] at 5 times the final concentration to be assayed. Cell plates and compound plates were loaded onto a kinetic imaging plate reader (FDSS 6000 or 7000; Hamamatsu Corporation, Bridgewater, NJ). Appropriate baseline readings were taken (10 images at 1 Hz; excitation, 470 ± 20 nm; emission, 540 ± 30 nm), and test compounds were added in a 20 μL volume and incubated for approximately 1 h at room temperature before the addition of 10 μL of thallium buffer with or without an EC_80_ concentration of the agonist glutamate for affinity evaluation experiments or with a full concentration-response of glutamate for Schild analysis experiments. After the addition of agonist, data were collected for approximately an additional 2.5 min. Data were analyzed using Excel (Microsoft Corp, Redmond, WA). The slope of the fluorescence increase beginning 5 s after thallium/agonist addition and ending 15 s after thallium/agonist addition was calculated, corrected to vehicle and maximal agonist control slope values, and plotted using either XLfit (ID Business Solutions Ltd.) or Prism software (GraphPad Software, San Diego, CA) to generate concentration-response curves. Potencies were calculated from fits using a four-point parameter logistic equation.

### Radiochemistry

*Radiosynthesis of 4-(2-fluoro-4-(methoxy-^11^C)phenyl)-7-((2-methoxy-pyridin-4-yl)methyl)-5,6,7,8-tetrahydro-1,7-naphthyridine-2-carboxamide* ([^11^C]**14a**). [^11^C]Methyl iodide ([^11^C]CH_3_I) was synthesized from cyclotron-produced [^11^C]CO_2_, which was produced by ^14^N(p, α)^11^C nuclear reaction. Briefly, [^11^C]CO_2_ was bubbled into a solution of LiAlH_4_ (0.4 M in THF, 300 μL). After evaporation, the remaining reaction mixture was treated with hydroiodic acid (57% aqueous solution, 300 μL). The resulting [^11^C]CH_3_I was transferred under helium gas with heating into a pre-cooled (-15 to -20 °C) reaction vessel containing precursor **17** (1.0 mg), NaOH (3-5 μL, 0.5 M) and anhydrous DMF (300 μL). After the radioactivity reached a plateau during transfer, the reaction vessel was warmed to 80 °C and maintained for 5 min. HPLC purification was completed on a Capcell Pak UG80 C18 column (10 mm ID × 250 mm) using a mobile phase of CH_3_CN / H_2_O (v/v, 55/45) at a flow rate of 5.0 mL/min. The retention time of [^11^C]**14a** was 9.0 min. The radioactive fraction corresponding to the desired product was collected in a sterile flask, evaporated to dryness *in vacuo*, and reformulated in a saline solution (3 mL) containing 100 µL of 25% ascorbic acid in sterile water and 100 µL of 20% Tween^®^ 80 in ethanol. (Note: We added ascorbic acid to prevent potential radiolysis and Tween^®^ 80 to improve aqueous solubility.) The synthesis time was *ca.* 40 min from end-of-bombardment. Radiochemical and chemical purity was measured by analytical HPLC (Capcell Pak UG80 C18, 4.6 mm ID × 250 mm). The identity of [^11^C]**14a** was confirmed by the co-injection with unlabeled **14a**. Radiochemical yield was 36.6 ± 7.3% (*n* = 7, decay-corrected based on [^11^C]CO_2_) with >99% radiochemical purity and greater than 2 Ci/μmol molar activity.

*Radiosynthesis of 4-(2,4-difluorophenyl)-7-((2-(methoxy-^11^C)pyridine-4-yl)methyl)-5,6,7,8-tetrahydro-1,7-naphthyridine-2-carboxamide* ([^11^C]**14b**). Similar radiosynthesis of [^11^C]**14a**, [^11^C]CH_3_I was trapped in the reaction vessel containing precursor **18** (1.0 mg) and anhydrous DMF (300 μL, pre-saturated with 10-20 mg Cs_2_CO_3_). After the radioactivity reached a plateau during transfer, the reaction vessel was warmed to 90 °C and maintained for 5 min. HPLC purification was completed on a Capcell Pak UG80 C18 column (10 mm ID × 250 mm) using a mobile phase of CH_3_CN / H_2_O + 0.1% Et_3_N (v/v, 55/45) at a flow rate of 5.0 mL/min. The retention time of [^11^C]**14b** was 9.0 min. The radioactive fraction corresponding to the desired product was collected in a sterile flask, evaporated to dryness in vacuo, and reformulated in a saline solution (3 mL) containing 100 µL of 25% ascorbic acid in sterile water and 100 µL of 20% Tween^®^ 80 in ethanol. The synthesis time was ca. 40 min from end-of-bombardment. Radiochemical and chemical purity was measured by analytical HPLC (Capcell Pak UG80 C18, 4.6 mm ID × 250 mm). The identity of [^11^C]**14b** was confirmed by the co-injection with unlabeled **14b**. Radiochemical yield was 6.53 ± 1.5% (n = 10, decay-corrected based on [^11^C]CO_2_) with >99% radiochemical purity and greater than 2 Ci/μmol molar activity.

### *In Vitro* Autoradiography

Sagittal rat brain slices were prepared into 20 μm sections with a cryostat (HM560; Carl Zeiss, Oberkochen, Germany), mounted on air plasma spray-coated glass slides, and stored at -80 °C before used for experiments. The sections were preincubated for 15 min in a glass tank containing 200 mL of Tris·HCl buffer (50 mM, pH 7.4) consisting of 2 mM of MgCl_2_ and 1.2 mM of CaCl_2_ at room temperature. After preincubation, the sections were incubated in a fresh buffer containing [^11^C]**14a** or [^11^C]**14b** (250 µCi in 200 mL buffer; molar activity 2.7 Ci/µmol of [^11^C]**14a** and 2.5 Ci/µmol of [^11^C]**14b**, respectively) in the glass tank for 30 min at room temperature. For blocking studies, non-radioactive **14a** or **14b** (10 μM) and **QCA** (10 μM) were chosen to determine the specificity of radiotracers for binding mGlu_2_. Six serial brain sections were used for each conditions. After incubation, brain sections were washed with cold buffer (3 × 5 min), immersed in cold distilled water, and then dried with cold air. The sections were placed in contact with imaging plates (BAS-MS2025, FUJIFILM, Tokyo, Japan). Autoradiograms were obtained and photostimulated luminescence (PSL) values in the ROIs were measured using a Bio-Imaging Analyzer System (BAS5000, FUJIFILM).

### Small Animal PET Imaging Studies in Rat Brain

As we previously reported 45, PET scans were carried out by an Inveon PET scanner (Siemens Medical Solutions, Knoxville, TN, USA). During the scan, SD rats were anesthetized by oxygen mixed with 1-2% (v/v) isoflurane and kept body temperature at 40 °C by a commercially available circulation system (T/Pump TP401, Gaymar Industries, Orchard Park, NY). A 24-gauge catheter was inserted into the tail vein to facilitate a bolus injection. The radiotracer [^11^C]**14a** or [^11^C]**14b** (*ca.* 1 mCi / 150-200 μL) was injected into the rat via the preinstalled catheter, and the dynamic acquisition of PET signals in rat brain was started at the same time and lasted for 60 min in 3D list mode. For pretreatment studies, **14a** (1 mg/kg for [^11^C]**14a** for self-blocking study in **Figure S2**; 3 mg/kg for [^11^C]**14b** for blocking study in **Figure S12C**) or **14b** (1 mg/kg for [^11^C]**14b** for self-blocking study in **Figure S12B**), formulated in 300 μL of saline containing 10% ethanol and 5% Tween 80, was injected at 30 min via the tail vein catheter prior to the injection of [^11^C]**14a** or [^11^C]**14b**. The dynamic emission data were reconstructed by filtered back projection using Hanning's filter with a Nyquist cutoff of 0.5 cycle/pixel into 33 frames (10 s × twelve frames, 20 s × three frames, 30 s × three frames, 60 s × three frames, 150 s × three frames, and 300 s × nine frames). The TACs of [^11^C]**14a** or [^11^C]**14b** were analyzed from volumes of interest in the striatum, hippocampus, cortex, thalamus, pons and cerebellum normalized to a rat brain MRI template 58 using PMOD software (version 3.4; PMOD technology, Zurich, Switzerland). The radioactivity was decay-corrected to the injection time and expressed as the standardized uptake value (SUV) which equals to (radioactivity per milliliter of tissue per injected radioactivity) × (gram of body weight). To obtain non-displaceable binding potential (BP_ND_), simplified reference tissue model (SRTM) 53, 54 was carried out on the basis of kinetic analysis using PMOD software. We acquired BP-parametric images using kinetic modeling without masking. Respective TACs in receptor-rich or -poor region were loaded for production of BP-map with default setting. Representative parametric images scaled with BP_ND_ were reconstructed by PMOD software using TACs in mGlu_2_-enriched and reference regions. Pons was selected as the reference region for mGlu_2_ (the lowest and consistent brain uptake and washout kinetics between baseline and blocking conditions). It should be noted that while there is a low-level expression of mGlu2 in the pons, it is not without existence. A reference region should be a region devoid of the target, but with similar tracer transport or diffusion to the other regions of interest with expression of the target. Therefore, there are some limitations when using pons as the reference region. For example, the partial volume effect in PET likely leads to spillover of activity from nearby higher uptake regions into the pons, artificially increasing the time-activity curve in the pons.

### Whole Body *ex vivo* Biodistribution Studies in Mice

Each mouse was treated with a bolus injection of [^11^C]**14b** (50 μCi / 150 μL) via the tail vein. Three mice were sacrificed by cervical dislocation at each time point (1, 5, 15, 30 and 60 min) after injection. Major organs, including heart, lungs, liver, pancreas, spleen, kidneys, stomach (including contents), small intestine (including contents), large intestine (including contents), testes, muscle, whole brain and blood samples were quickly removed and weighed. The radioactivity remained in these organs was measured by a 2480 Wizard autogamma counter (PerkinElmer, USA). The results are expressed as the percentage of injected dose per gram of wet tissue (%ID/g) or standardized uptake value (SUV). All radioactivity measurements were decay-corrected to the time point of PET tracer injection based on half-life of ^11^C.

### Radiometabolite Analysis

Sprague-Dawley rats were sacrificed by decapitation under anesthesia at 5 and 20 min (*n* = 2 each time point) after the intravenous injection of [^11^C]**14b**. Blood and whole brain samples were quickly harvested. Plasma was separated from the blood samples via 1) centrifuging at 15,000 g for 2 min at 4 °C, 2) mixing 0.5 mL of acetonitrile with 0.5 mL of supernatant, and then 3) vortexing for 15 s and centrifuging again at 15,000 g for 2 min for deproteinization. In terms of brain metabolite analysis, the removed rat brain was quickly placed on the ice, homogenized in an ice-cooled CH_3_CN/H_2_O (v/v, 1/1, 1 mL) solution, and then centrifuged at 15,000 g for 2 min at 4 °C. The supernatant was collected, whose radioactivity was >90% based on the total radioactivity in the brain homogenate. An aliquot of the supernatant (100 μL) obtained from the plasma or brain homogenate was injected into the radio-HPLC system and analyzed using a Capcell Pak UG80 C18 column (4.6 mm ID × 250 mm) in a mobile phase of CH_3_CN/H_2_O + 0.1% Et_3_N (v/v, 45/55) at a flow rate of 1.0 mL/min. The percentage of [^11^C]**14b** to total radioactivity (corrected for decay) on the HPLC charts was calculated as ([peak area for [^11^C]**14b**]/[total peak area]) × 100.

### Statistical analysis

Statistical analysis is performed using the statistical computer package, GraphPad Prism (GraphPad Software Inc., San Diego, CA, USA). Results are expressed as means ± SEM. Statistical comparisons were made using one-way analysis of variance (ANOVA). Asterisks indicate statistical significance. *p < 0.05, ***p ≤ 0.001, and ****p ≤ 0.0001.

## Supplementary Material

Supplementary figures and tables.

## Figures and Tables

**Table 1:** Pharmacology and physiochemical properties of mGlu_2_NAMs **14a-14g**

	IC_50_ (nM) for mGlu_2_*^a^*				
compd.	mean	SEM	IC_50_ (μM) for mGlu_3_*^b^*	cLog*D^c^*	tPSA*^c^*
14a	39	10	> 10	3.48	89.51
14b	24	5	> 10	3.54	80.28
14c	129	24	> 10	3.90	80.28
14d	39	6	> 10	2.92	89.51
14e	106	14	> 10	3.07	92.62
14f	318	26	> 10	2.66	101.87
14g	87*^d^*	/	> 30*^e^*	3.87	80.28

*^a^*Values of *in vitro* affinity were measured in triplicate assays in mGlu_2_ GIRK or *^b^*mGlu_3_ GIRK. *^c^*Values were calculated with ChemDraw 16.0 software.*^ d^*Values were tested in duplicate assays in mGlu_2_ GIRK or *^e^*mGlu_3_ GIRK.

## Abbreviations

mGlu_2_: metabotropic glutamate receptor 2
PD: Parkinson's disease
AD: Alzheimer's disease
PET: positron emission tomography
CNS: central nervous system
iGluRs: ionotropic glutamate receptors
mGlus: metabotropic glutamate receptors
PAMs: positive allosteric modulators
NAMs: negative allosteric modulators
BBB: blood-brain barrier
PgP: P-glycoprotein
Bcrp: breast cancer resistance protein
ARG: autoradiography
ABC: ATP binding cassette
NBS: N-bromosuccinimide
DMF: dimethylformamide
GTP: Guanosine triphosphate
cAMP: cyclic adenosine monophosphate
tPSA: topological polar surface area
GIRK: G protein-coupled inwardly rectifying potassium
BPND: non-displaceable binding potential
SUV: standardized uptake value
TAC: time-activity curve
%ID/g: the percentage of injected dose per gram of wet tissue
