# Supplementary material for: Synthesis and preliminary studies of 11C-labeled tetrahydro-1,7-naphthyridine-2-carboxamides for PET imaging of metabotropic glutamate receptor 2
Source: Theranostics. 2020 Sep 14;10(24):11178–96. doi: 10.7150/thno.42587 (PMC7532674; doi:10.7150/thno.42587)

Supporting Information

Synthesis and preliminary studies of ^{11}C-labeled tetrahydro-1,7-naphthyridine-2-carboxamides for PET imaging of metabotropic glutamate receptor 2

Xiaofei Zhang,[1,2,†] Yiding Zhang,[3,†] Zhen Chen,[1] Tuo Shao,[1] Richard Van,[4] Katsushi Kumata,[3] Xiaoyun Deng,[1] Hualong Fu,[1] Tomoteru Yamasaki,[3] Jian Rong,[1] Kuan Hu,[3] Akiko Hatori,[3] Lin Xie,[3] Qingzhen Yu,[1] Weijian Ye,[5] Hao Xu,[5] Douglas J. Sheffler,[6] Nicholas D. P. Cosford,[6] Yihan Shao,[4] Pingping Tang,[2] Lu Wang,[1,5,*] Ming-Rong Zhang,[3,*] Steven H. Liang[1,*]

[1]Division of Nuclear Medicine and Molecular Imaging, Massachusetts General Hospital & Department of Radiology, Harvard Medical School, Boston, MA, 02114, USA

[2]State Key Laboratory and Institute of Elemento-Organic Chemistry, Collaborative Innovation Center of Chemical Science and Engineering, Nankai University, Tianjin 300071, China

[3]Department of Radiopharmaceuticals Development, National Institute of Radiological Sciences, National Institutes for Quantum and Radiological Science and Technology, 4-9-1 Anagawa, Inage-ku, Chiba 263-8555, Japan

[4]Department of Chemistry and Biochemistry, University of Oklahoma, Norman, Oklahoma 73019, United States

[5]Department of Nuclear Medicine and PET/CT-MRI Center, The First Affiliated Hospital of Jinan University, 613 West Huangpu Road, Tianhe District, Guangzhou 510630, China

[6]Cancer Metabolism and Signaling Networks Program and Conrad Prebys Center for Chemical Genomics, Sanford-Burnham Prebys Medical Discovery Institute, La Jolla, California 92037, United States

[*]Corresponding authors.

liang.steven@mgh.harvard.edu (S. H. Liang); zhang.ming-rong@qst.go.jp (M.-R. Zhang); l_wang1009@foxmail.com (L. Wang)

[†]These two authors contributed equally to this work.

Contents

Figure S1. Concentration-response curves of $mGlu_2$ NAMs compounds **14** in $mGlu_2$ GIRK or $mGlu_3$ GIRK functional assays..1

Figure S2. Time-activity curves of [^{11}C]**14a** in rat brains...2

Figure S3. Radiometabolite analysis of [^{11}C]**14a** in rat brain and plasma...3

Table S1. Radiometabolite and parent (unchanged) fraction of [^{11}C]**14a** in rat brain and plasma 3

Figure S4. RadioHPLC chromatogram in the brain and plasma 5 min post injection of [^{11}C]**14a**......4

Figure S5. RadioHPLC chromatogram in the brain and plasma 20 min post injection of [^{11}C]**14a**..4

Figure S6. Radiometabolite analysis of [^{11}C]**14b** in rat brain and plasma…………..………………....5

Table S2. Radiometabolite and parent (unchanged) fraction of [^{11}C]**14b** in rat brain and plasma ….5

Figure S7. RadioHPLC chromatogram in the brain and plasma 5 min post injection of [^{11}C]**14b**….5

Figure S8. RadioHPLC chromatogram in the brain and plasma 20 min post injection of [^{11}C]**14b**....6

Figure S9. Image of the ROIs used for quantification of in vitro autoradiography...........................7

Figure S10. Functional (agonist and antagonist) assays of compound **14b** towards mGlu receptors...7

Figure S11. Off-target pharmacological evaluation of compound **14b** at a concentration of 10 μM against major CNS targets...8

Figure S12. Representative PET/MRI fused images……………………………………….9

NMR spectra of synthesized compounds..10

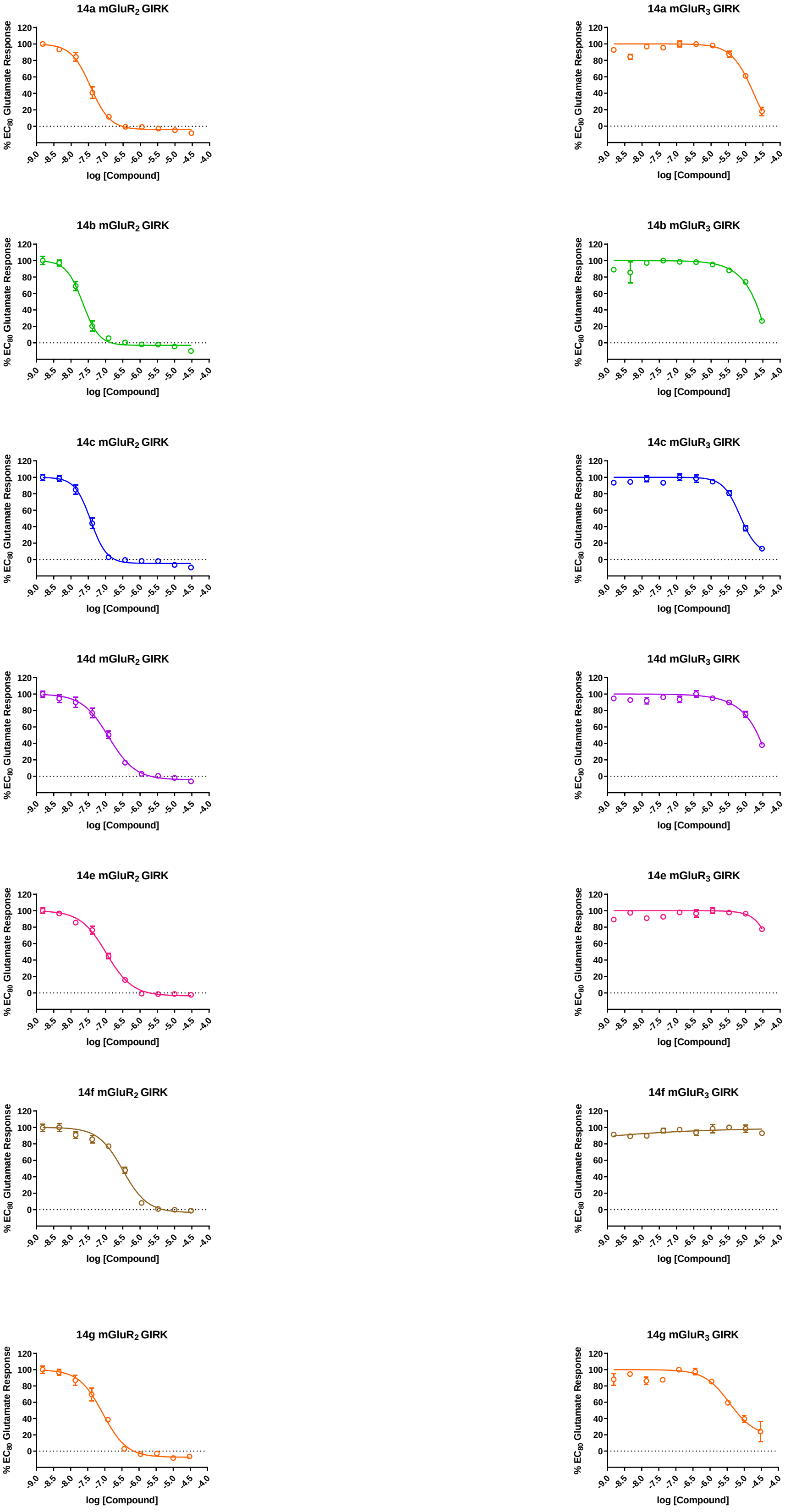

Figure S1. Concentration−response curves of mGlu2 NAMs compounds **14a-14g** in mGlu2 GIRK or mGlu3 GIRK functional assays.

A. Baseline studies of [^{11}C]14a

B. Baseline studies with pretreatment of 14a

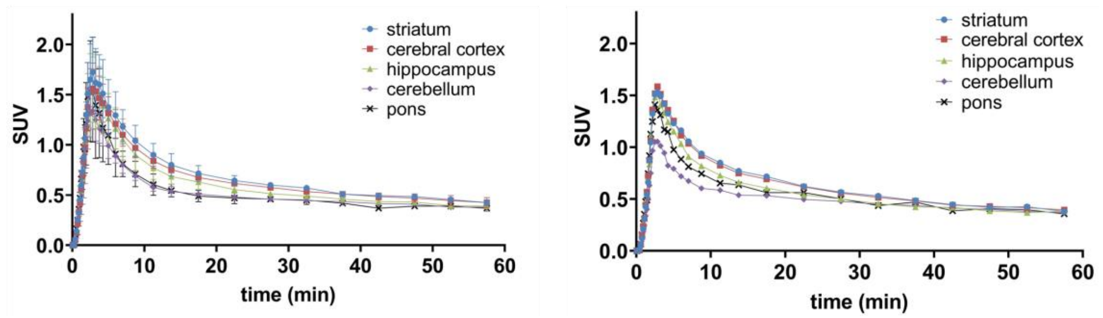

Figure S2. Time-activity curves of [^{11}C]**14a** in rat brains. [a]Blocking conditions: **14a** (1 mg/kg), 30 min *i.v.* before radioligand injection.

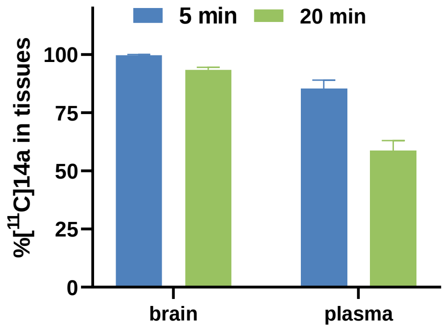

Figure S3. Radiometabolite analysis of [^{11}C]**14a** in rat brain (average two runs)

Table S1. Radiometabolite and parent (unchanged) fraction of [^{11}C]**14a** in rat brain and plasma

	Metabolite (%)	Unchanged (%)
Plasma 5 mins−1	11.96	88.04
Plasma 5 mins−2	17.06	82.94
Plasma 20 mins−1	44.51	55.49
Plasma 20 mins−2	38.26	61.74
Brain 5 mins−1	0.06	99.94
Brain 5 mins−2	0.49	99.51
Brain 20 mins−1	7.3	92.7
Brain 20 mins−2	5.76	94.24

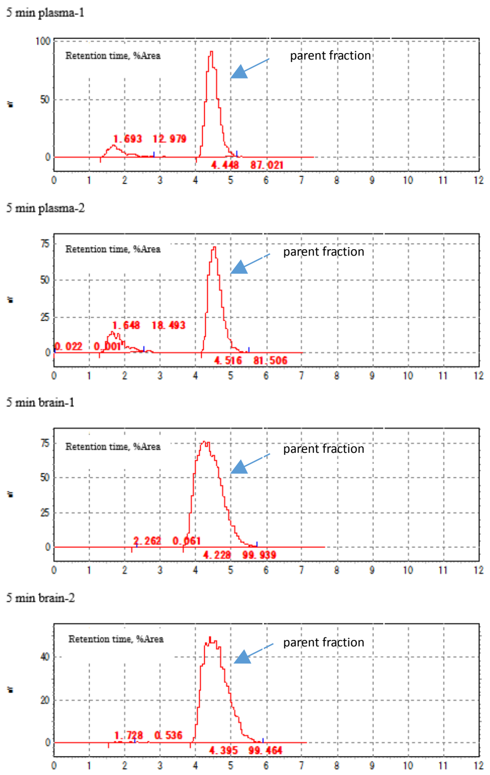

Figure S4. RadioHPLC chromatogram in the brain and plasma 5 min post injection of [^{11}C]**14a**

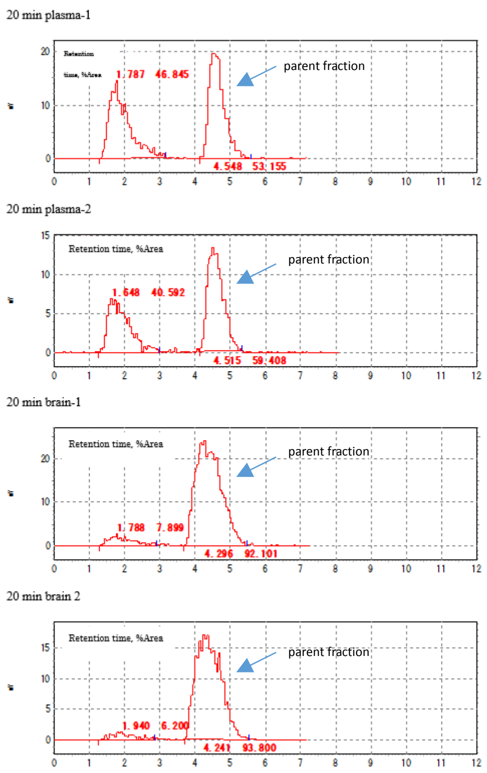

Figure S5. RadioHPLC chromatogram in the brain and plasma 20 min post injection of [^{11}C]**14a**

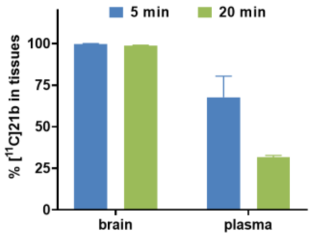

Figure S6. Radiometabolite analysis of [^{11}C]**14b** in rat brain (average two runs)

Table S2. Radiometabolite and parent (unchanged) fraction of [^{11}C]**14b** in rat brain and plasma

	Metabolite(%)	Unchanged (%)
Plasma 5 mins−1	21.32	76.68
Plasma 5 mins−2	41.31	58.69
Plasma 20 mins−1	69.22	30.78
Plasma 20 mins−2	67.60	32.40
Brain 5 mins−1	0.28	99.72
Brain 5 mins−2	0.04	99.96
Brain 20 mins−1	0.97	99.03
Brain 20 mins−2	1.22	98.78

5 min plasma-1

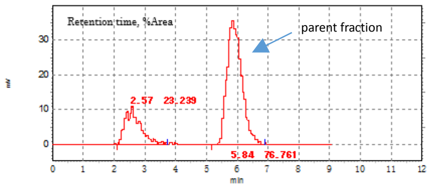

5 min plasma-2

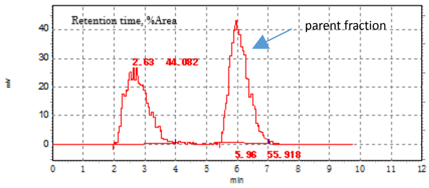

5 min brain-1

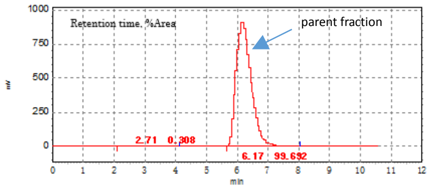

5 min brain-2

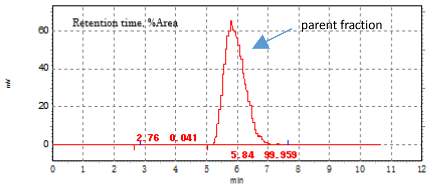

Figure S7. RadioHPLC chromatogram in the brain and plasma 5 min post injection of [^{11}C]**14b**

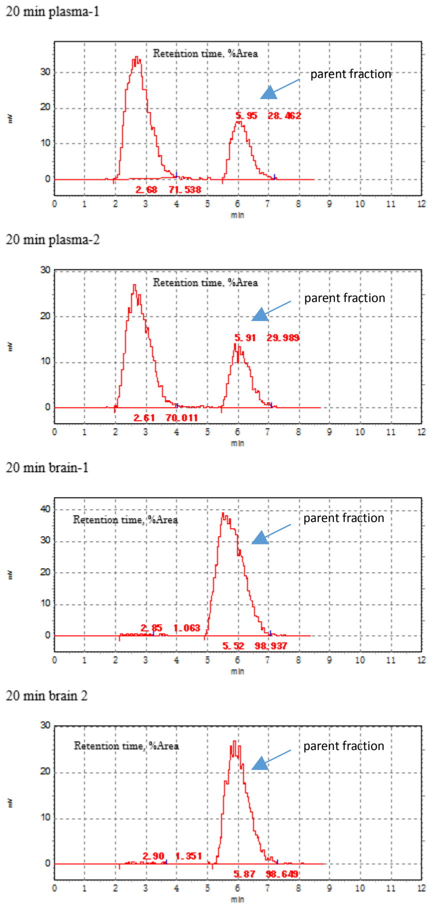

Figure S8. RadioHPLC chromatogram in the brain and plasma 20 min post injection of [^{11}C]**14b**

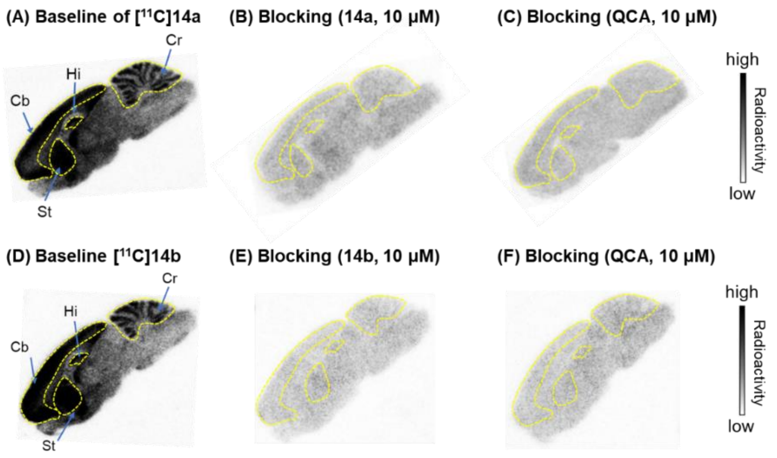

Figure S9. Image of the ROIs used for quantification of in vitro autoradiography. (A) Brain sections were treated with [^{11}C]**14a**; (B) Brain sections were pre-treated with **14a** (10 μM), followed by [^{11}C]**14a**; (C) Brain sections were pre-treated with **QCA** (10 μM), followed by [^{11}C]**14a**; (A) Brain sections were treated with [^{11}C]**14b**; (B) Brain sections were pre-treated with **14b** (10 μM), followed by [^{11}C]**14b**; (C) Brain sections were pre-treated with **QCA** (10 μM), followed by [^{11}C]**14b**.

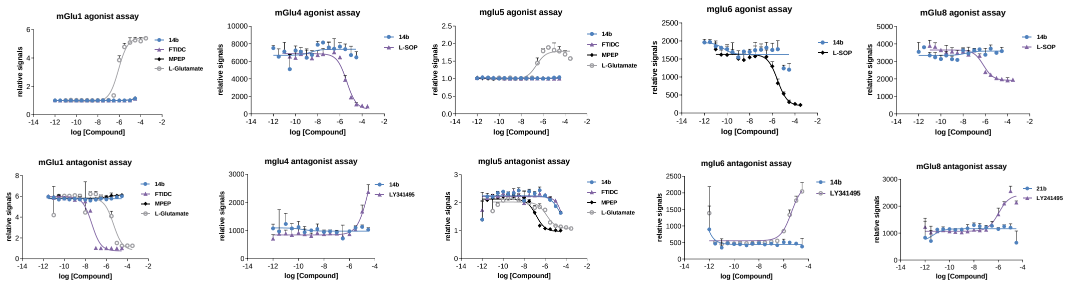

Figure S10. Functional (agonist and antagonist) assays of compound **14b** towards mGlu receptors, including mGlu$_1$, mGlu$_4$, mGlu$_5$, mGlu$_6$ and mGlu$_8$.

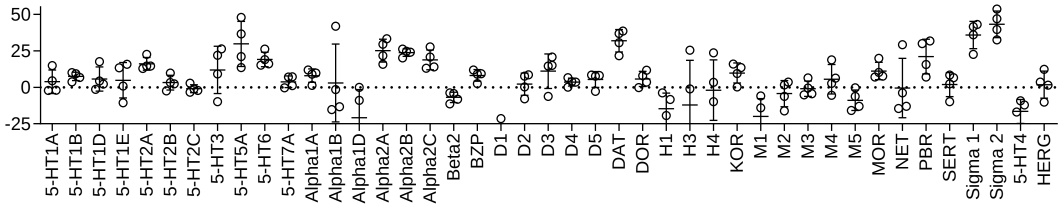

Figure S11. Off-target pharmacological evaluation of compound **14b** at a concentration of 10 µM against major CNS targets, including common GPCRs, enzymes, ion channels and transporters: Initial screening at a concentration of 10 µM. All data are mean ± SD (n = 4). No significant off-target binding (>50%) was observed at 10 µM compound testing concentration.

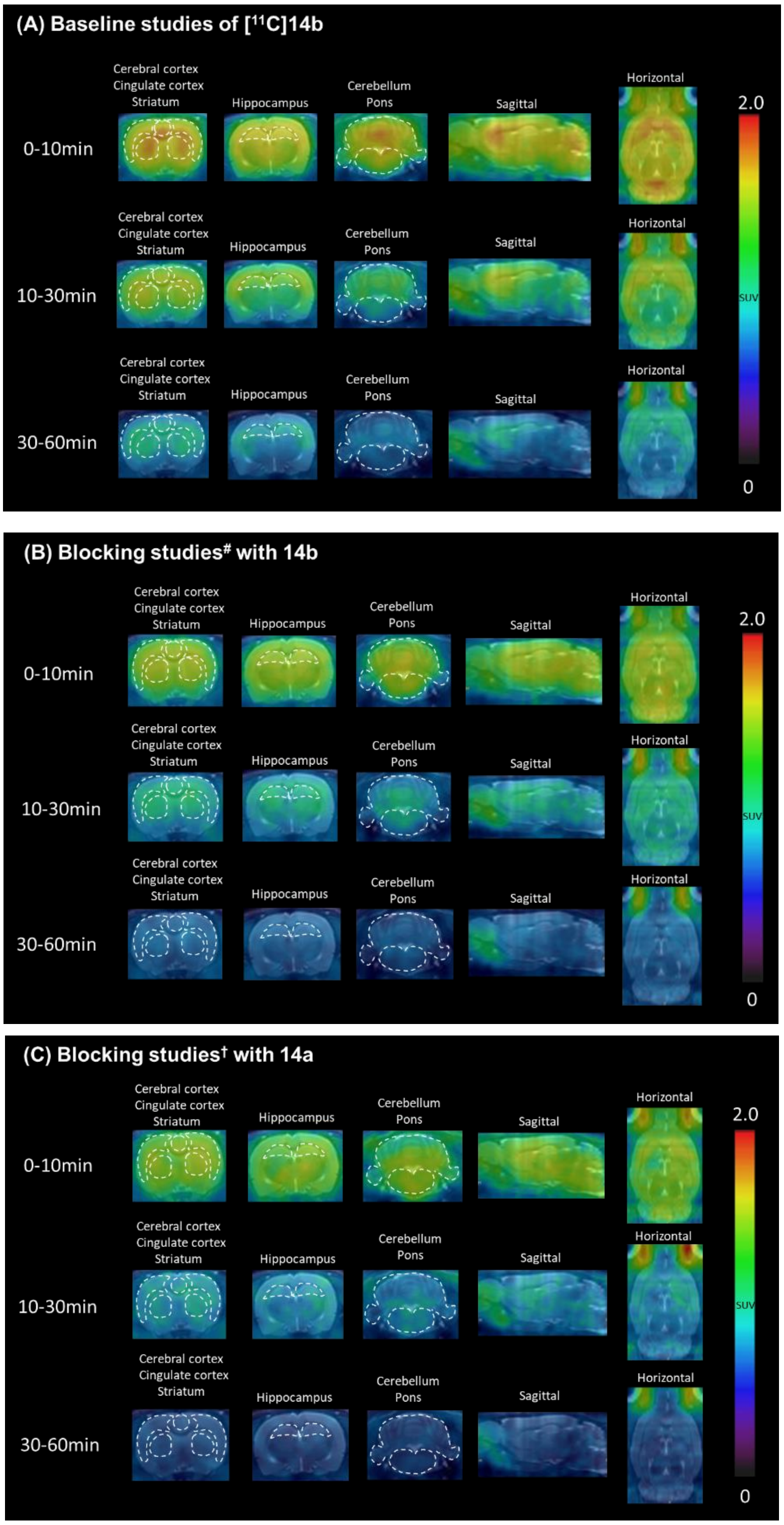

Figure S12. Representative PET/MRI fused images (summed at 0-10 min, 10-30 min and 30-60 min) and time-activity curves of [^{11}C]**14b** under baseline and blocking conditions in SD rat brain. #Blocking conditions: **14b** (1 mg/kg), 30 min *i.v.* before radioligand injection; †Blocking conditions: **14a** (3 mg/kg), 30 min *i.v.* before radioligand injection. Data are presented as mean ± SEM (n = 3).

NMR spectra of synthesized compounds

^{1}H spectrum of 11a

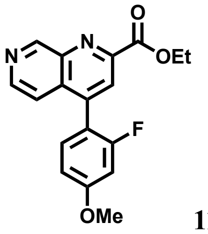

11a

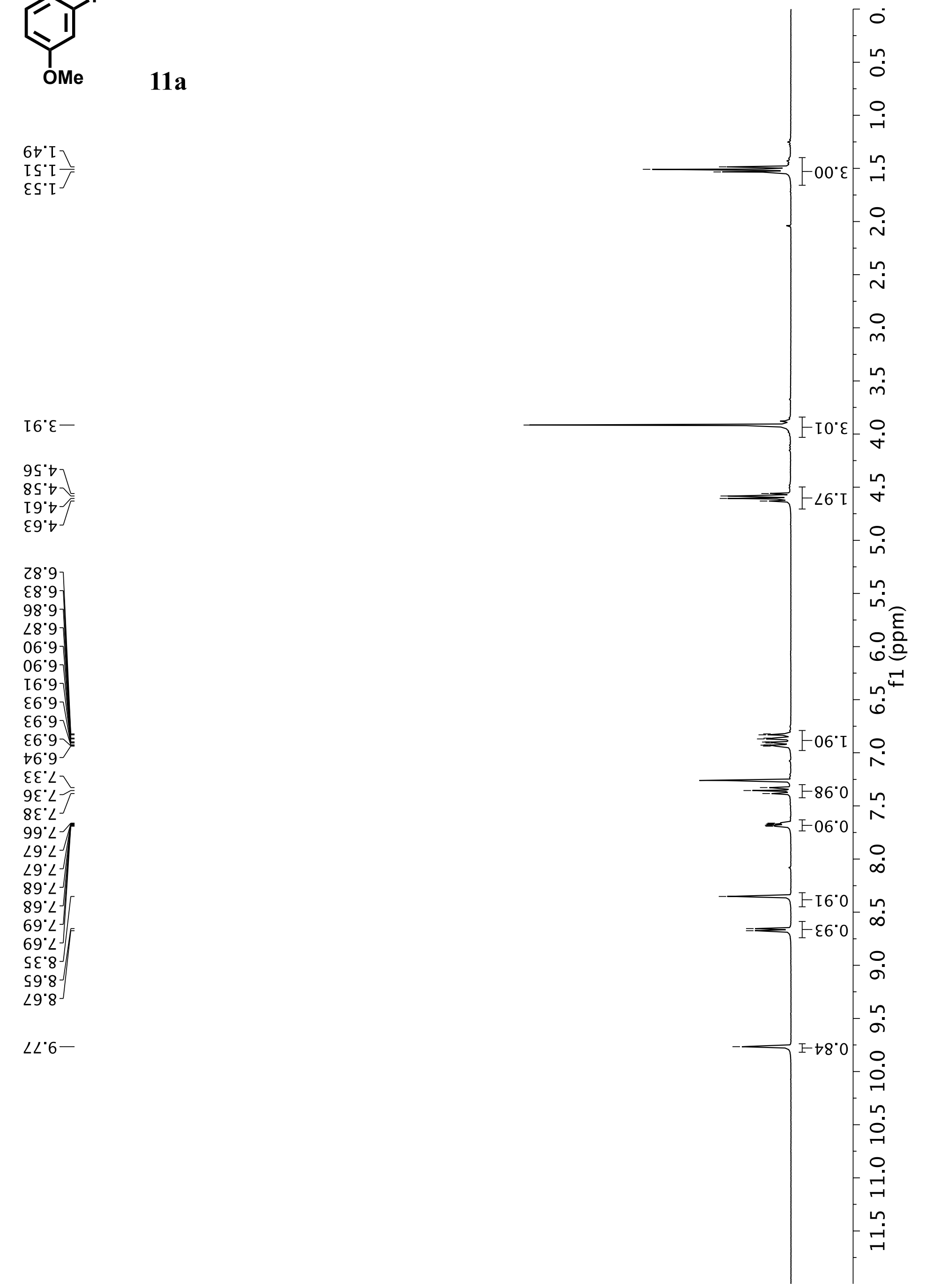

^{13}C spectrum of 11a

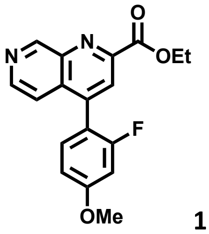

11a

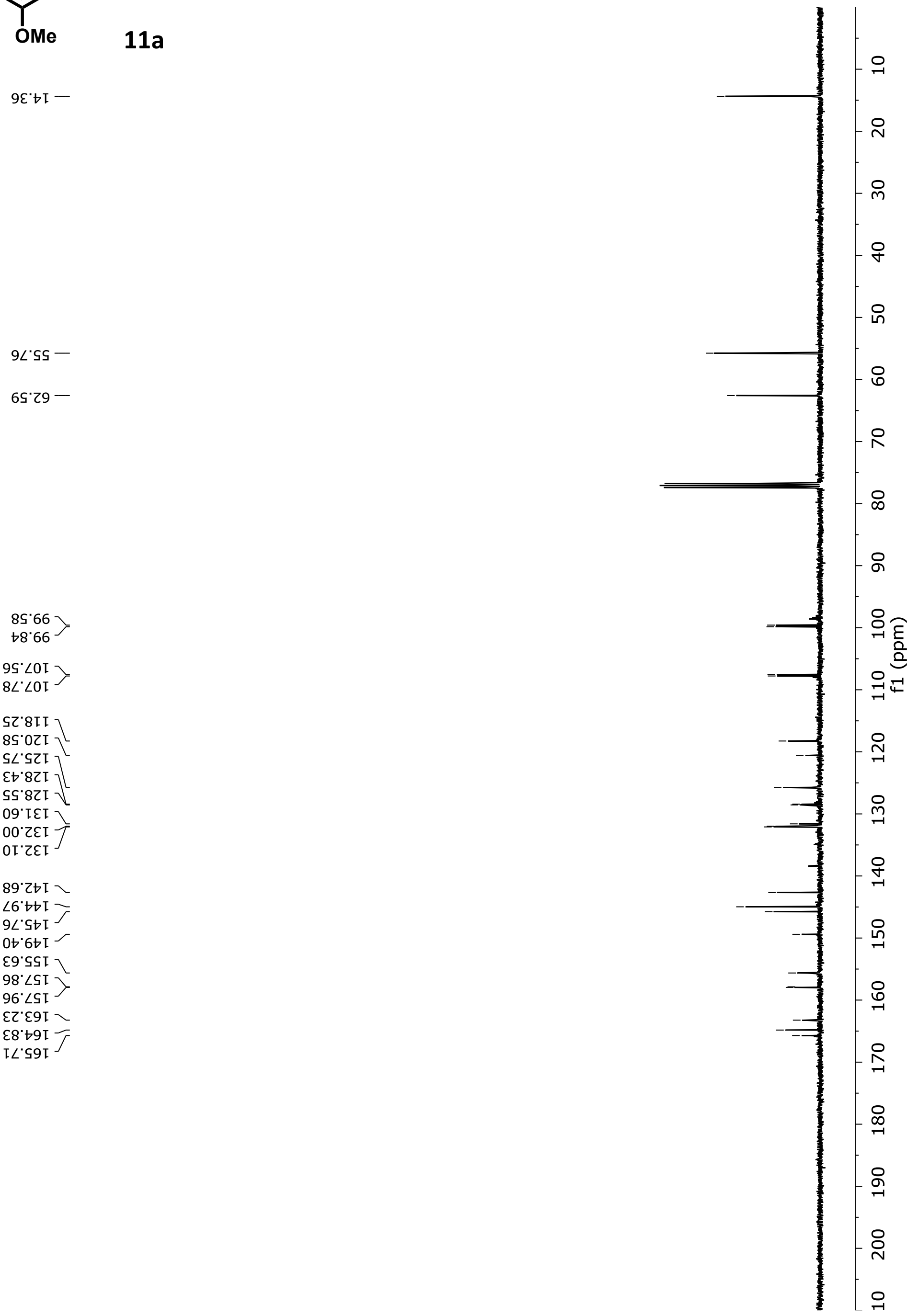

^{1}H spectrum of 11b

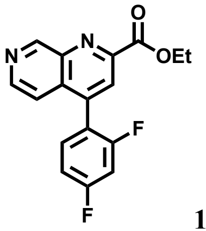

11b

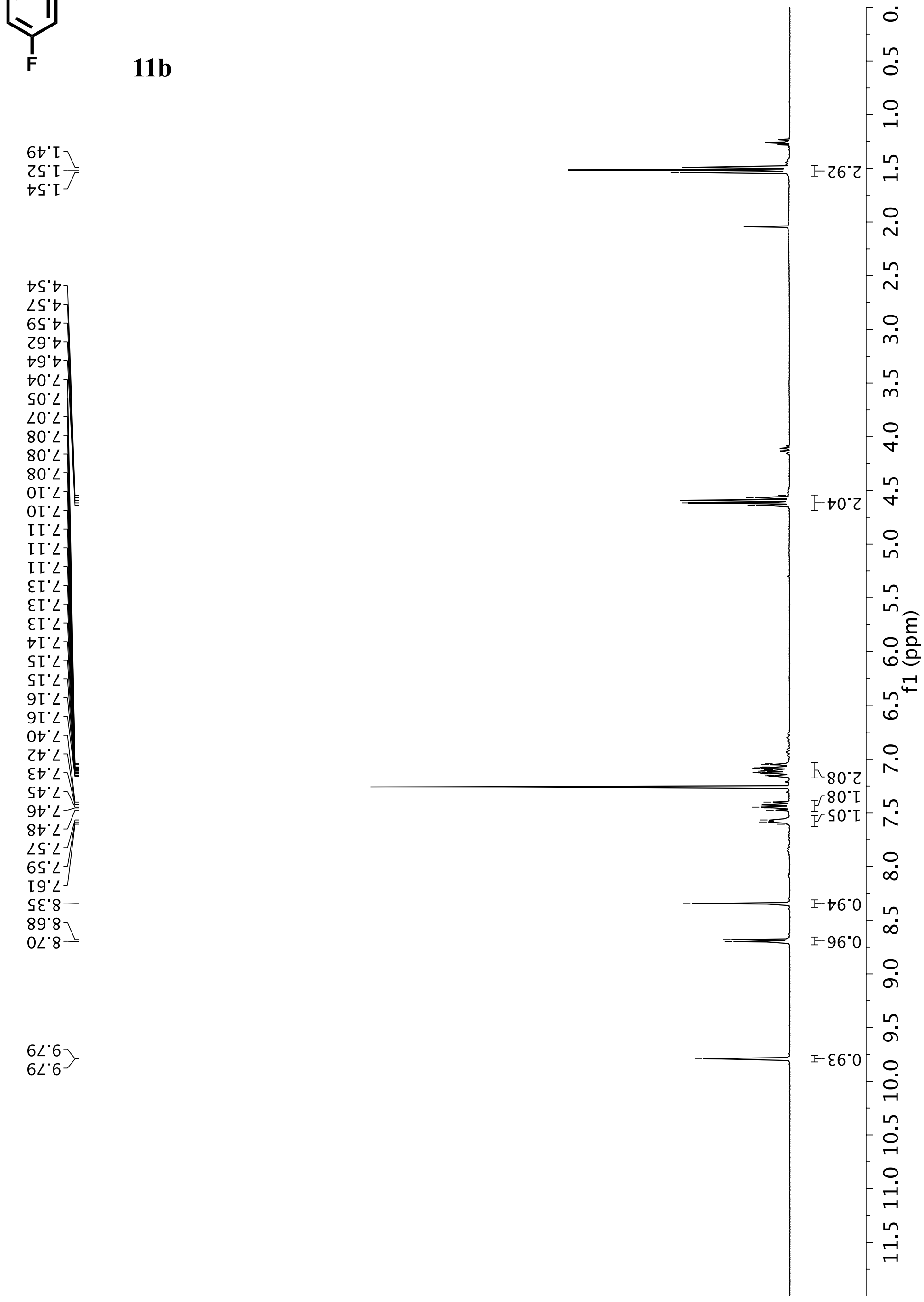

^{13}C spectrum of 11b

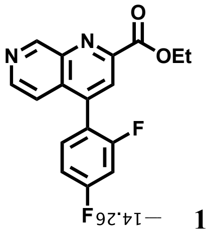

11b

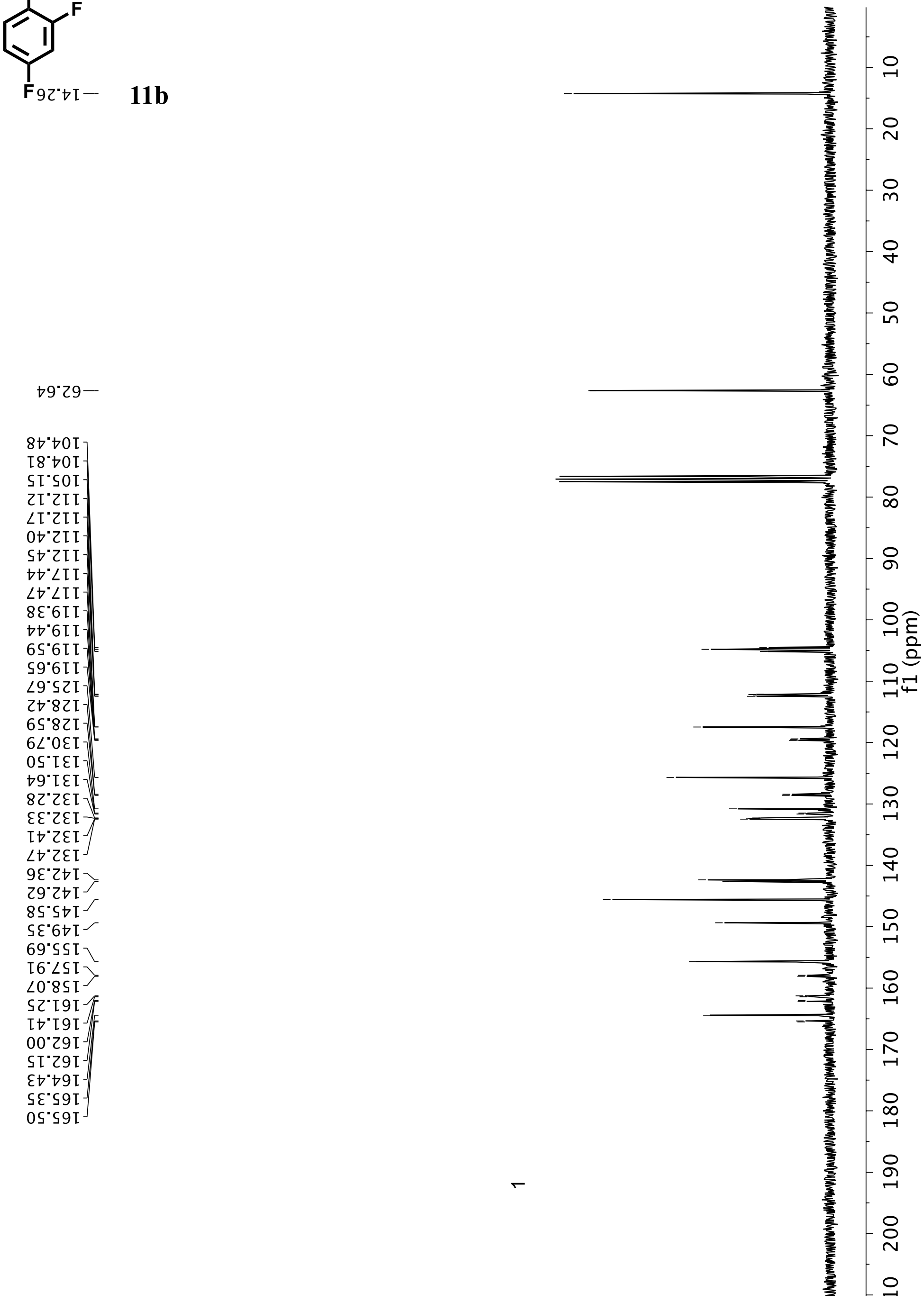

^{1}H spectrum of 11c

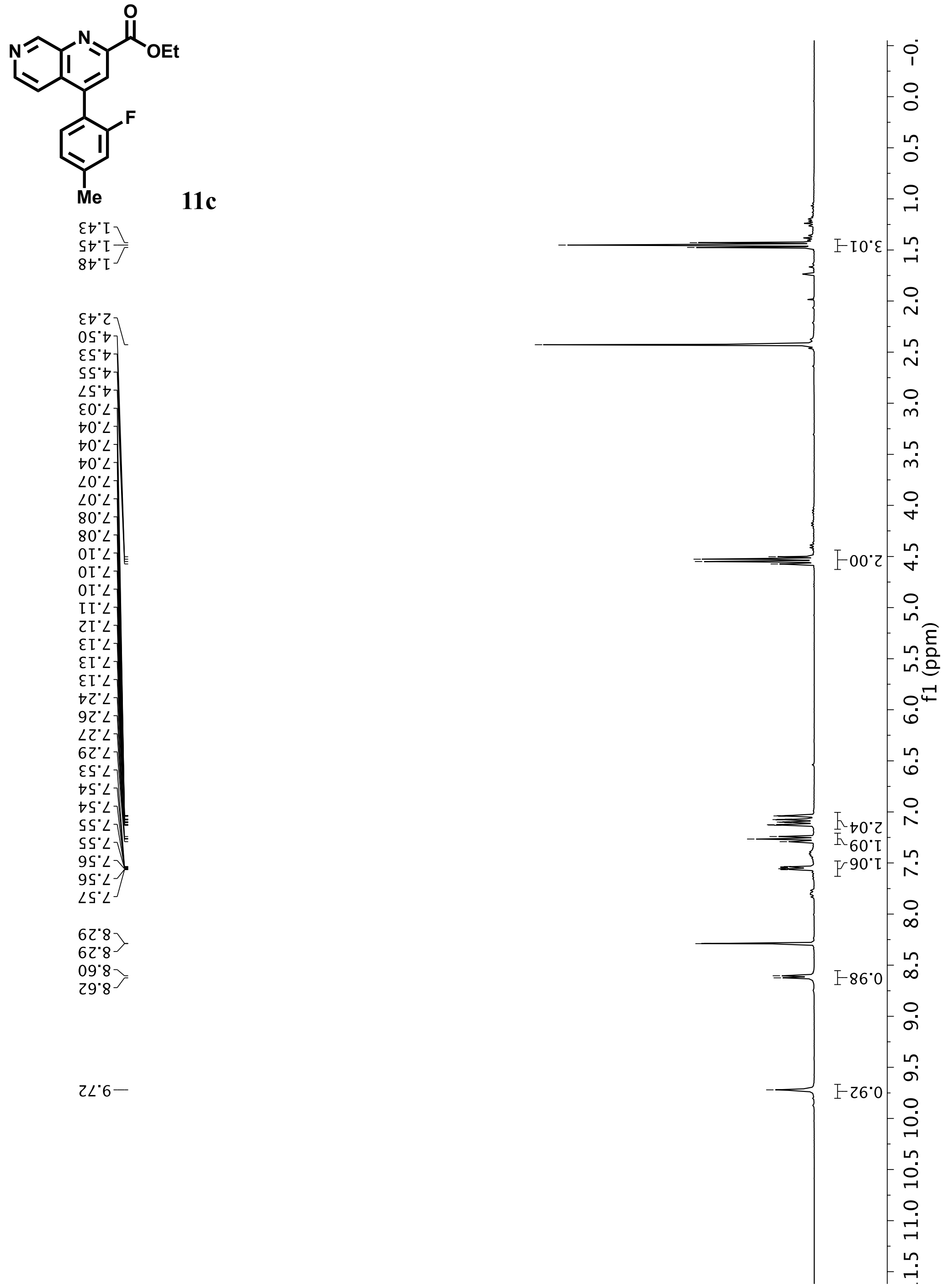

11c

^{13}C spectrum of 11c

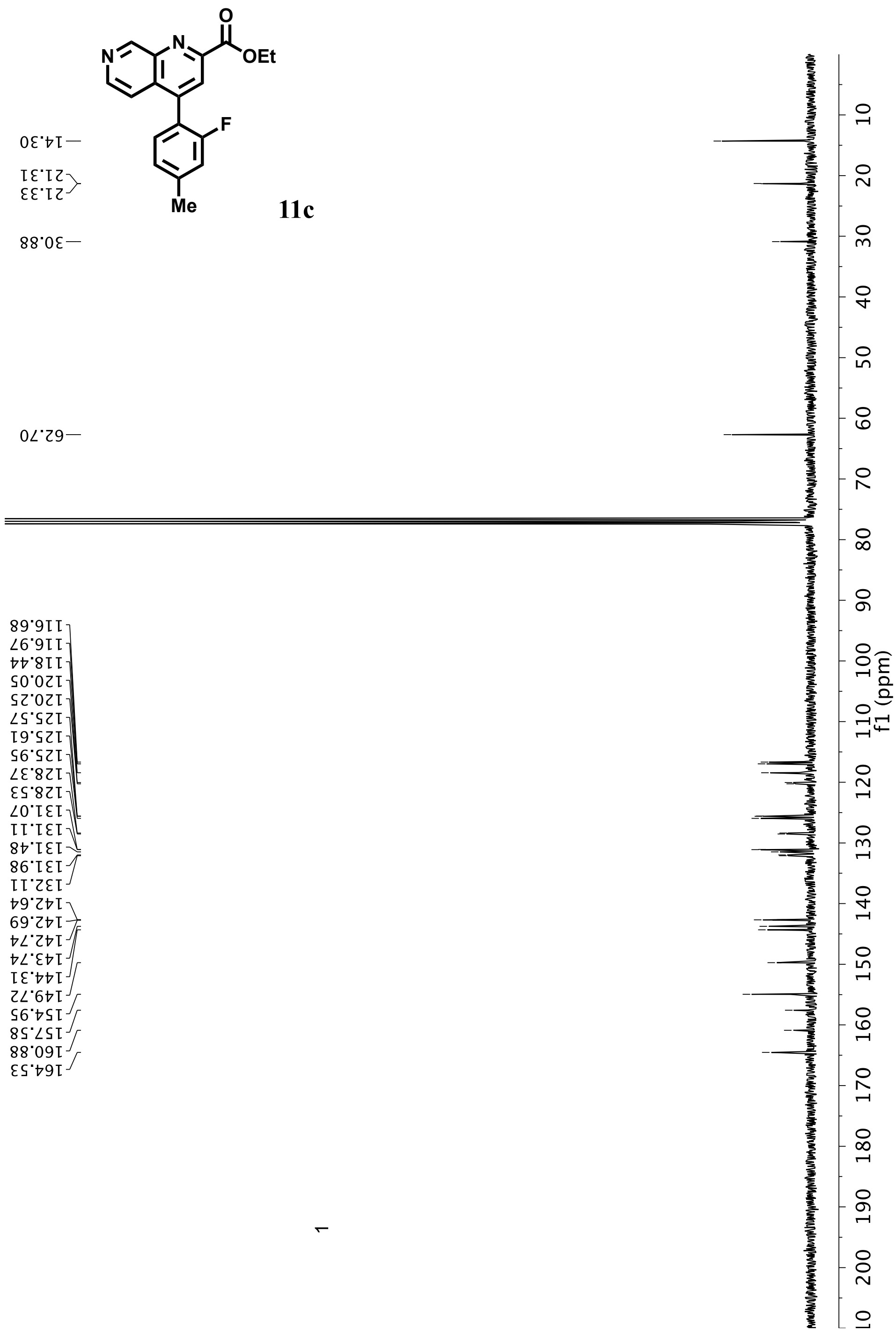

^{1}H spectrum of 11d

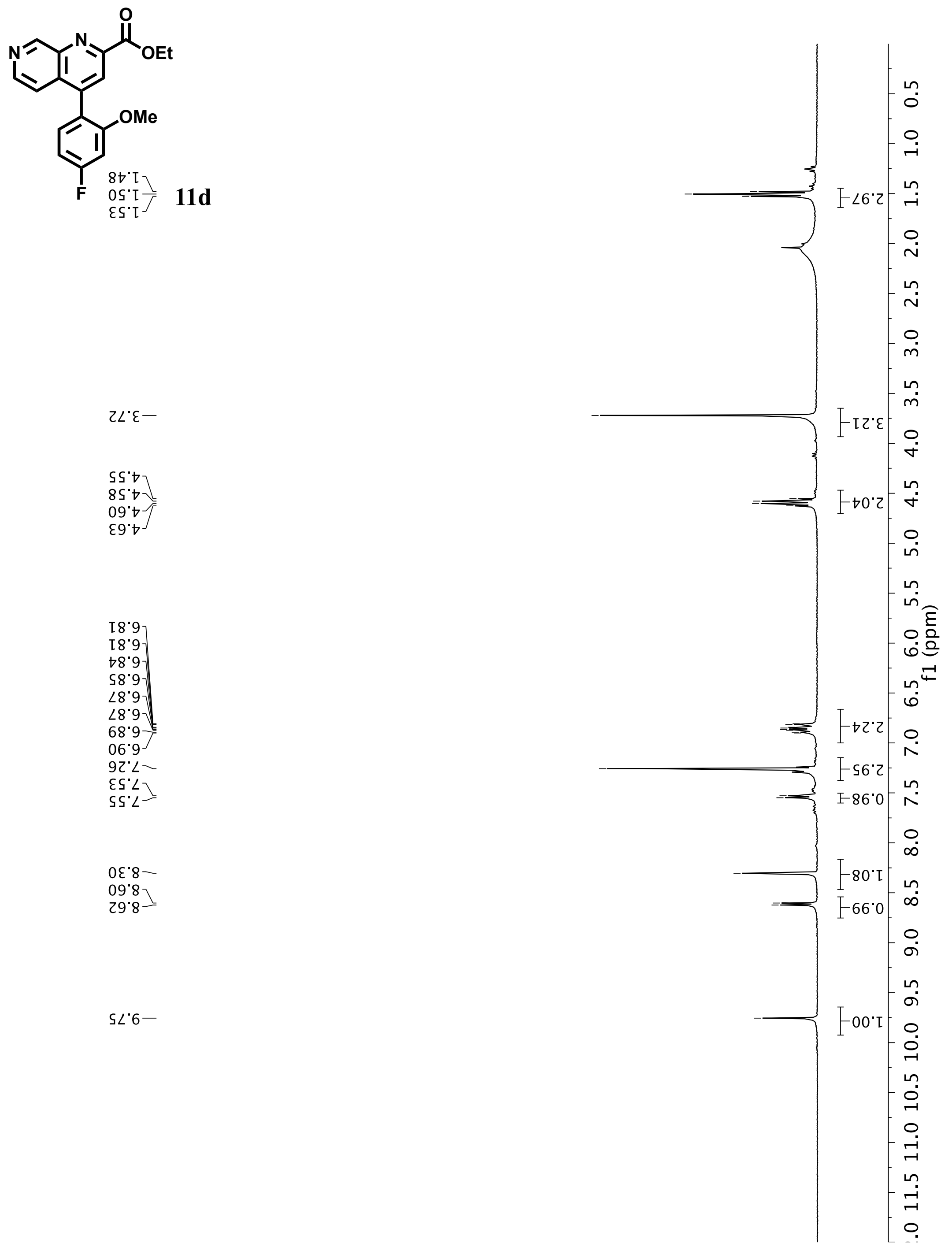

11d

^{13}C spectrum of 11d

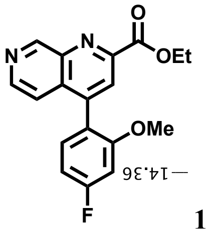

11d

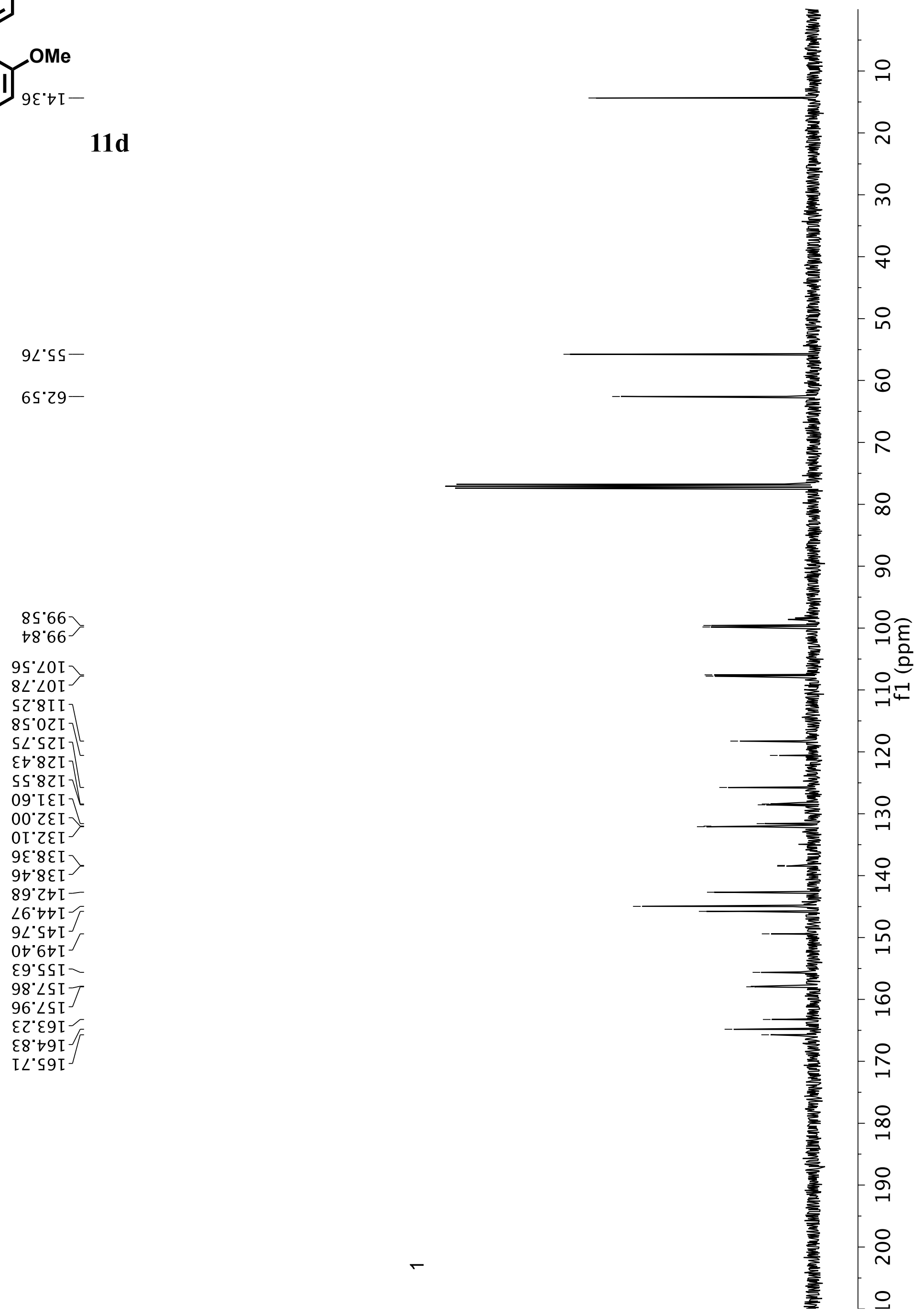

^{1}H spectrum of 11g

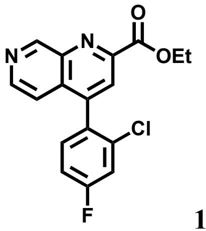

11g

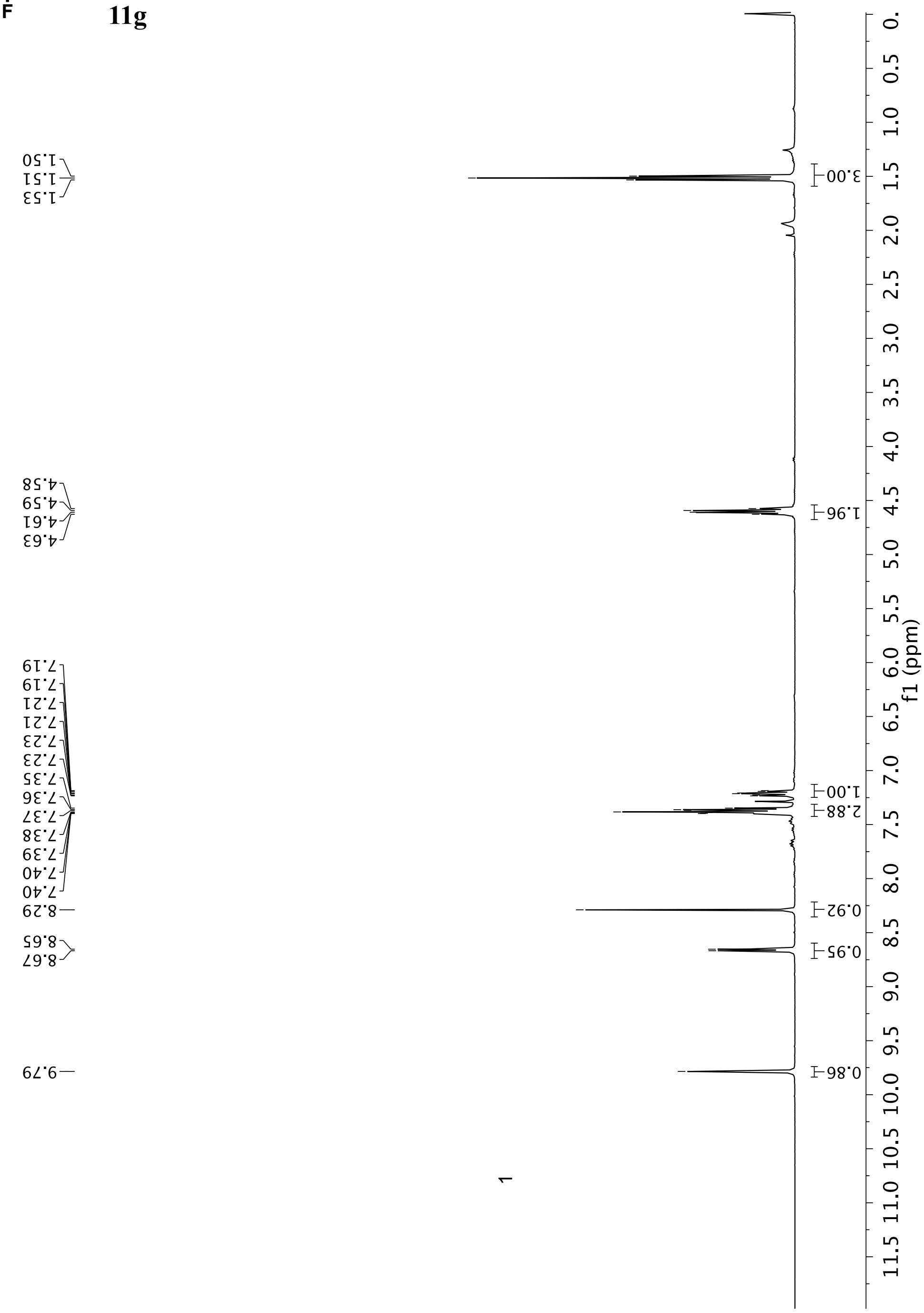

^{13}C spectrum of 11g

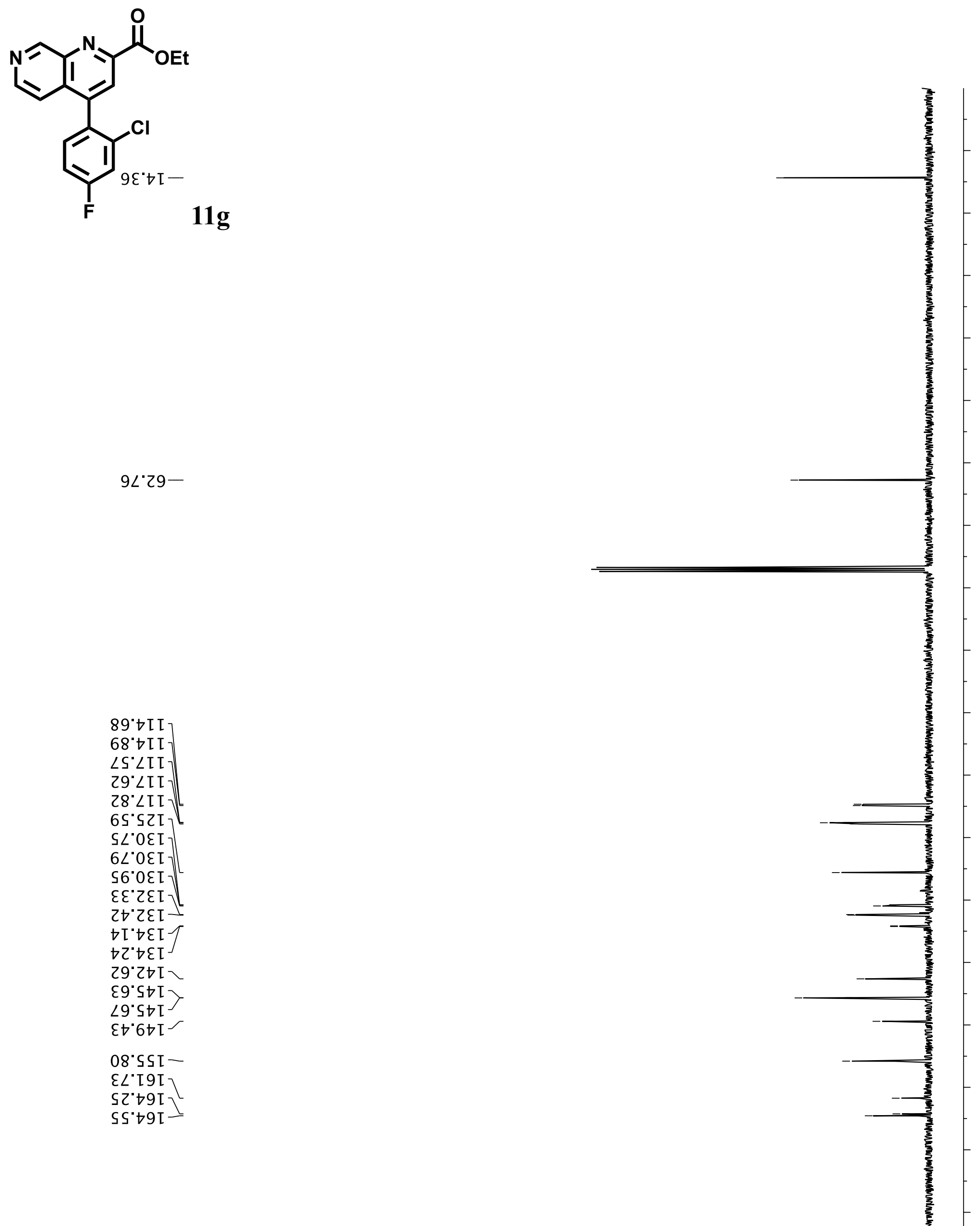

^{1}H spectrum of 13a

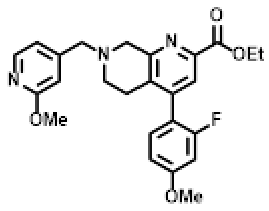

13a

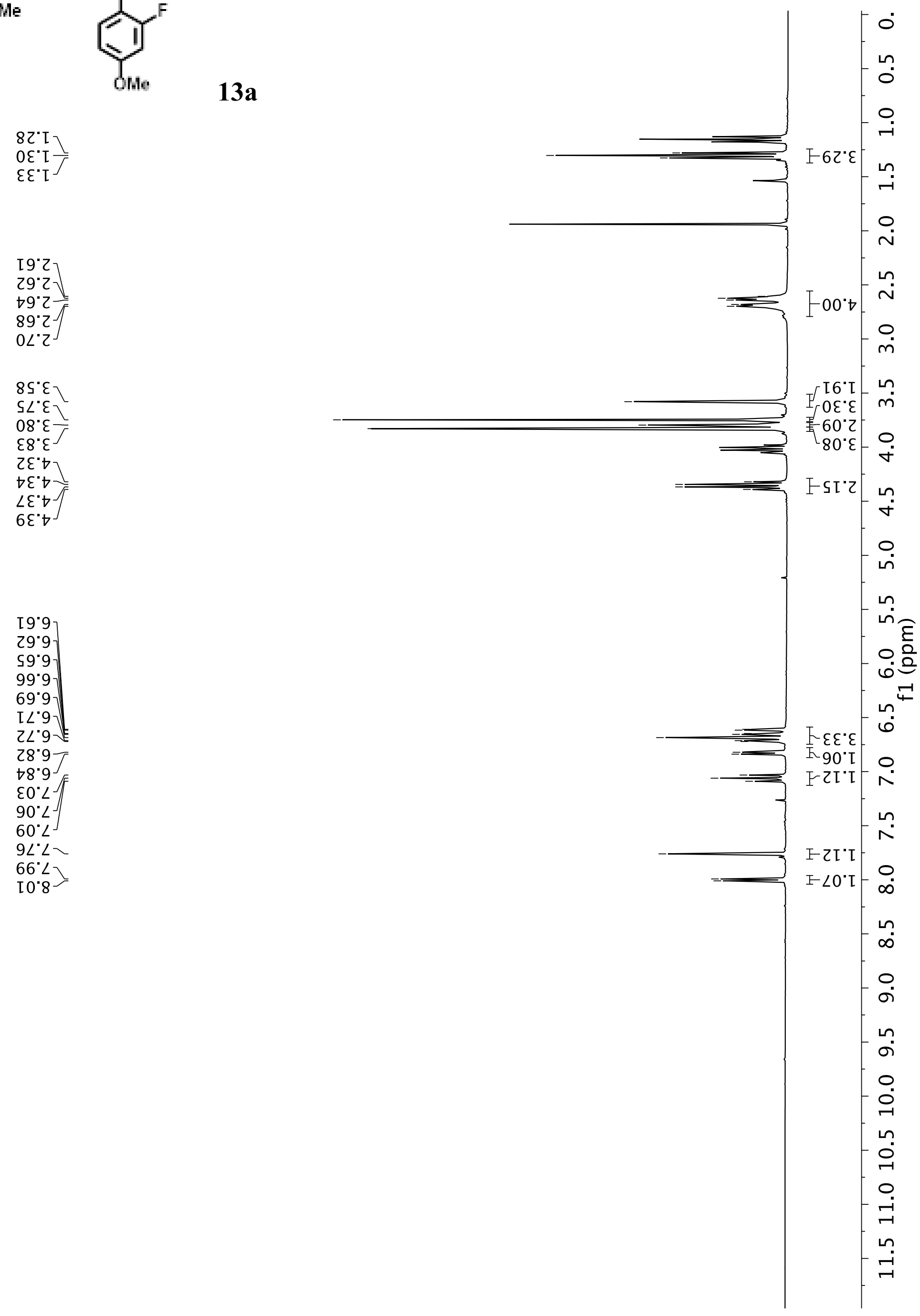

^{13}C spectrum of 13a

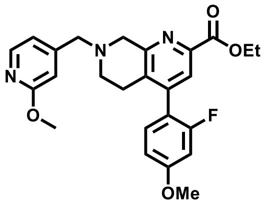

13a

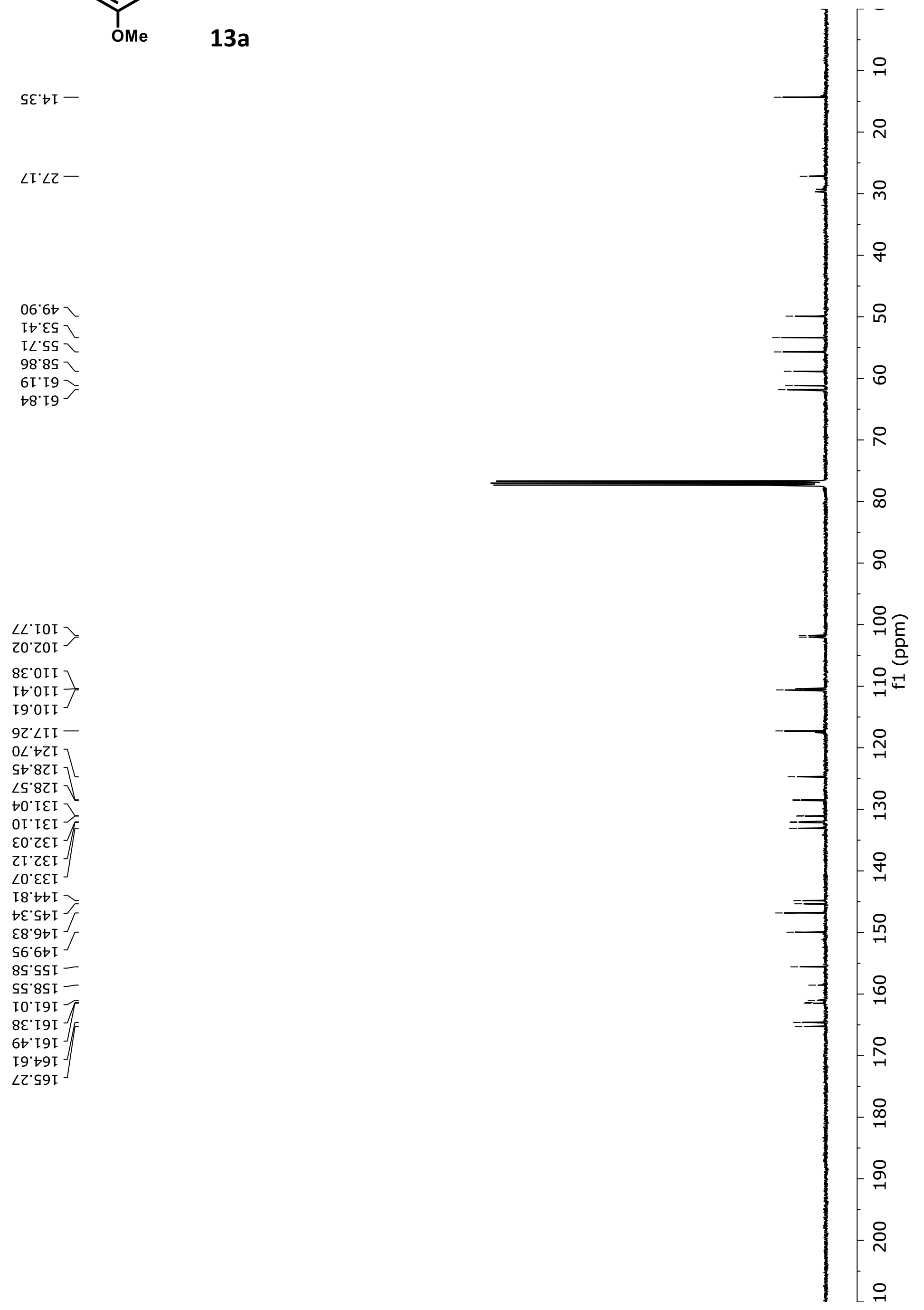

^{1}H spectrum of 13b

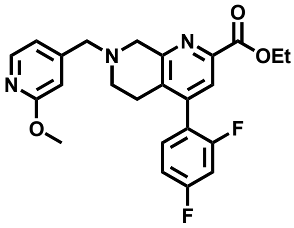

13b

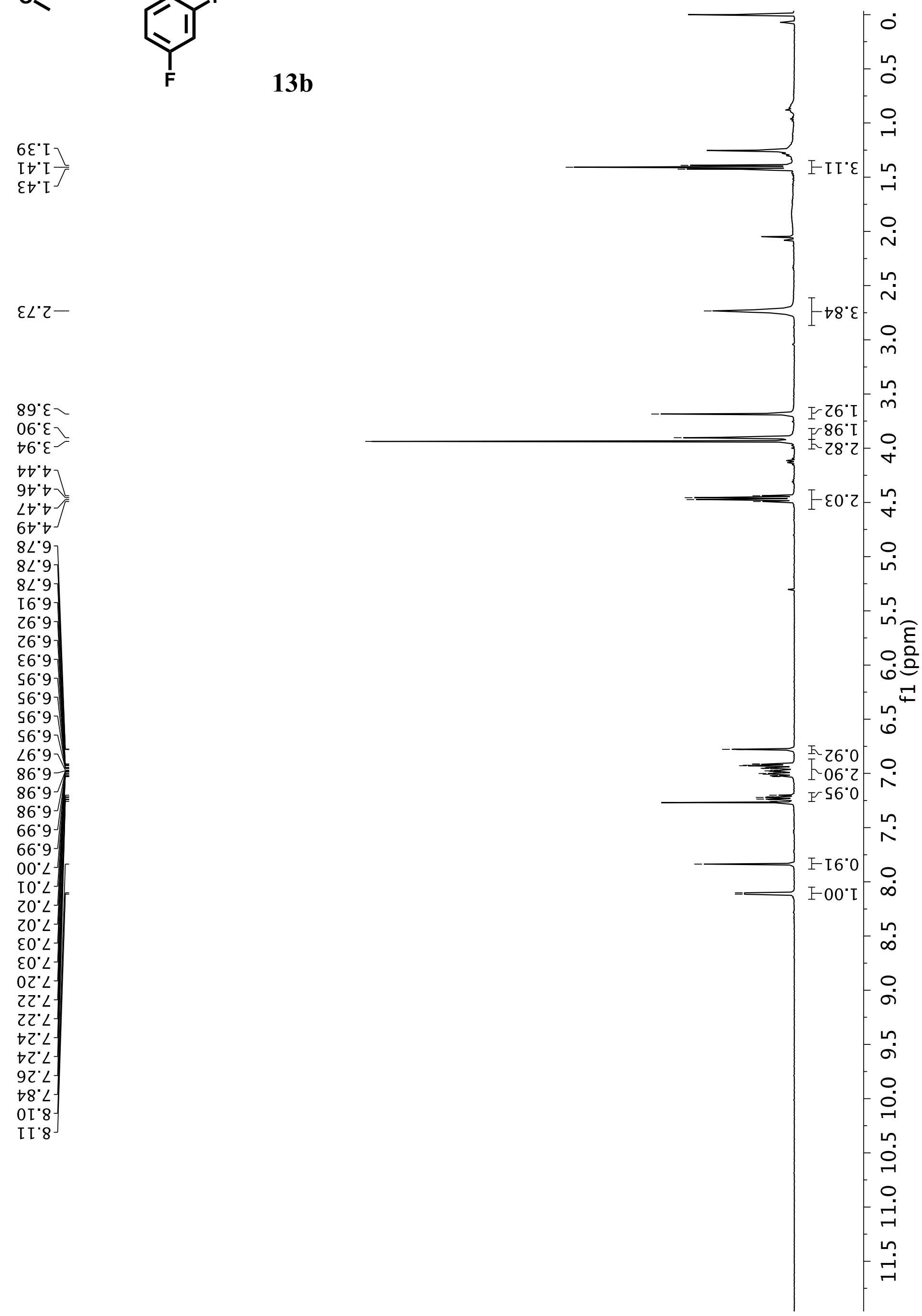

^{13}C spectrum of 13b

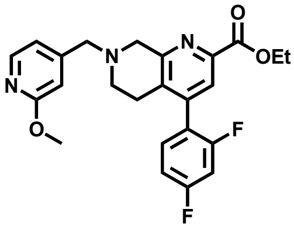

13b

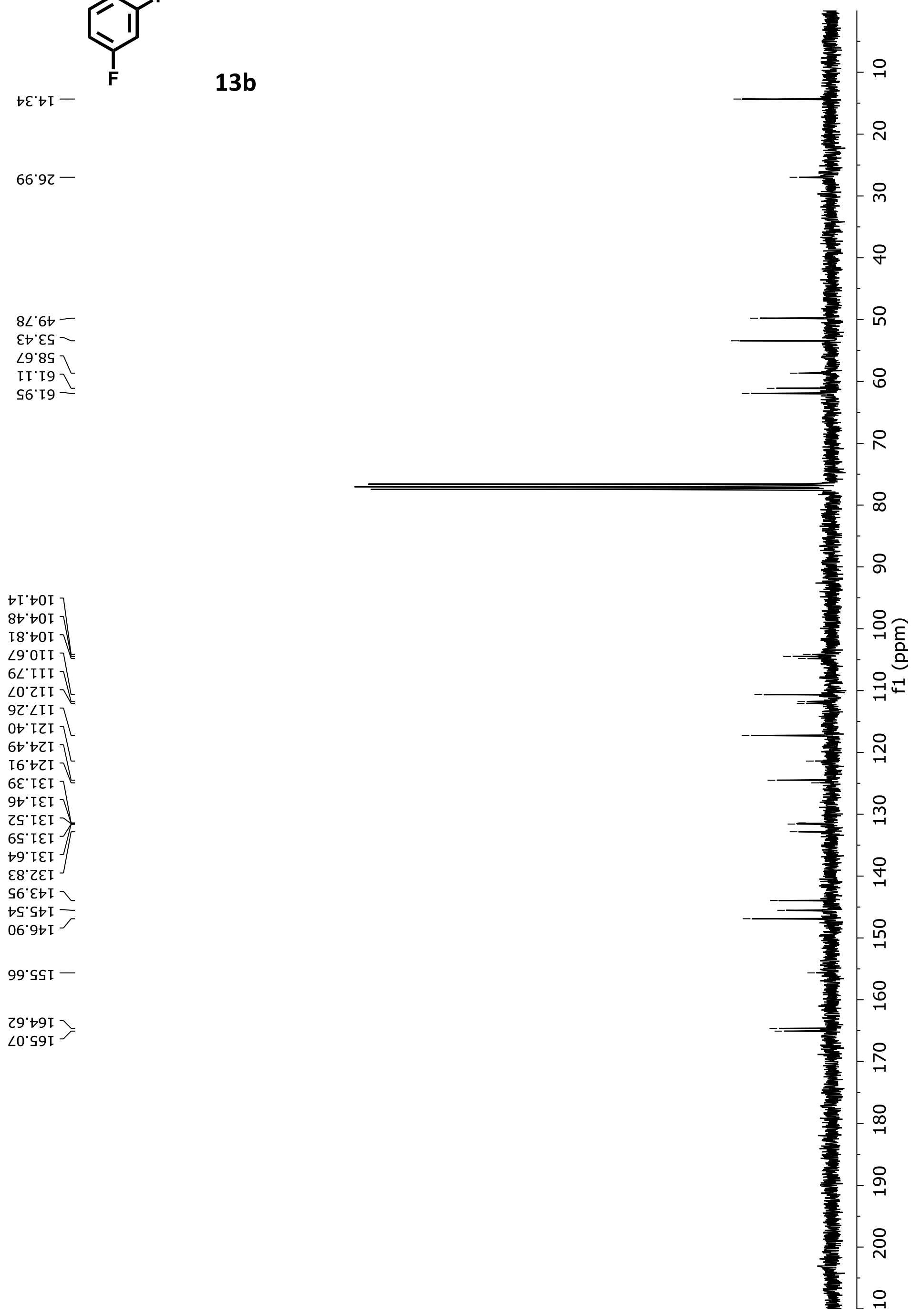

^{1}H spectrum of 13c

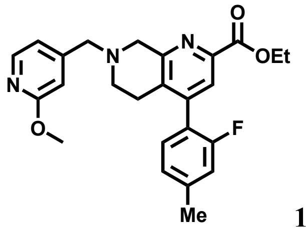

13c

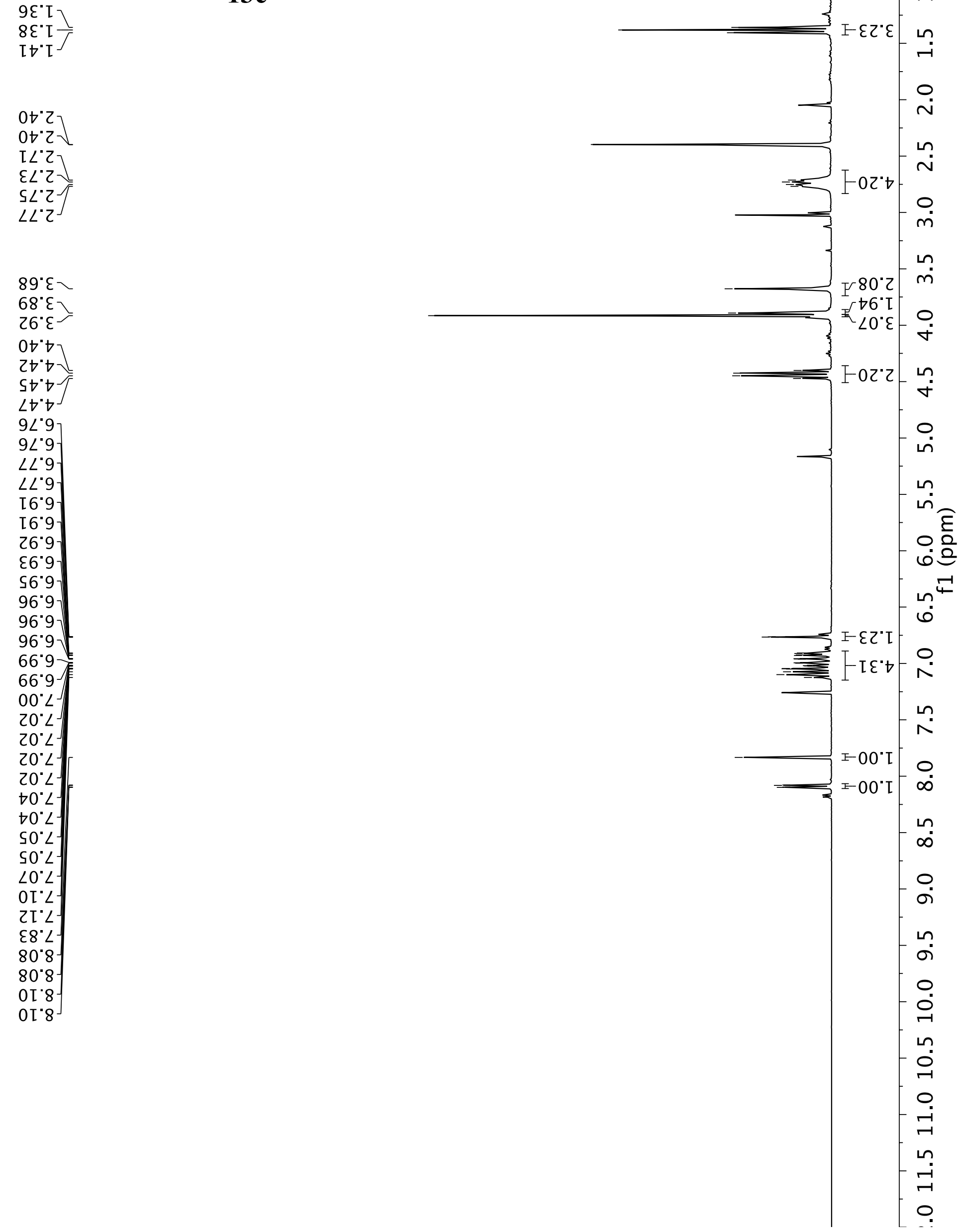

^{13}C spectrum of 13c

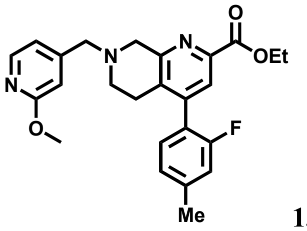

13c

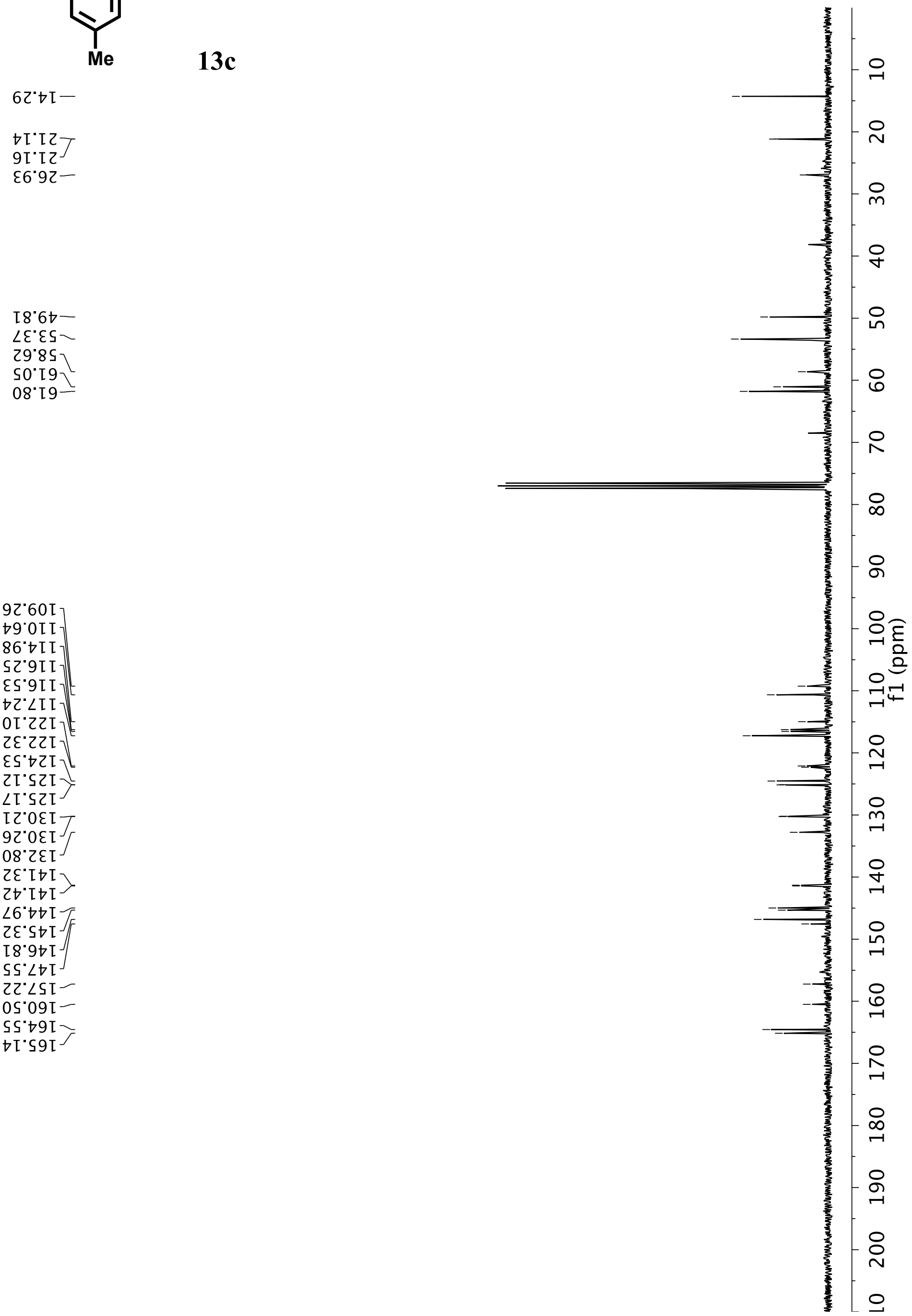

^{1}H spectrum of 13d

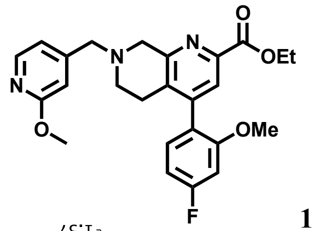

13d

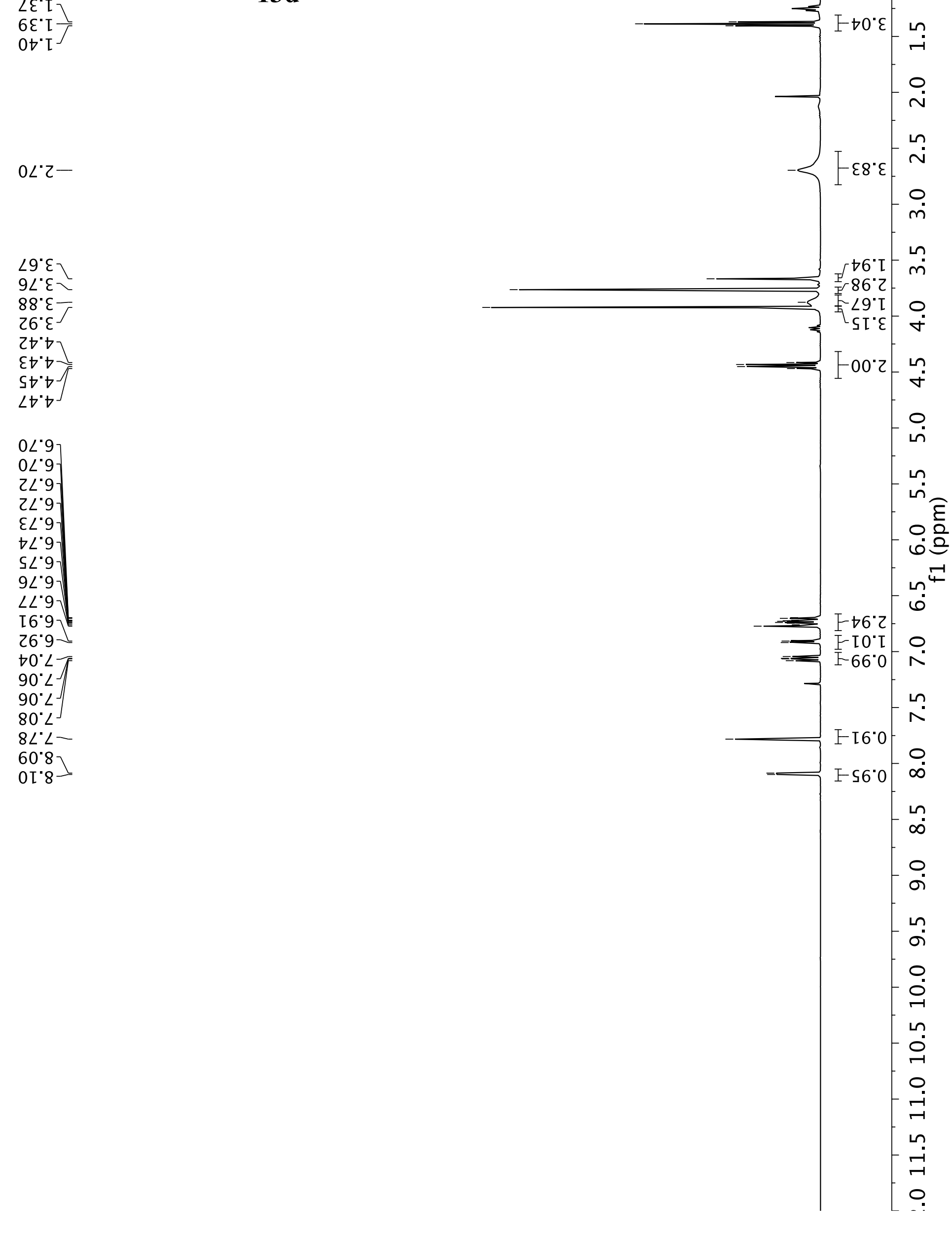

^{13}C spectrum of 13d

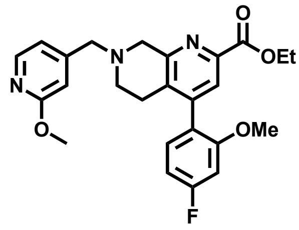

13d

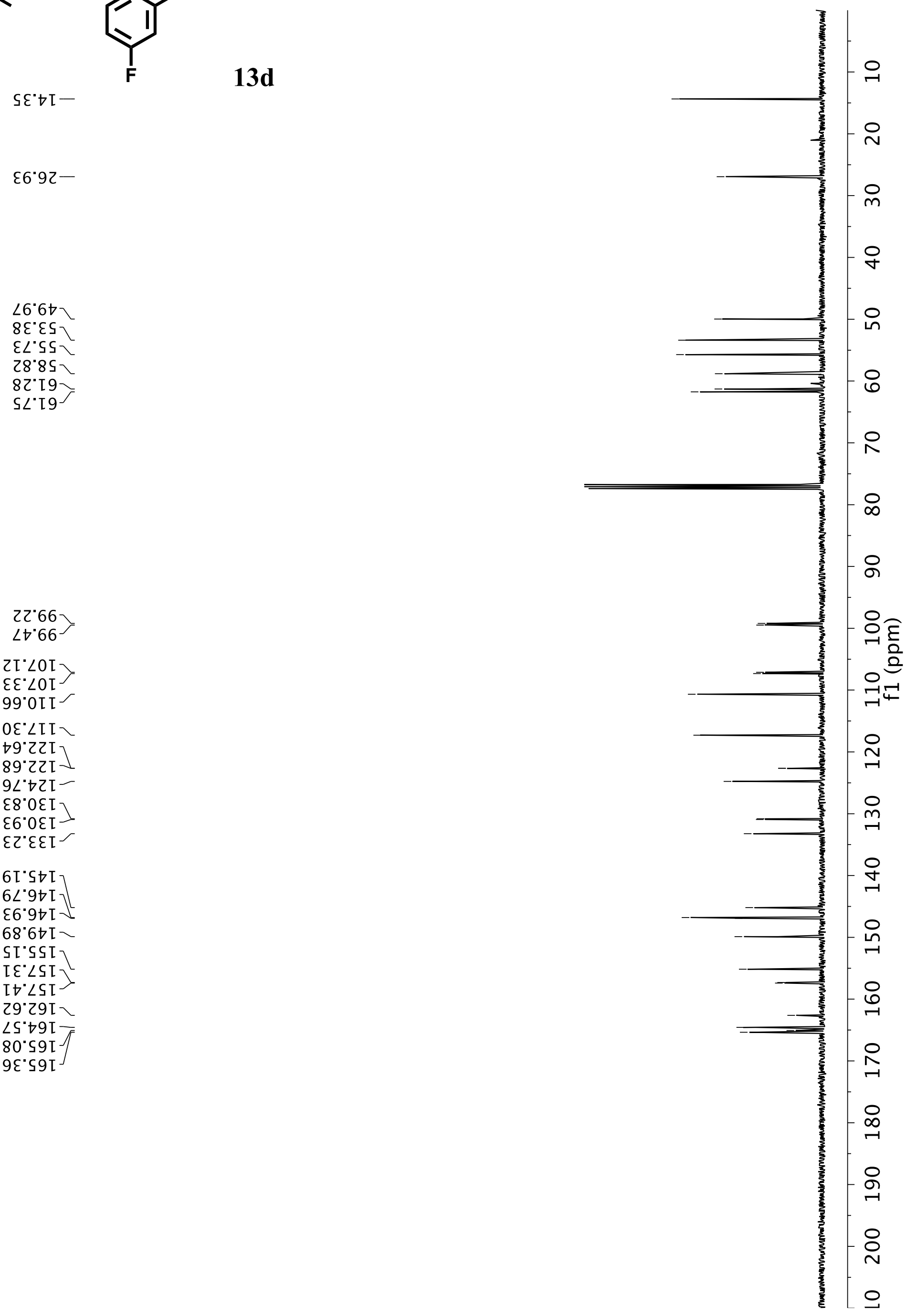

^{1}H spectrum of 13e

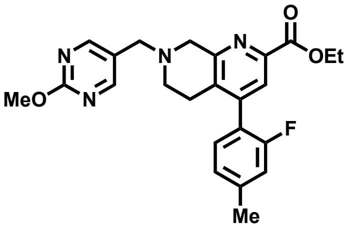

13e

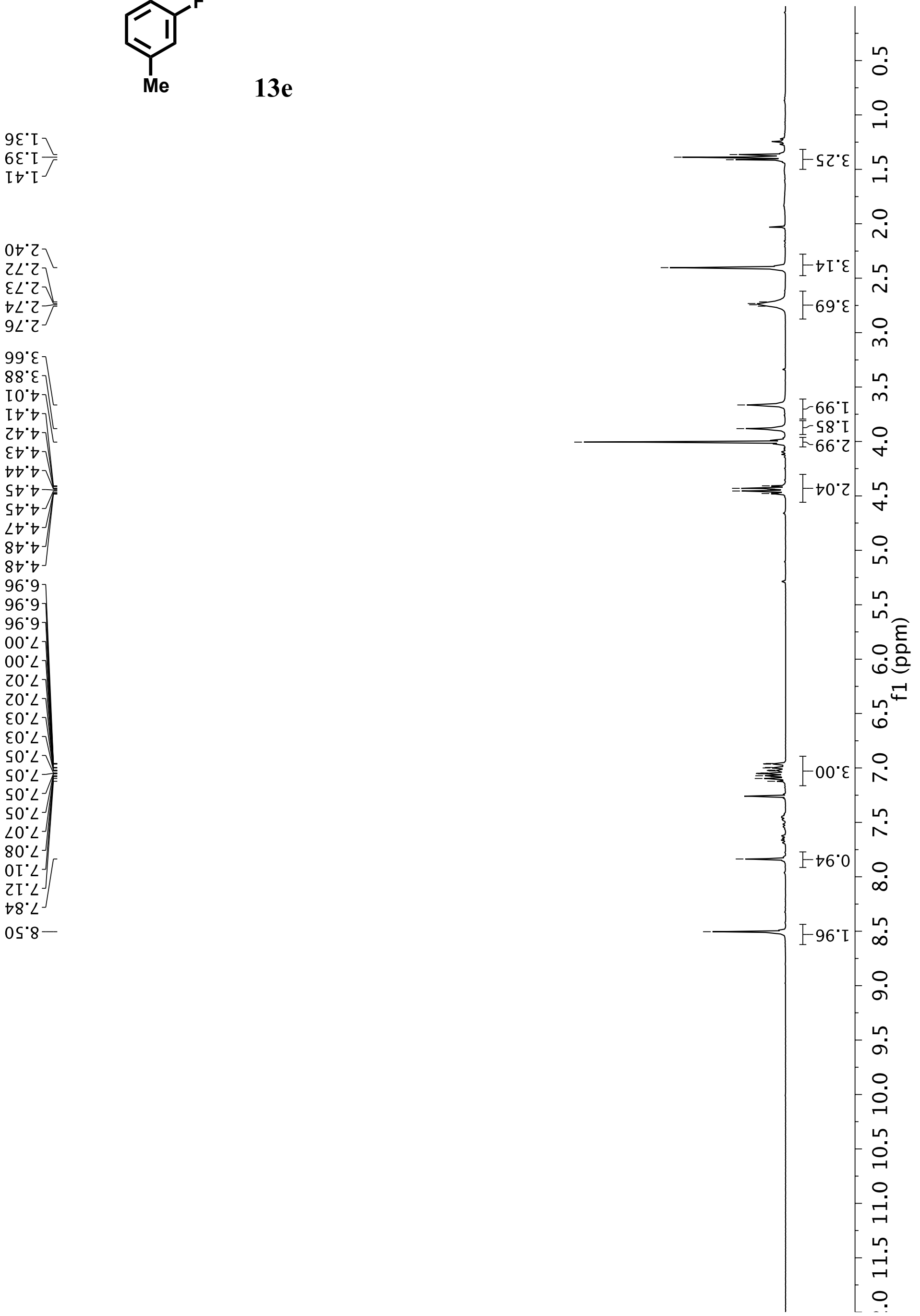

^{13}C spectrum of 13e

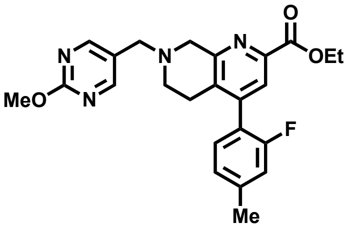

13e

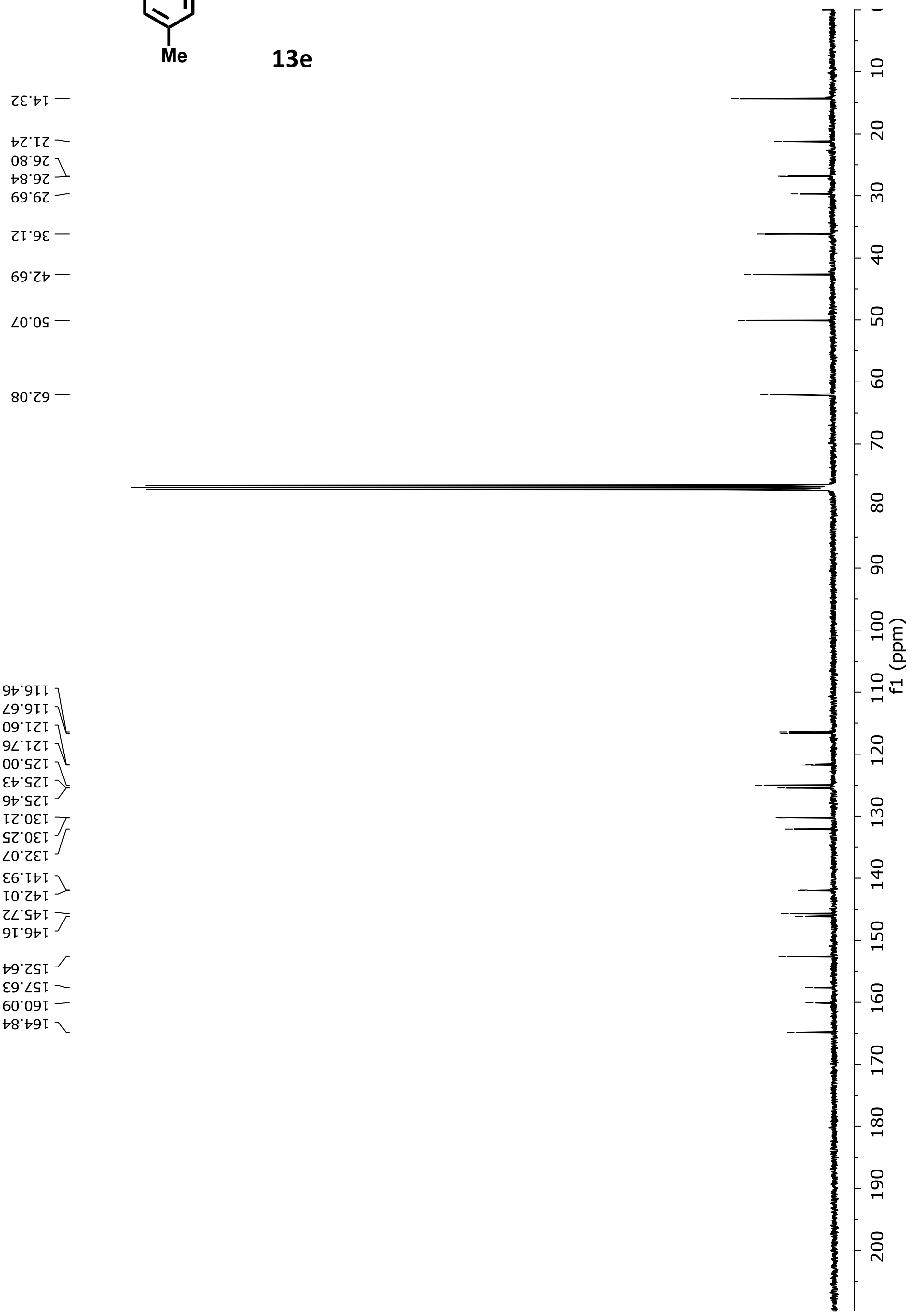

^{1}H spectrum of 13f

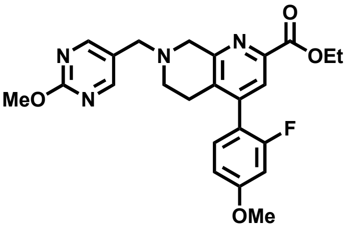

13f

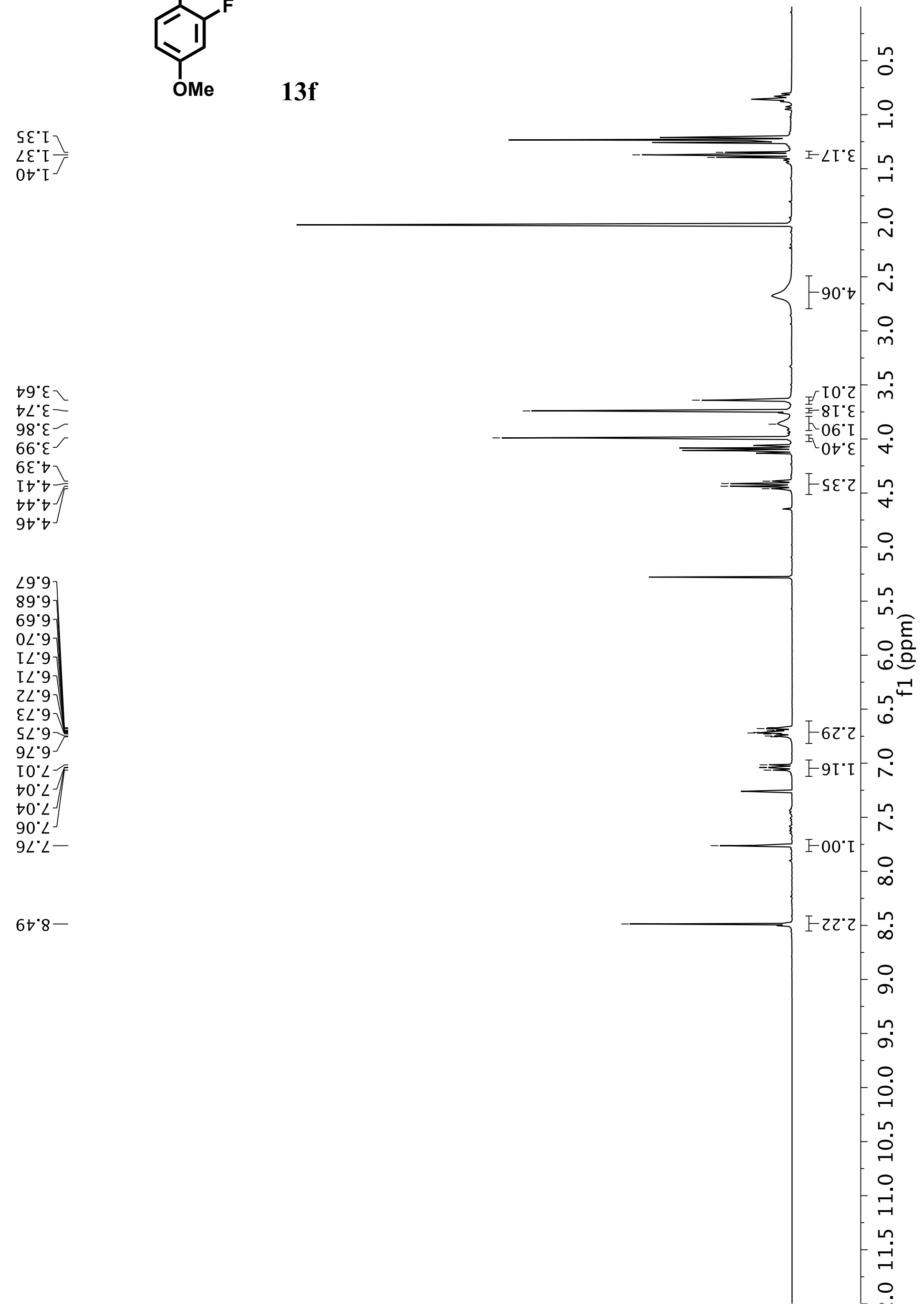

^{13}C spectrum of 13f

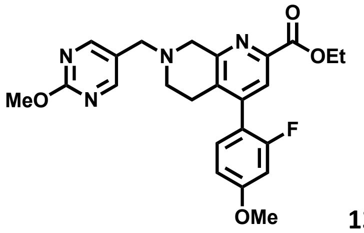

13f

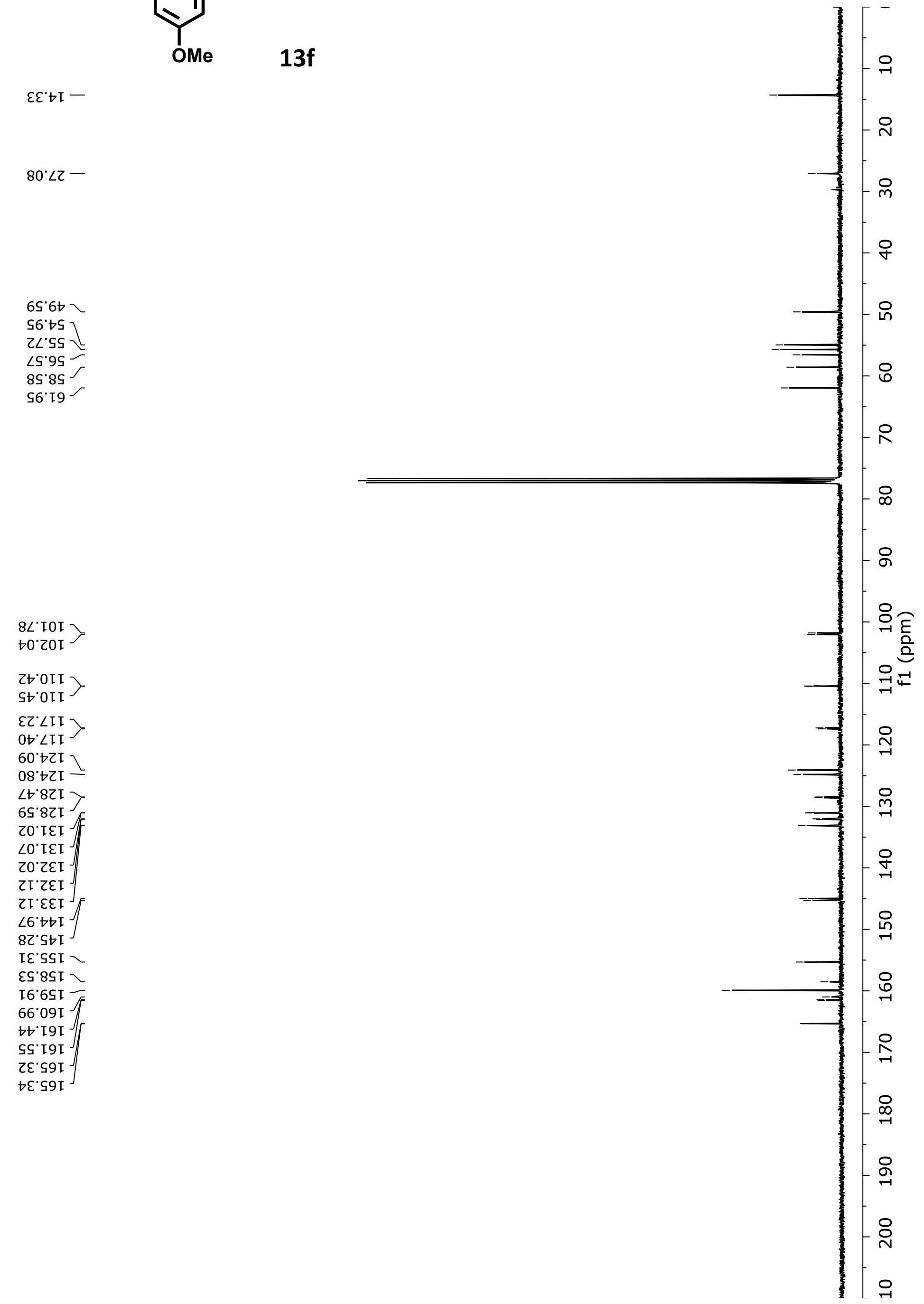

^{1}H spectrum of 13g

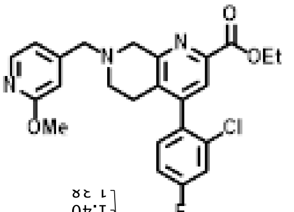

13g

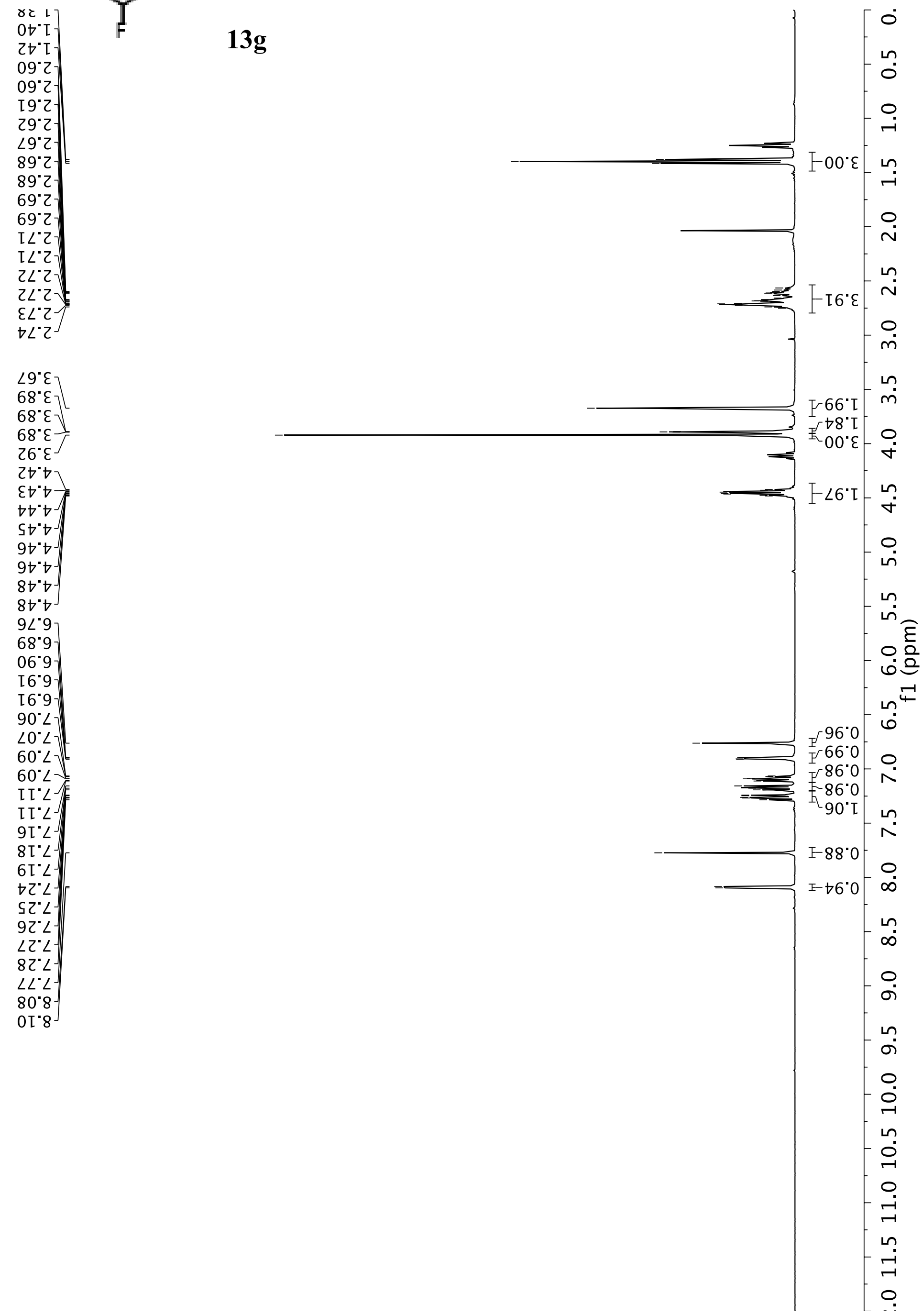

^{13}C spectrum of 13g

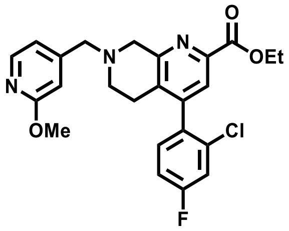

13g

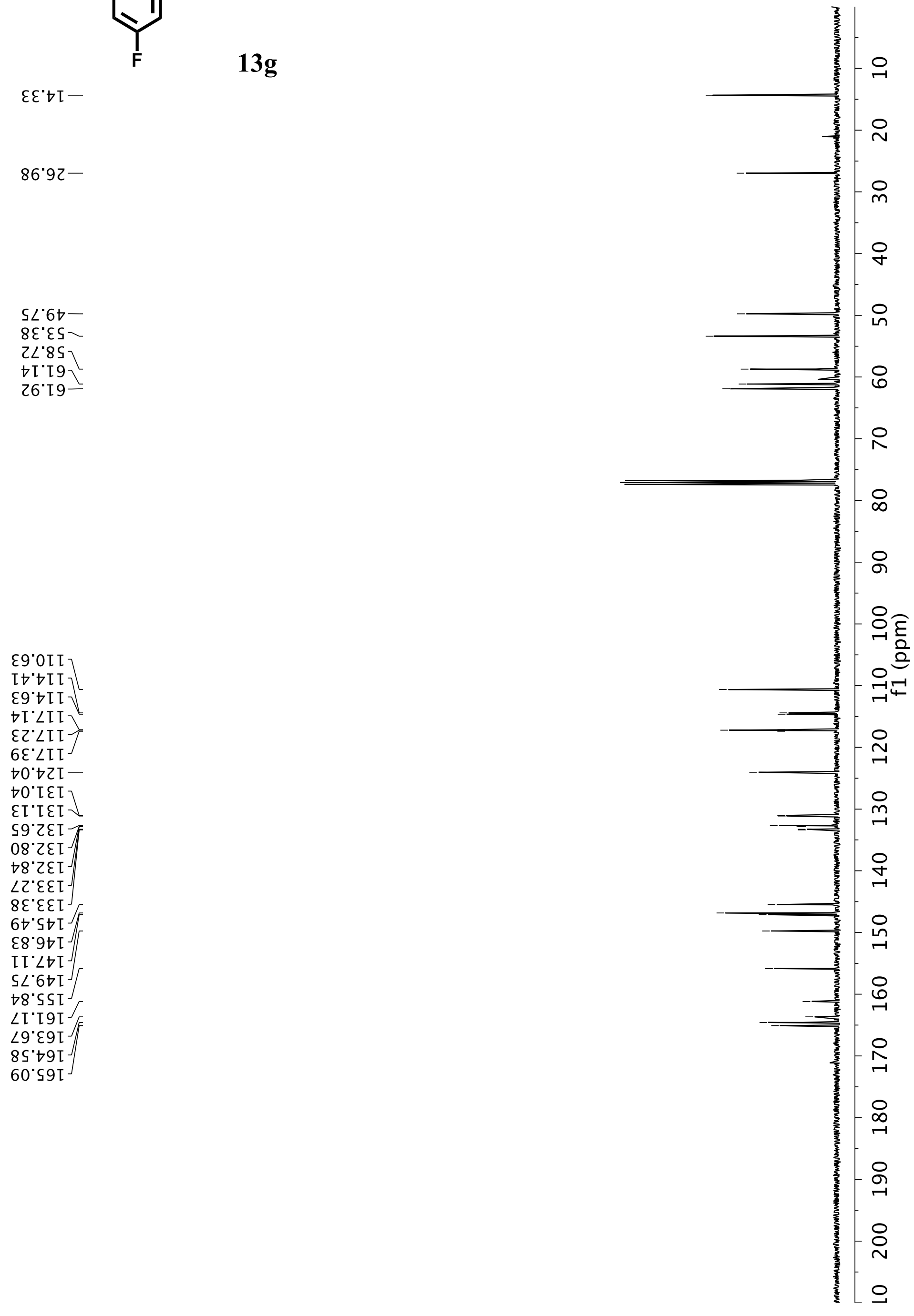

^{1}H spectrum of 14a

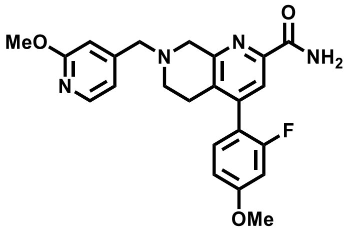

14a

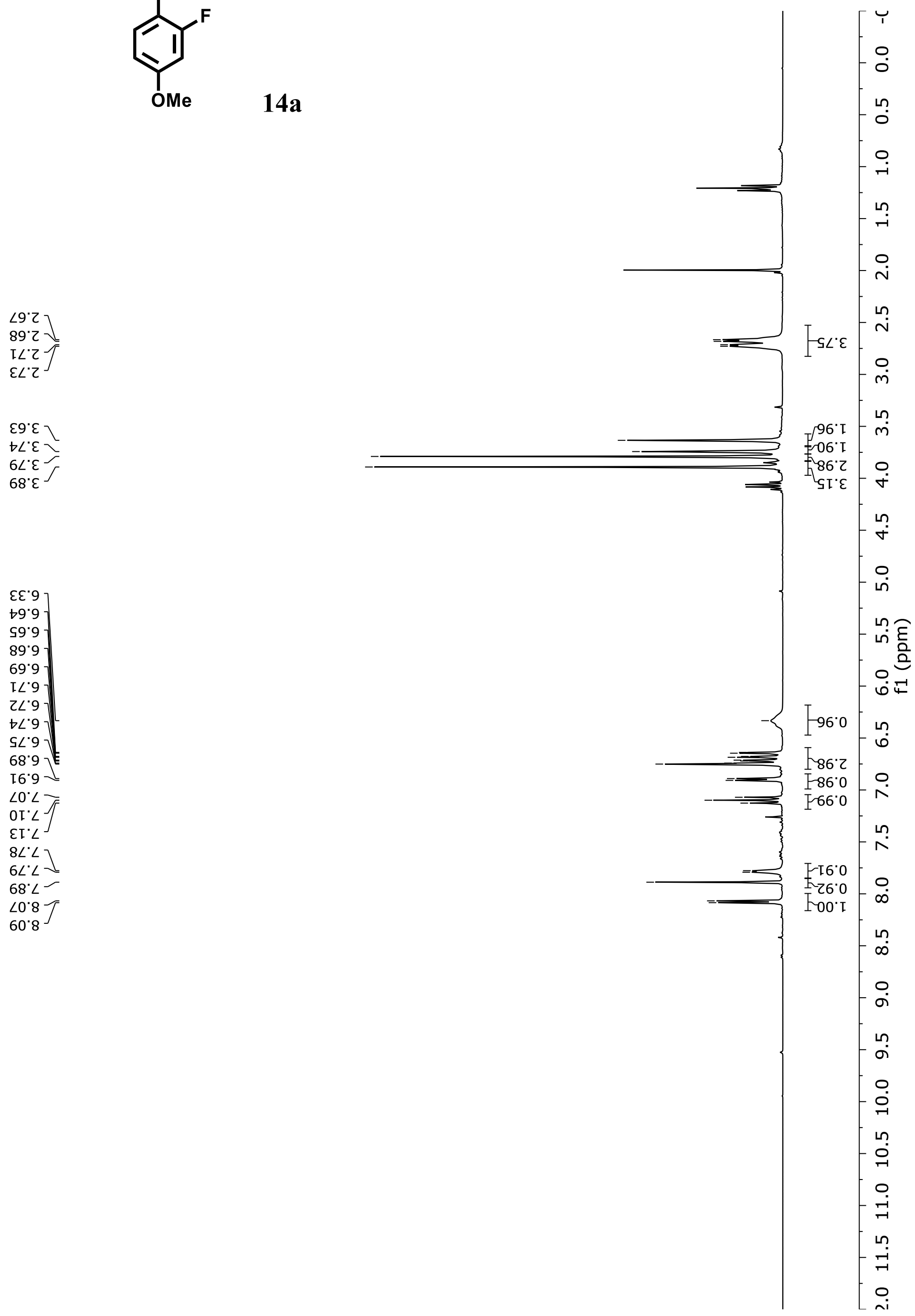

^{13}C spectrum of 14a

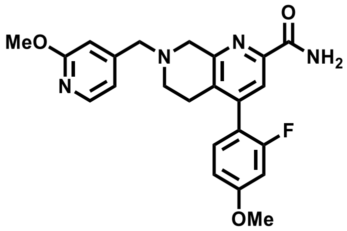

14a

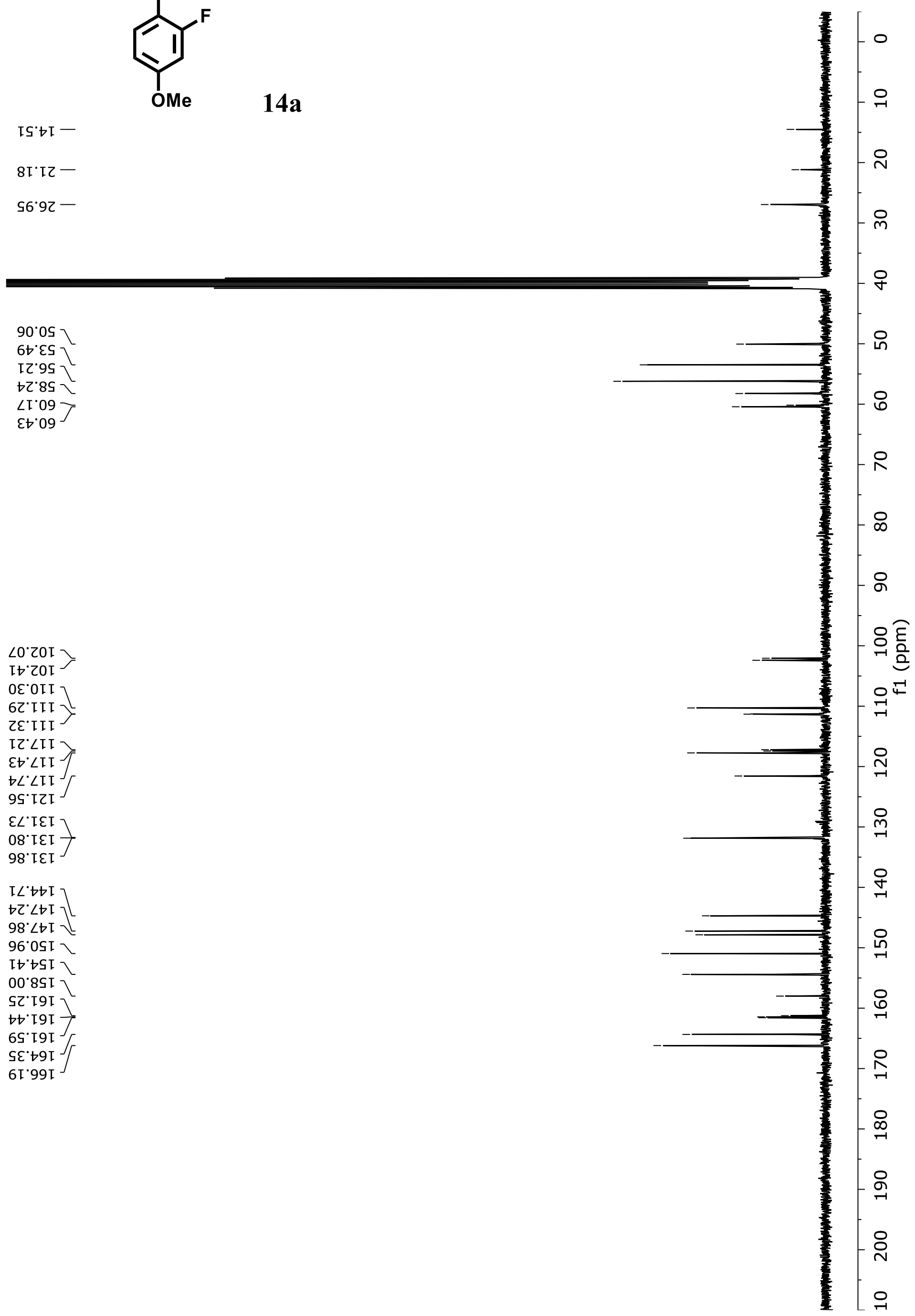

^{1}H spectrum of 14b

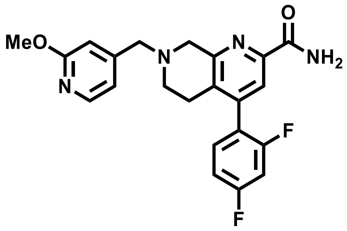

14b

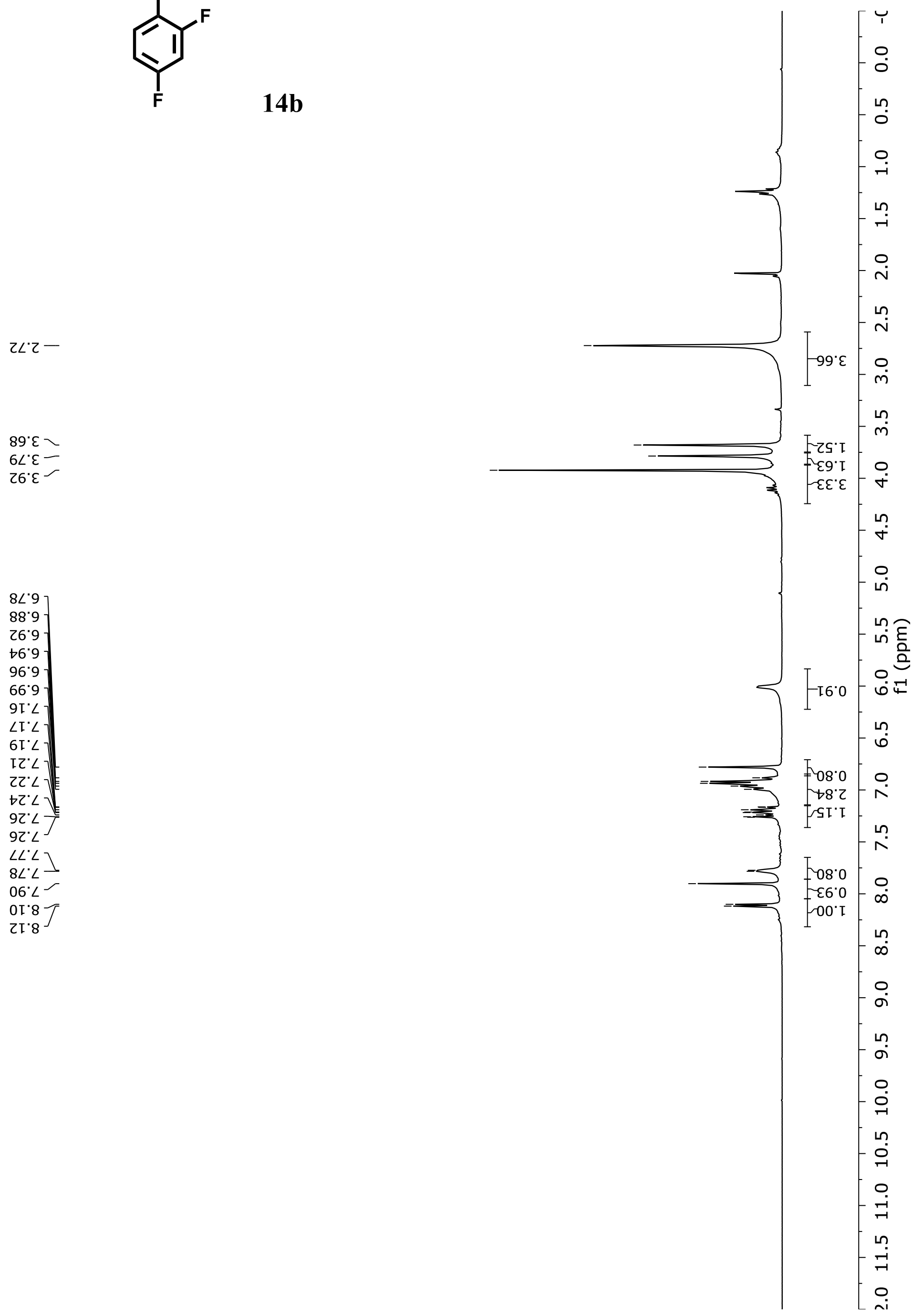

^{13}C spectrum of 14b

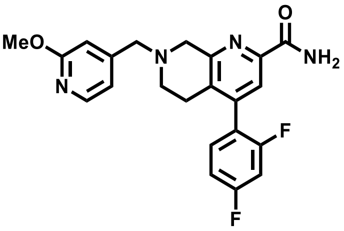

14b

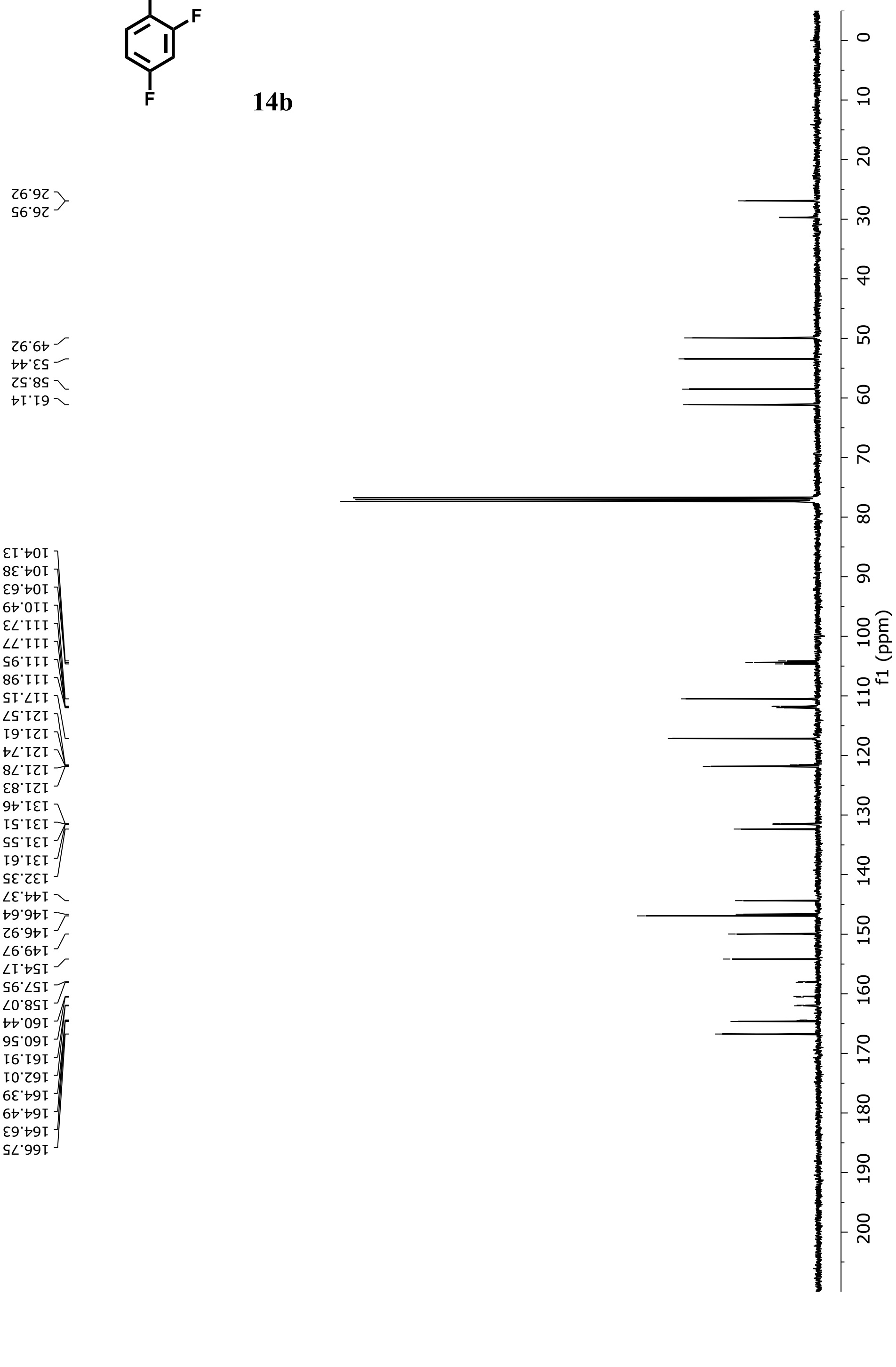

^{1}H spectrum of 14c

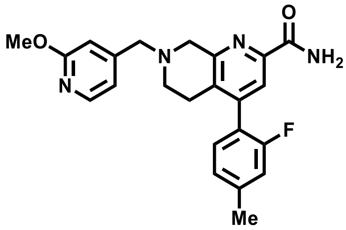

14c

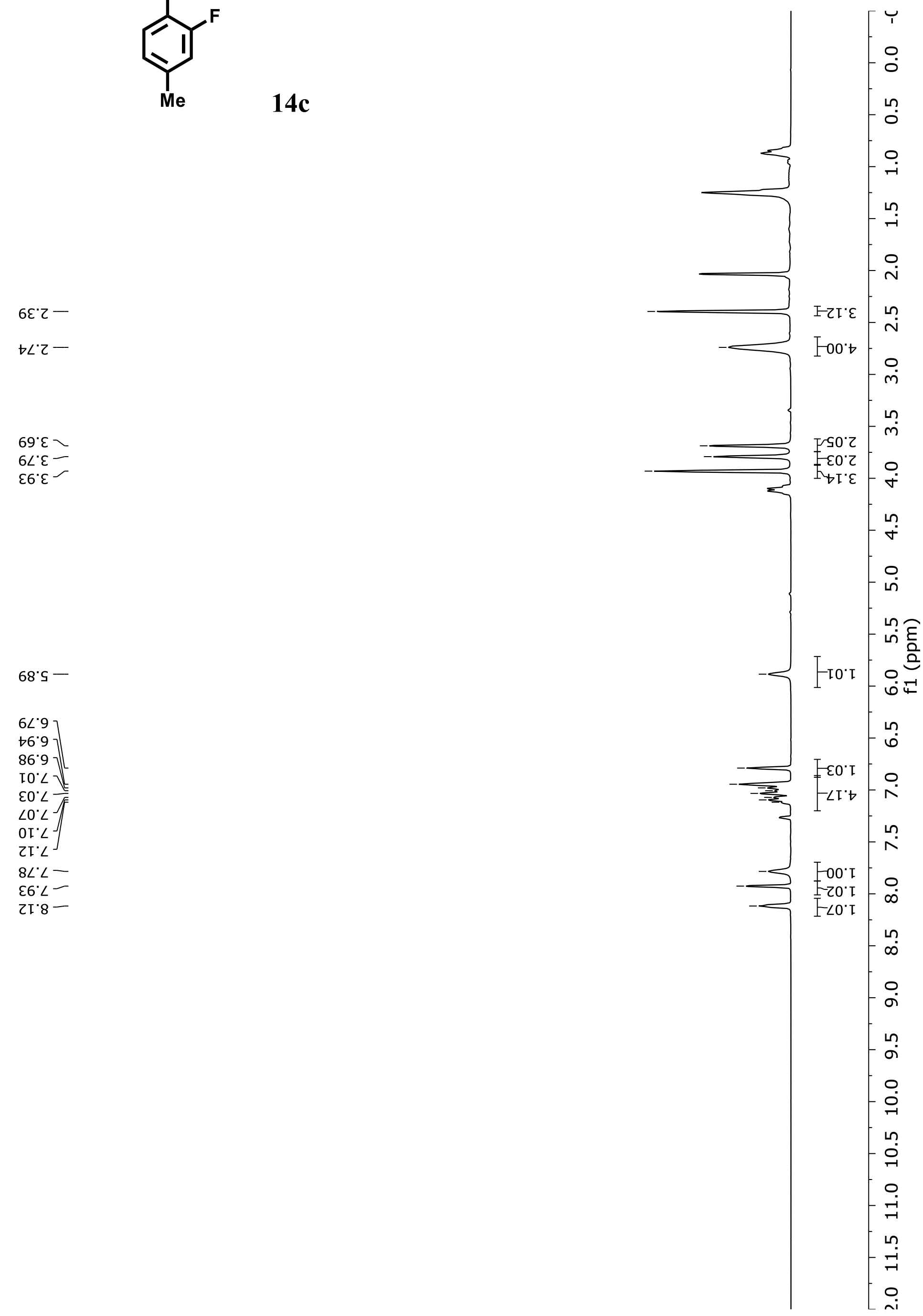

^{13}C spectrum of 14c

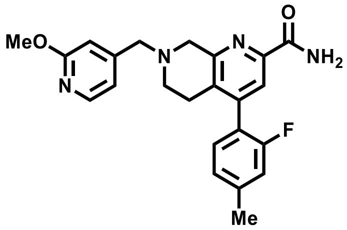

14c

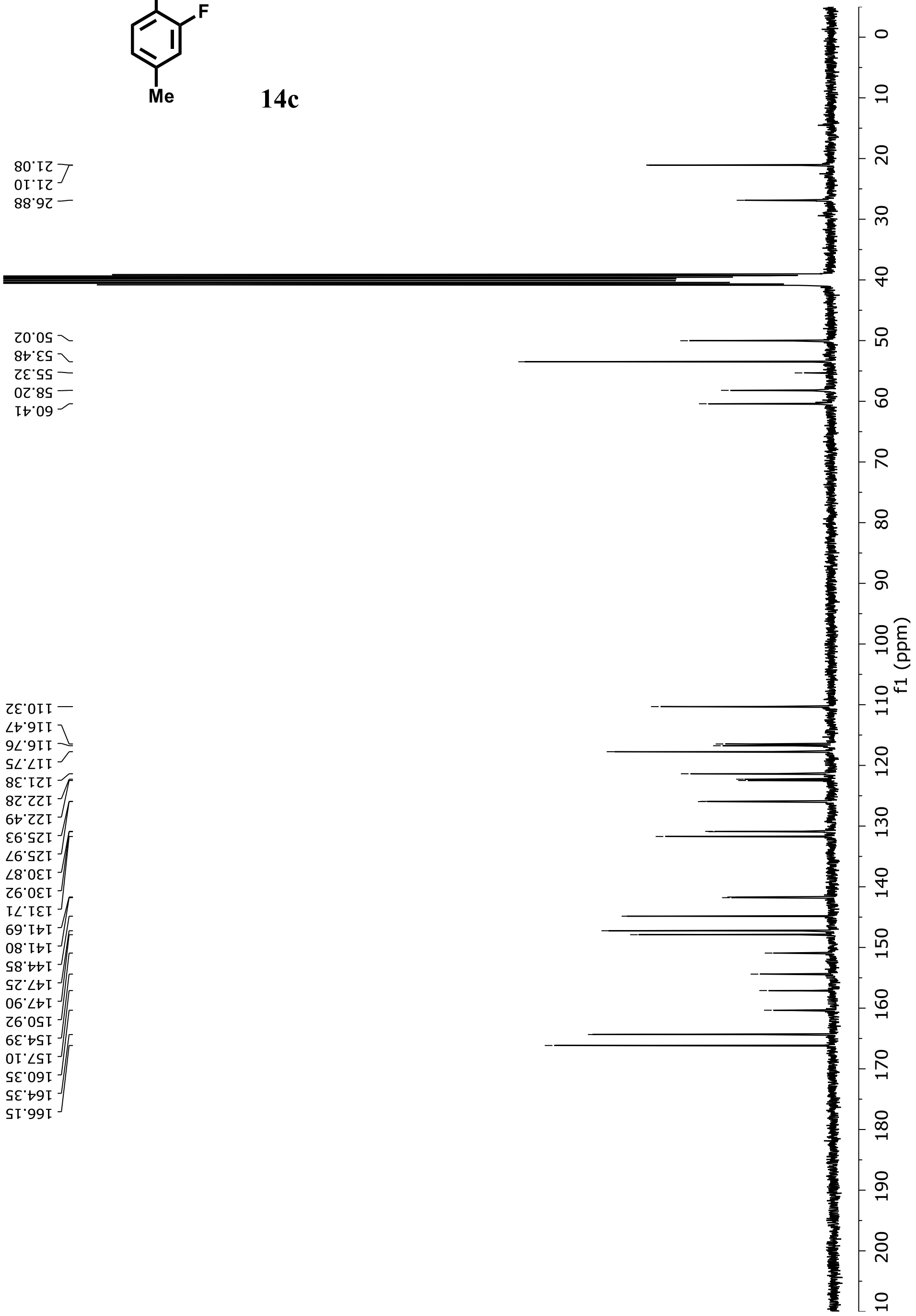

^{1}H spectrum of 14d

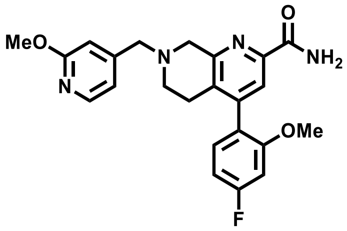

14d

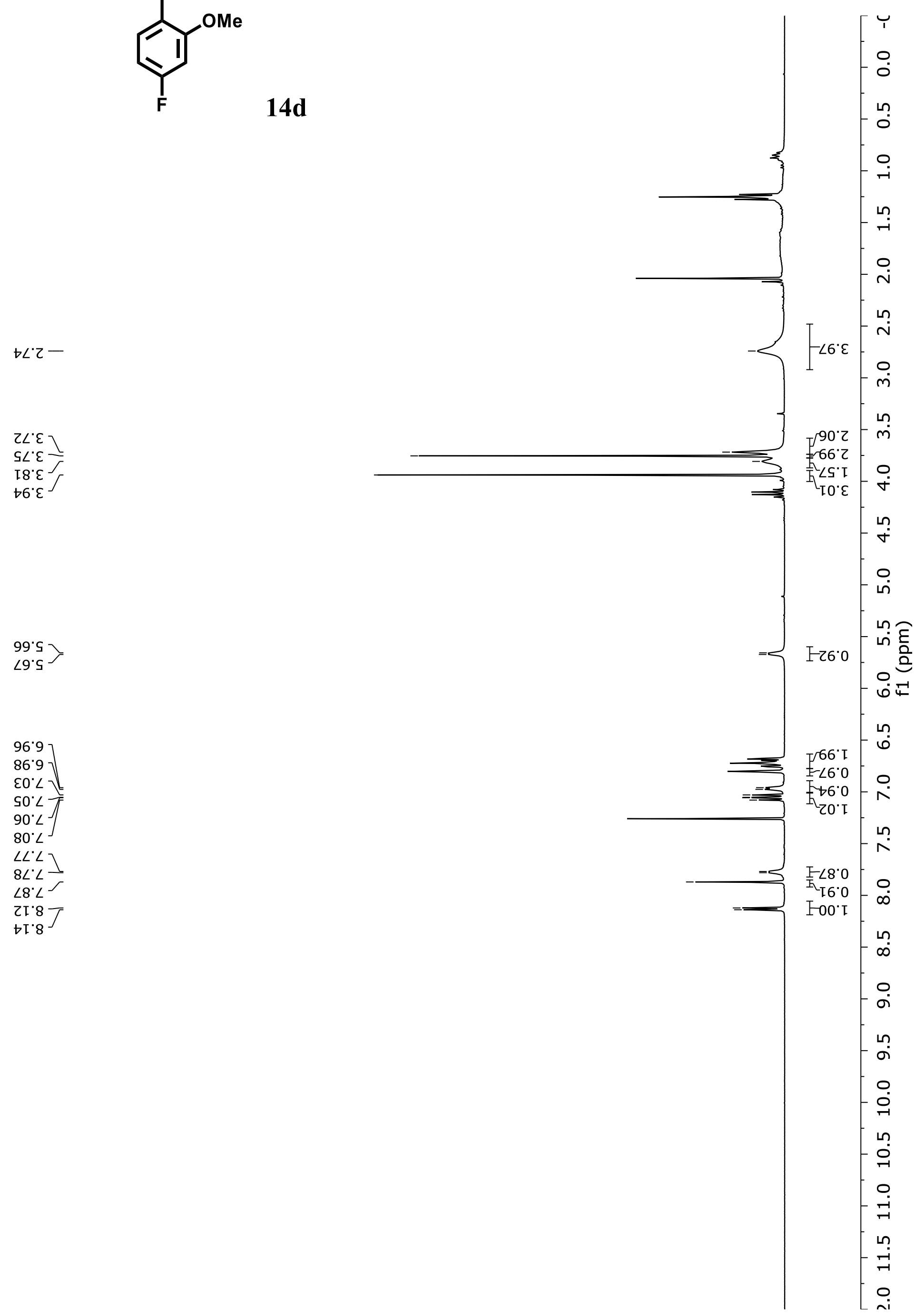

^{13}C spectrum of 14d

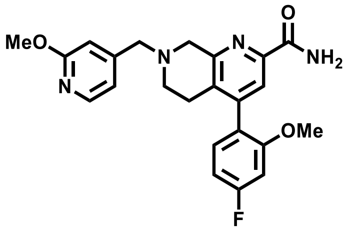

14d

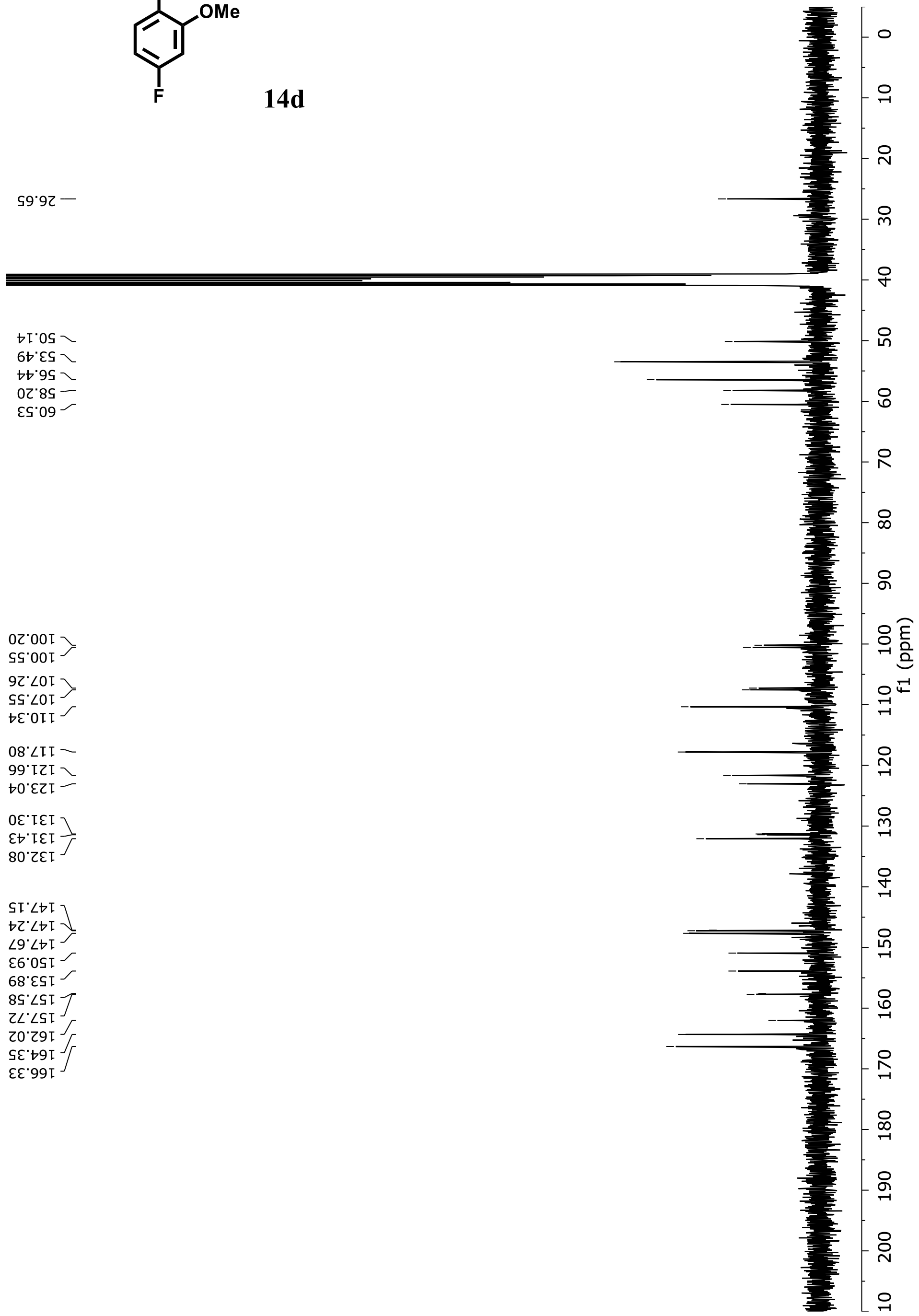

^{1}H spectrum of 14e

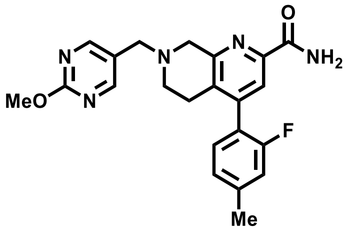

14e

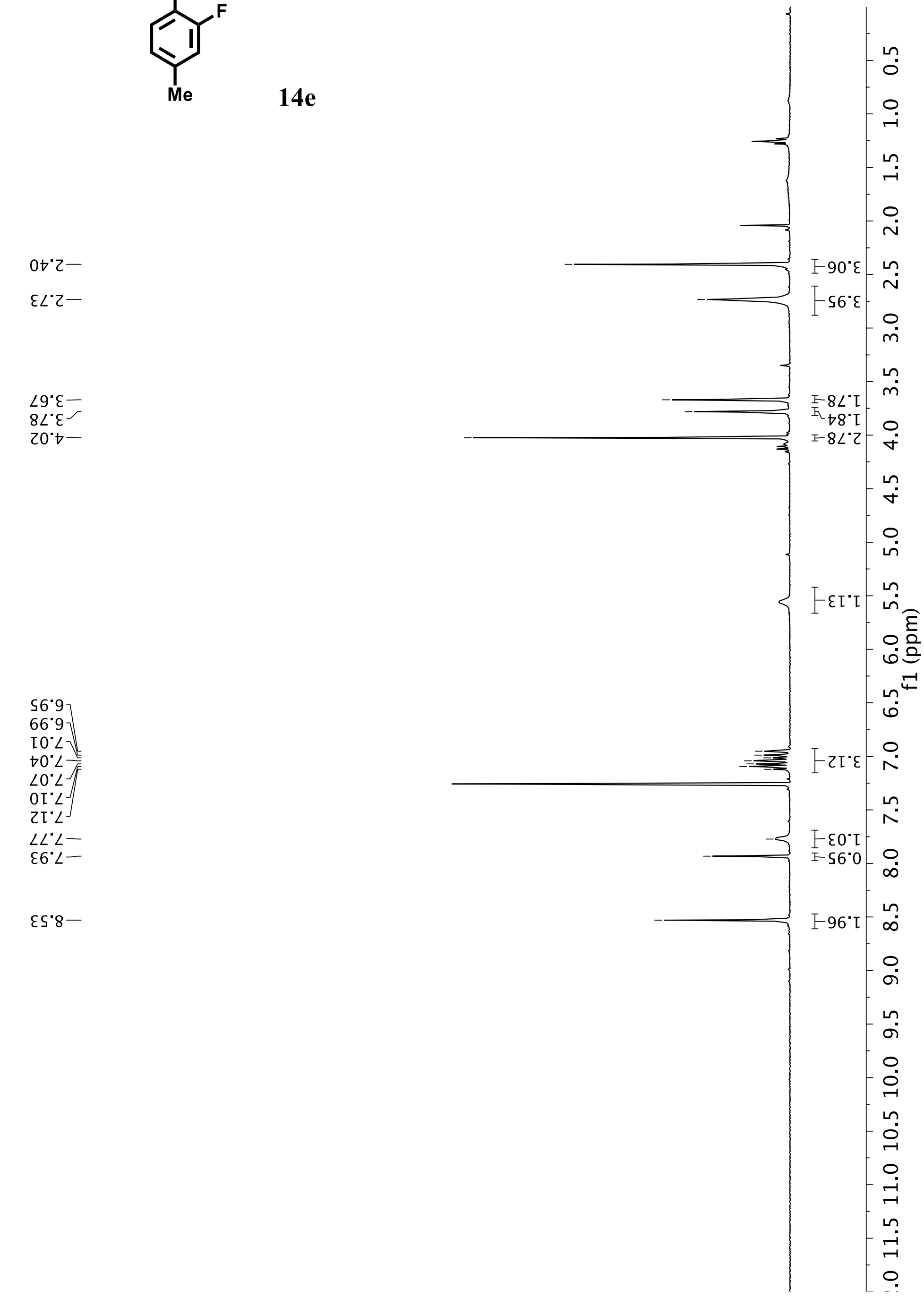

^{13}C spectrum of 14e

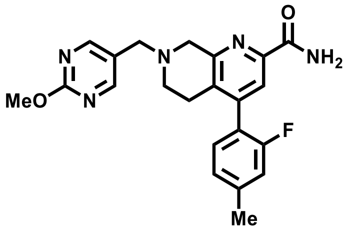

14e

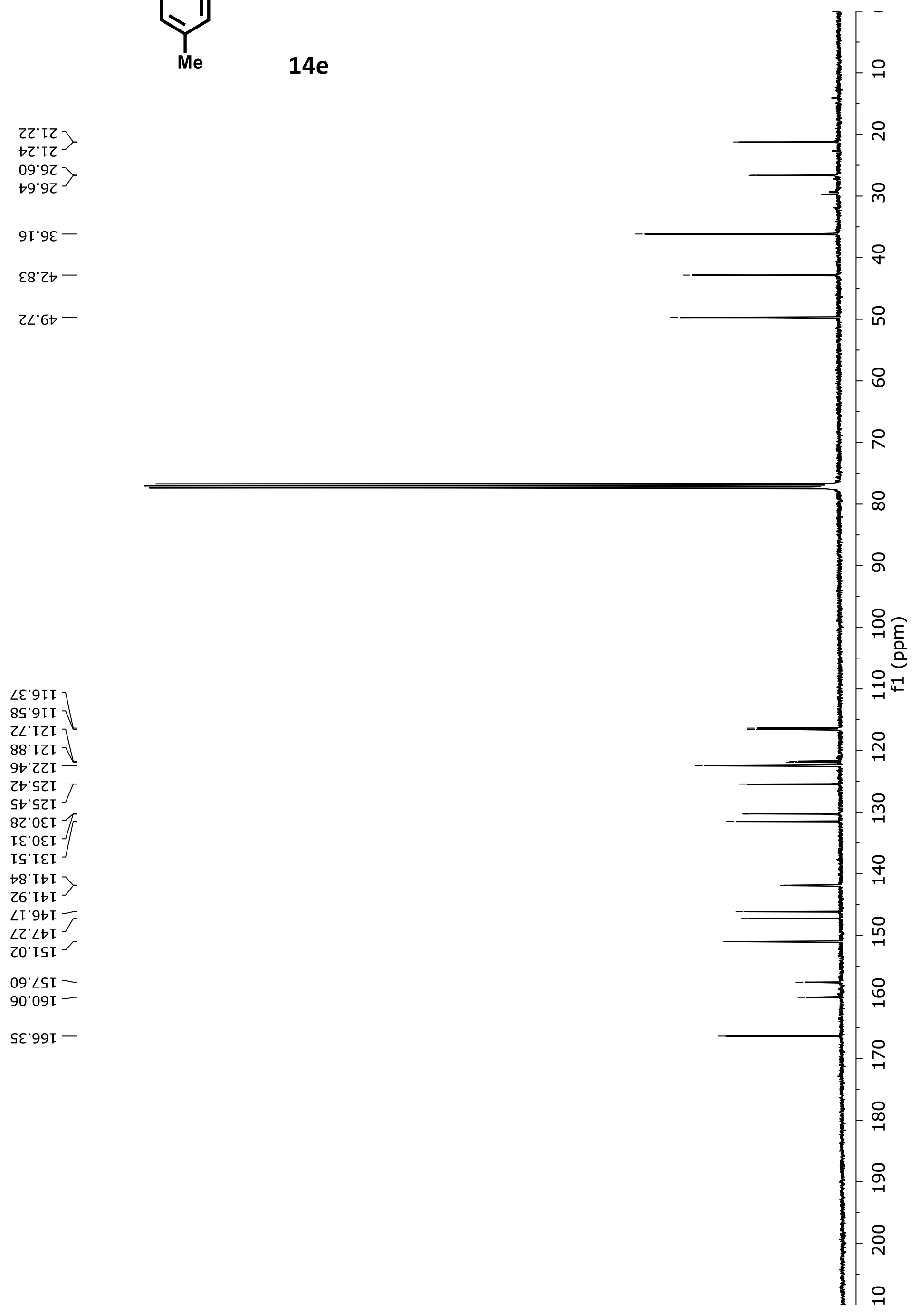

^{1}H spectrum of 14f

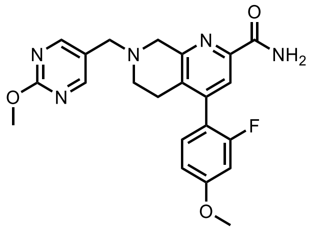

14f

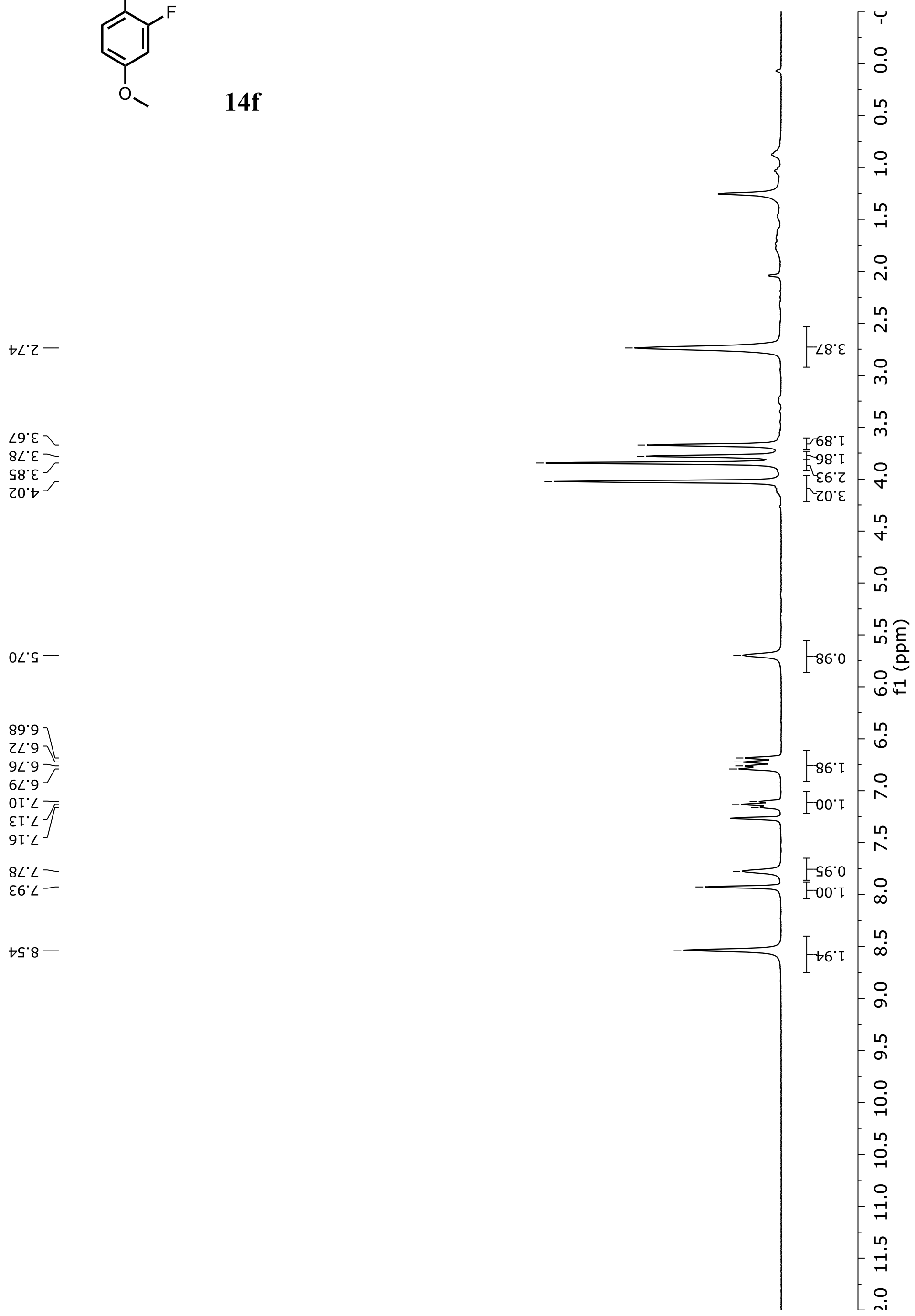

^{13}C spectrum of 14f

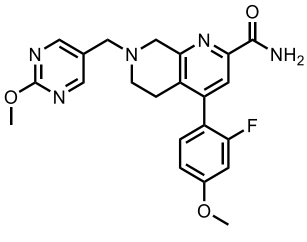

14f

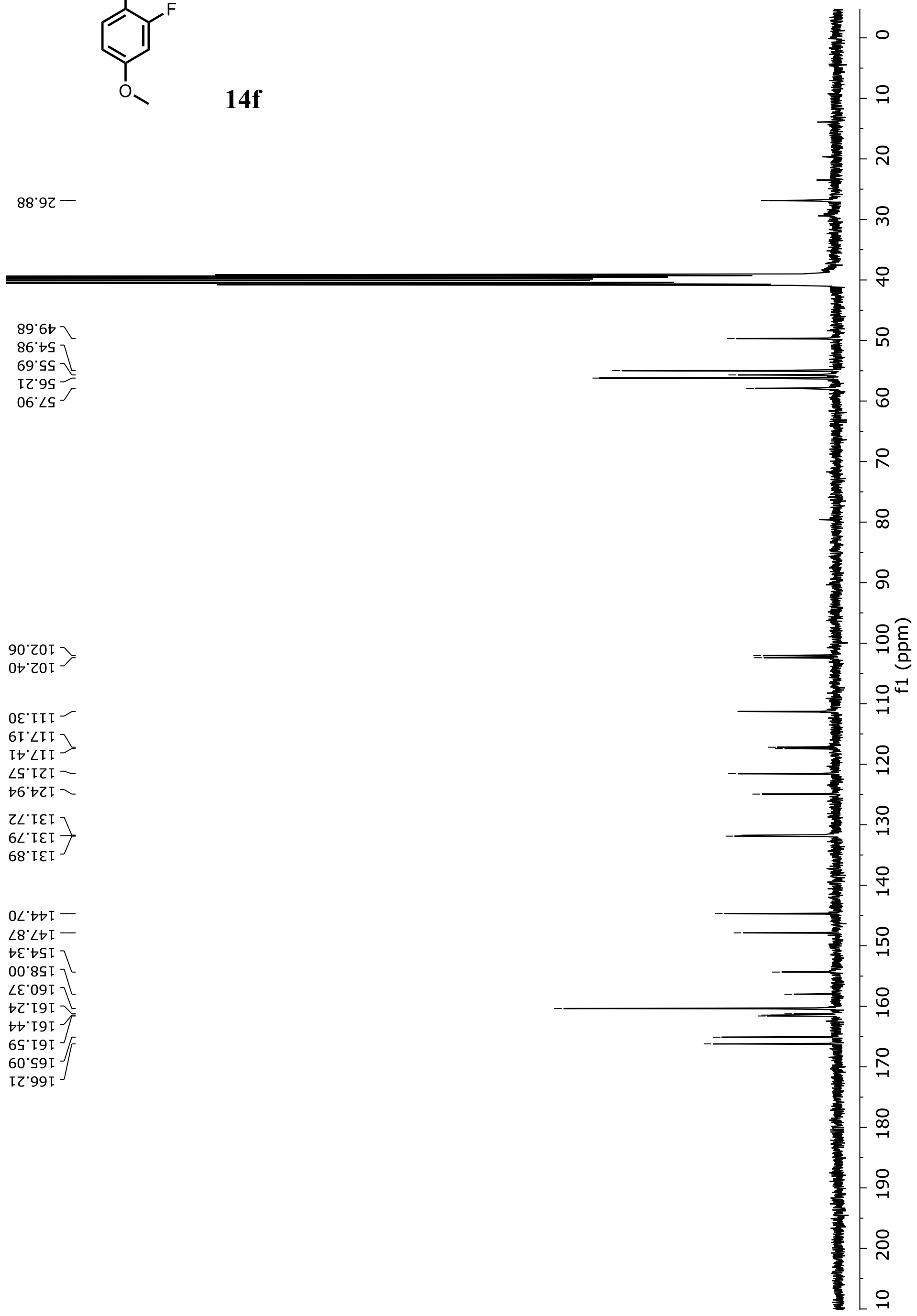

^{1}H spectrum of 14g

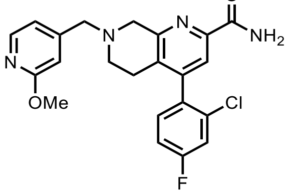

14g

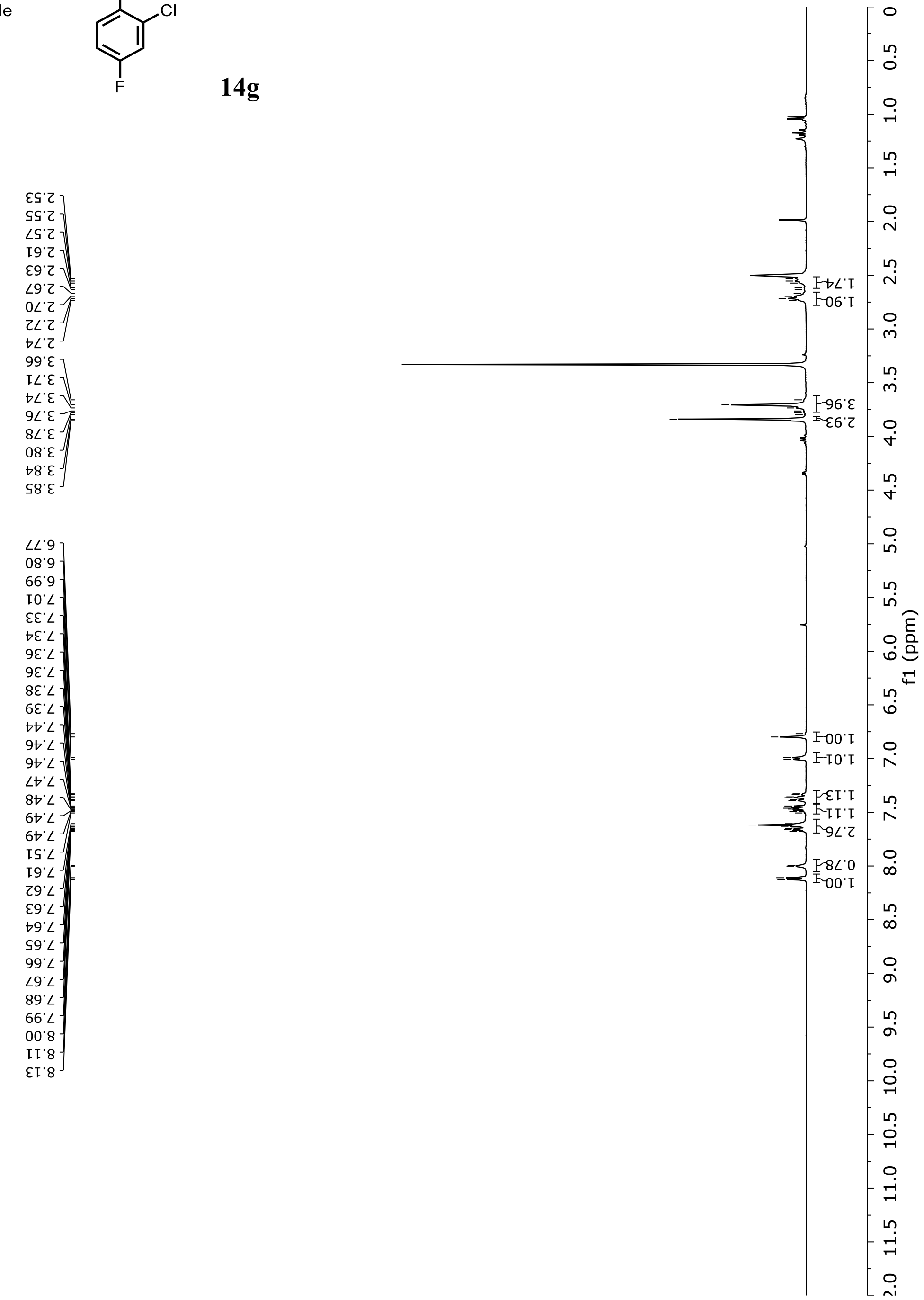

^{13}C spectrum of 14g

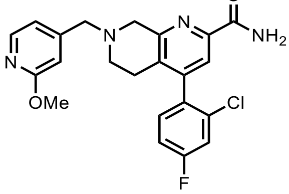

14g

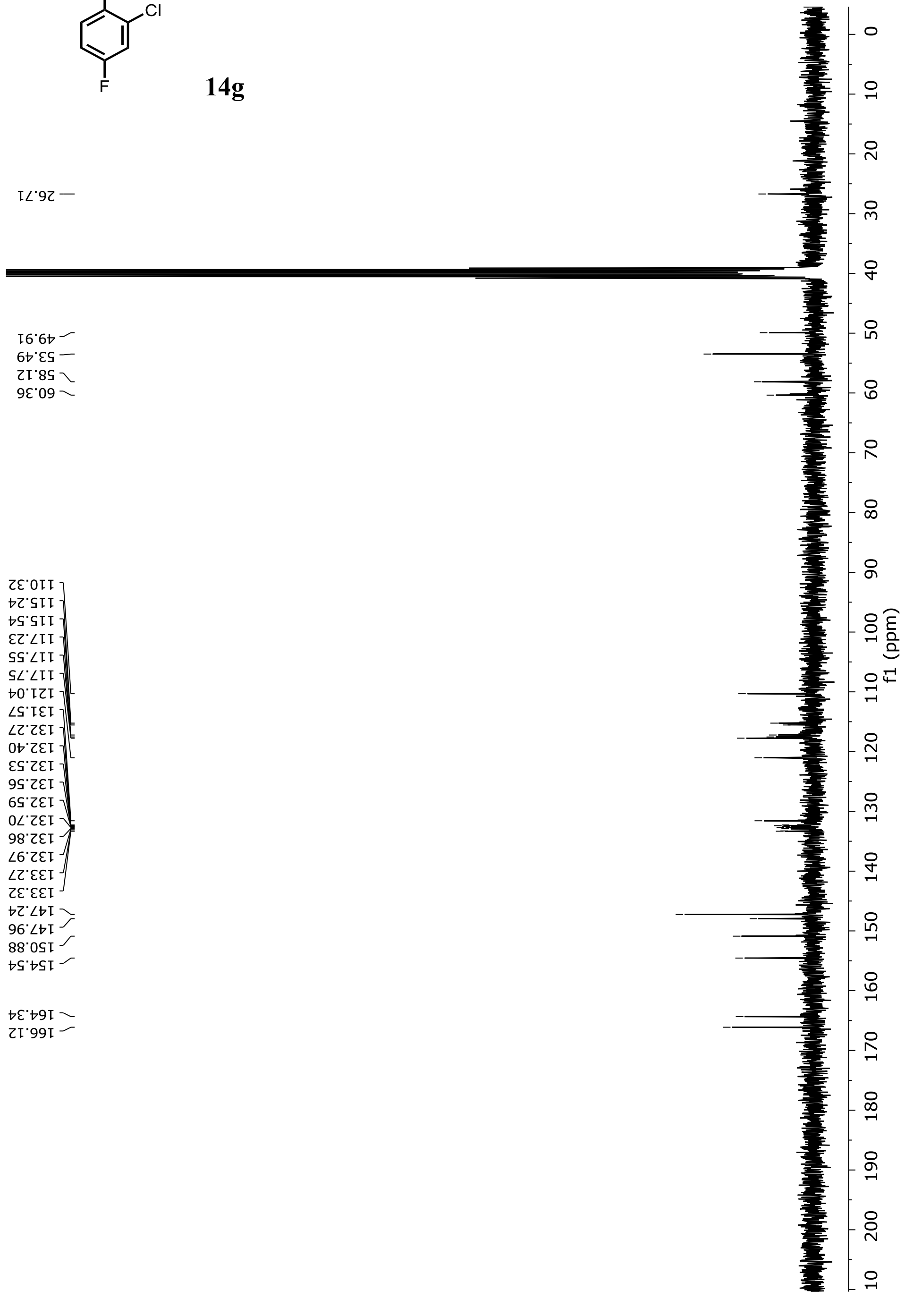

^{1}H spectrum of 16

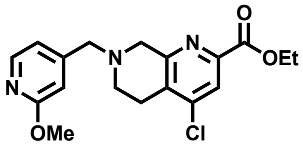

16

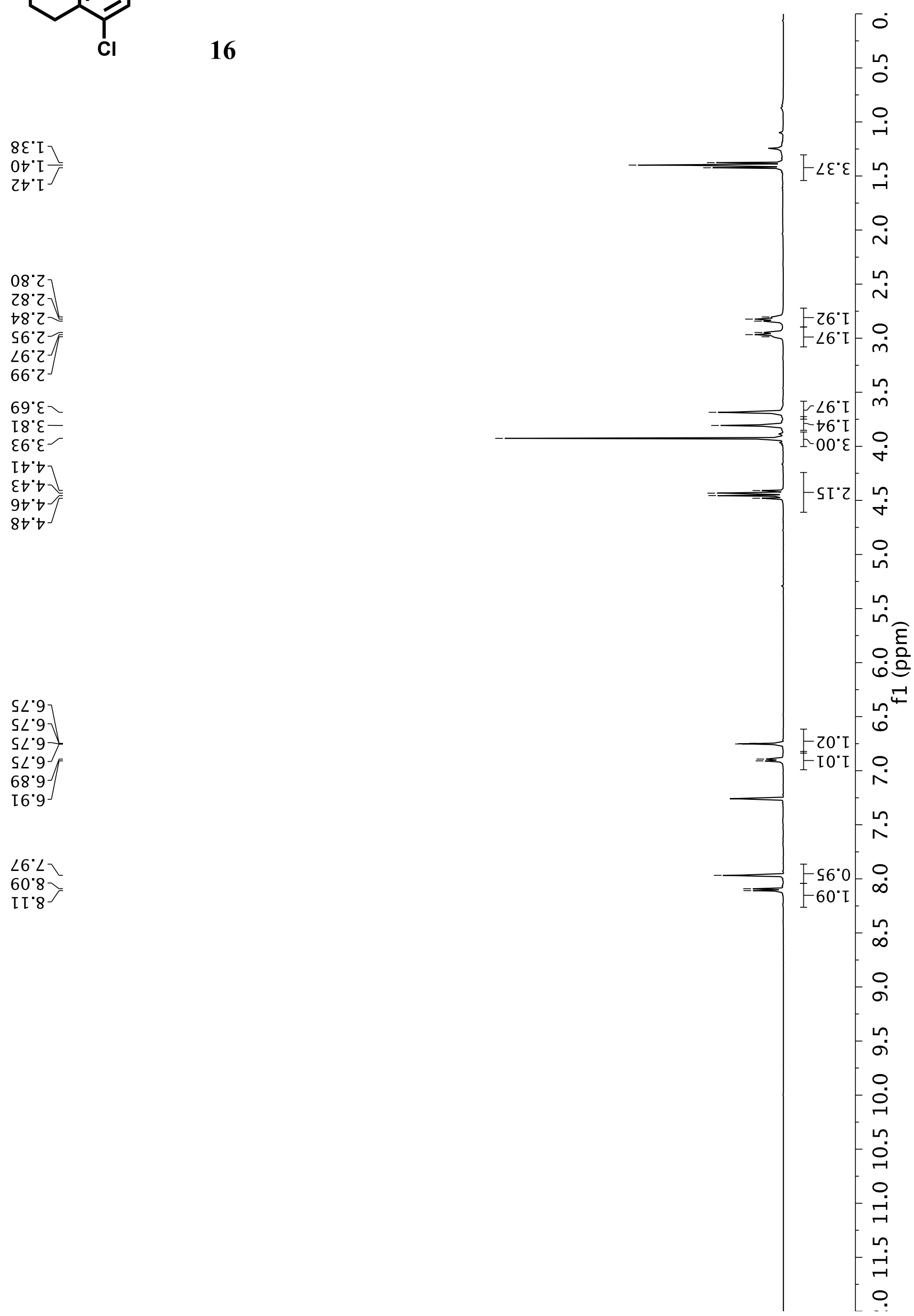

^{13}C spectrum of 16

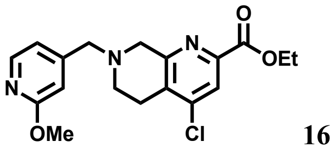

16

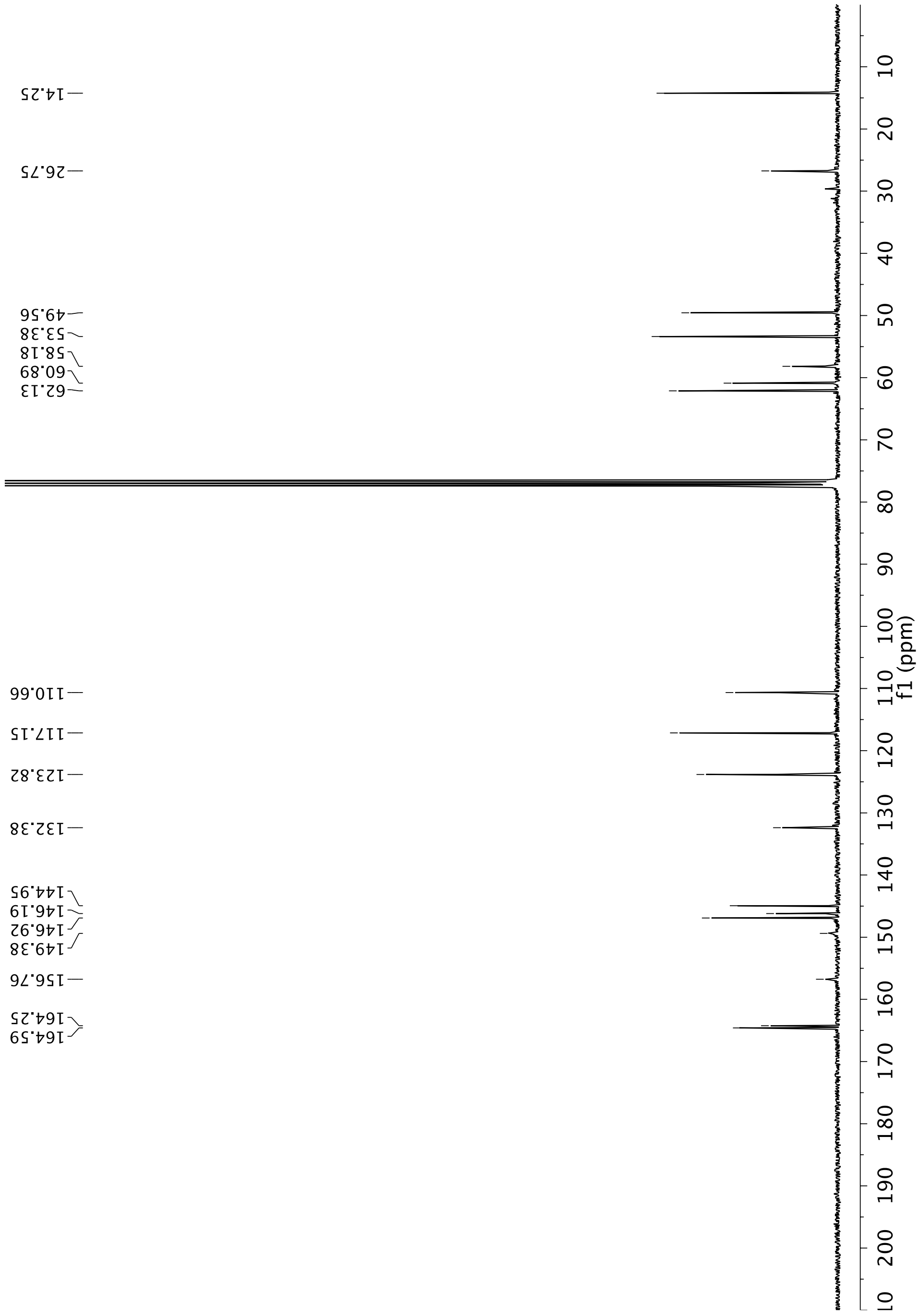

^{1}H spectrum of 17

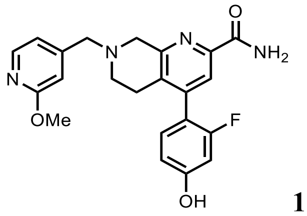

17

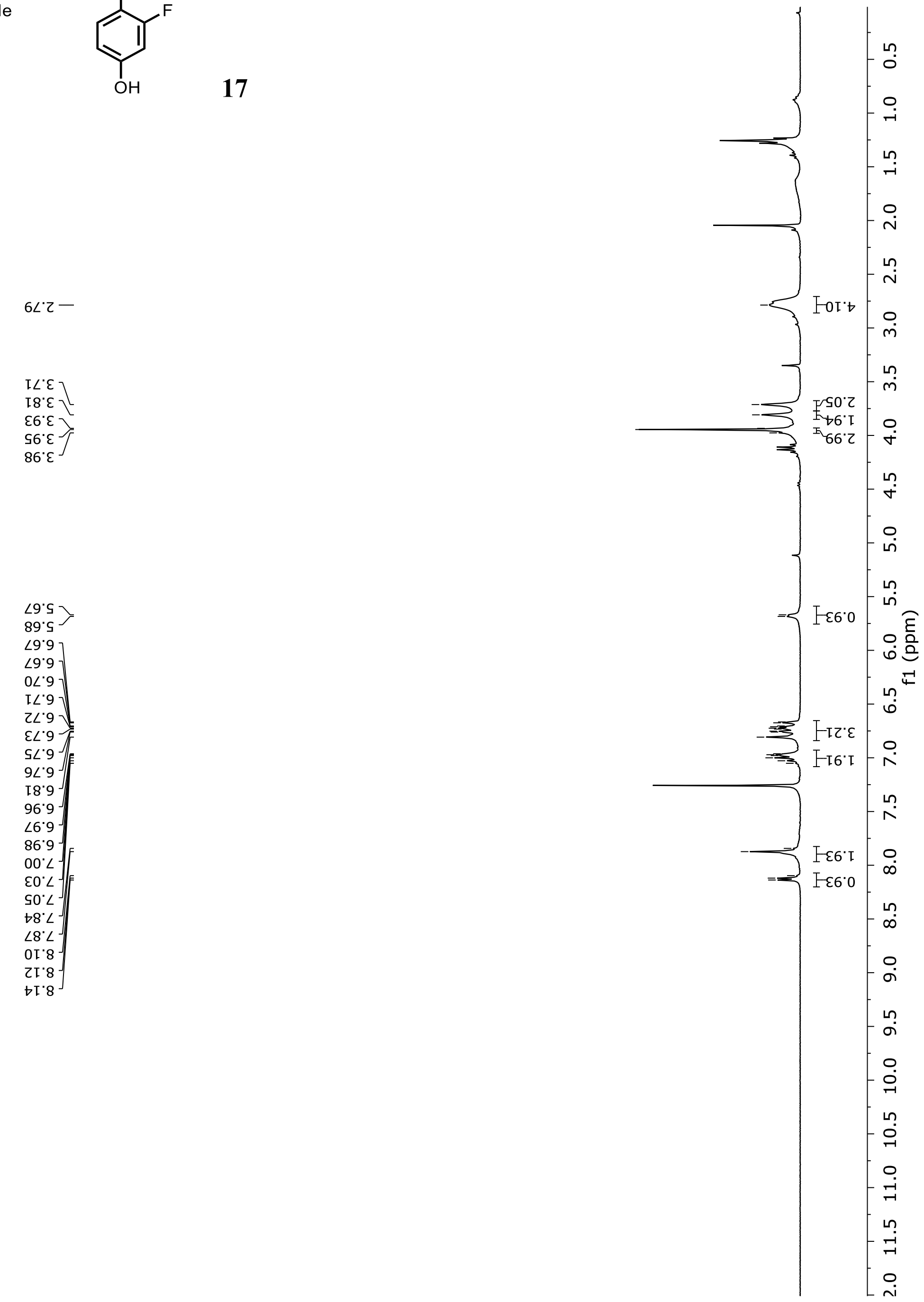

^{13}C spectrum of 17

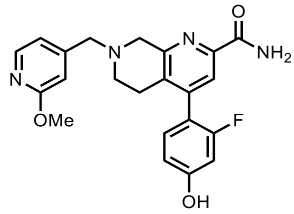

17

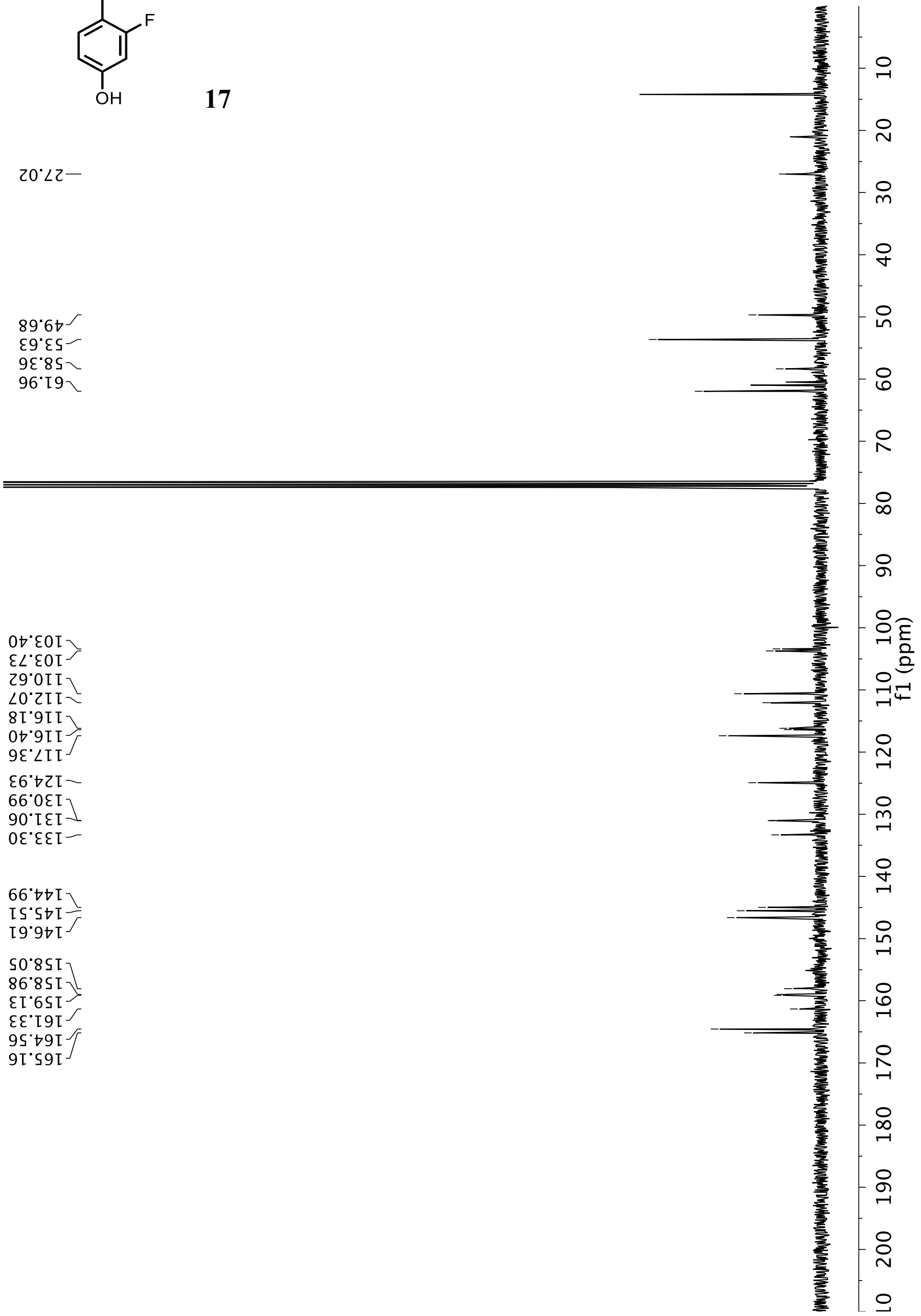

^{1}H spectrum of 18

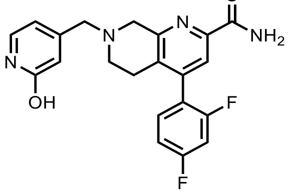

18

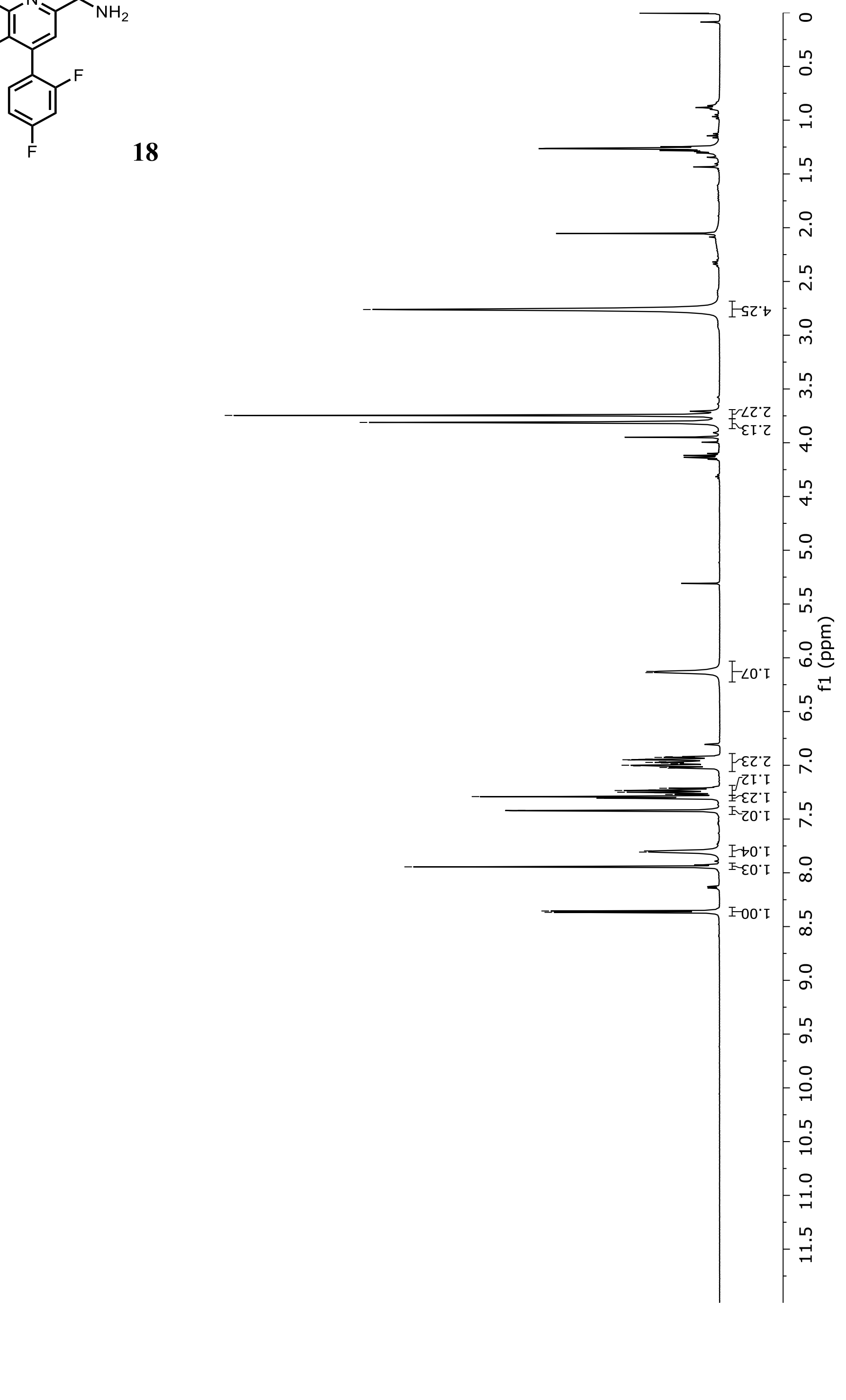

^{13}C spectrum of 18

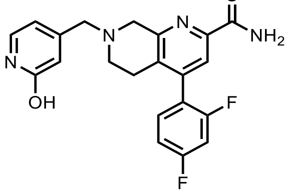

18

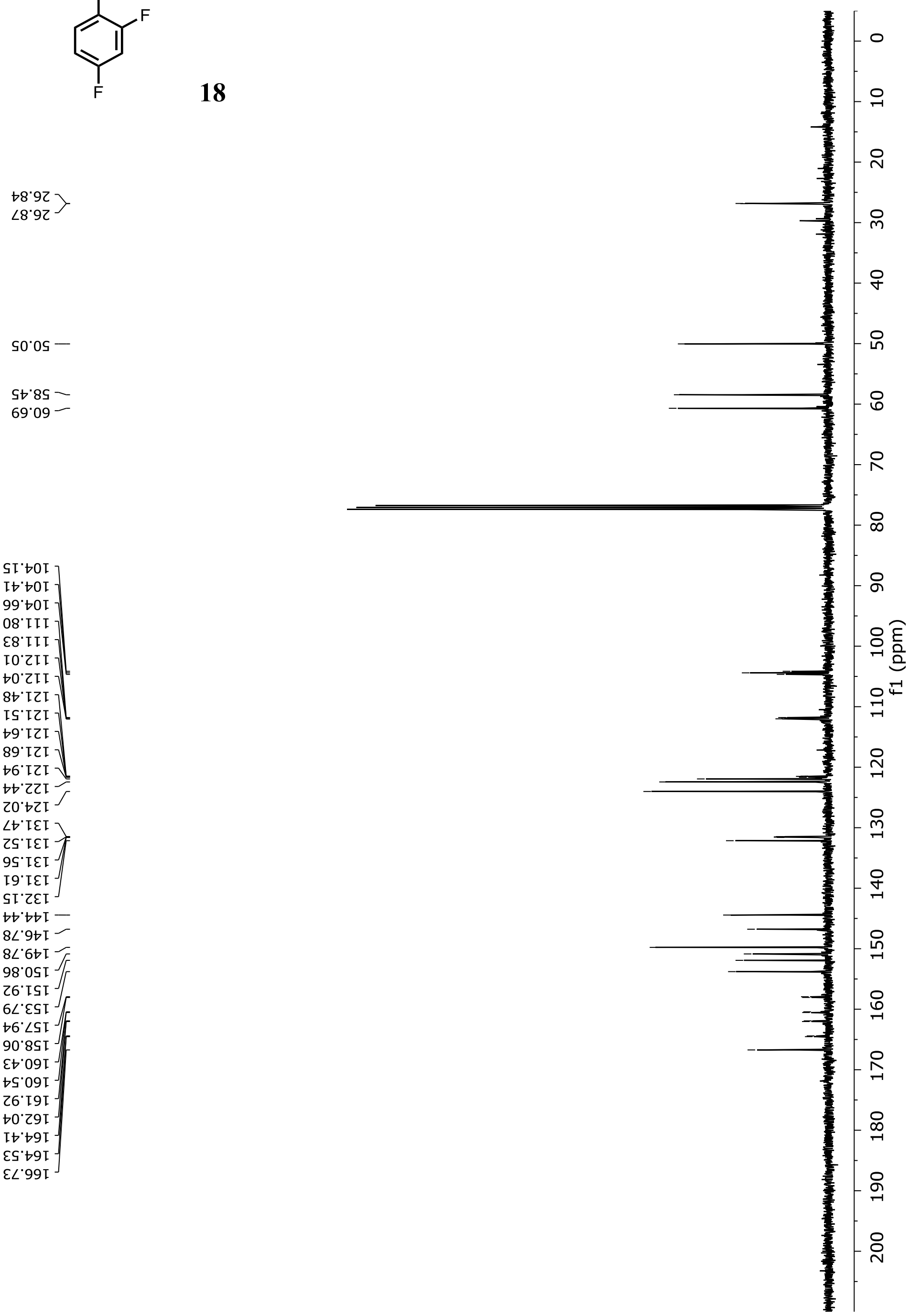

Supplement: Supplementary file 1 — Supplementary figures and tables. [file thnov10p11178s1.pdf]
